# Phenanthrene Monomers and Dimers from *Juncus tenuis* with Antiproliferative Activity and Synergistic Effect with Doxorubicin Against Human Colon Cancer Cell Lines

**DOI:** 10.3390/ijms26167665

**Published:** 2025-08-08

**Authors:** Anita Barta, Annamária Kincses, Dragica Purger, Gabriella Spengler, Judit Hohmann, Andrea Vasas

**Affiliations:** 1 Institute of Pharmacognosy, Interdisciplinary Excellence Centre, University of Szeged, 6720 Szeged, Hungary; bartaanita96@gmail.com (A.B.); kincses.annamaria@szte.hu (A.K.); hohmann.judit@szte.hu (J.H.); 2 HUN-REN-USZ Biologically Active Natural Products Research Group, University of Szeged, 6720 Szeged, Hungary; 3 Department of Pharmacognosy, University of Pécs, 7624 Pécs, Hungary; dragica@gamma.ttk.pte.hu; 4 Department of Medical Microbiology, Albert Szent-Györgyi Health Center, Albert Szent-Györgyi Medical School, University of Szeged, 6725 Szeged, Hungary; spengler.gabriella@med.u-szeged.hu

**Keywords:** *Juncus tenuis*, Juncaceae, phenanthrene, antiproliferative, doxorubicin, combination assay, synergism

## Abstract

Continuing our search for bioactive compounds in species from the Juncaceae family, we investigated *Juncus tenuis*. The structures of five previously undescribed phenanthrenes—tenuins A–E (**1**–**5**)—and 14 known phenanthrenes (**6**–**19**), along with other components, were isolated and characterized using nuclear magnetic resonance and high-resolution mass spectrometry measurements. The antiproliferative activity of all of the isolated phenanthrenes was evaluated against the human colorectal adenocarcinoma cell lines COLO 205 (doxorubicin-sensitive) and COLO 320 (doxorubicin-resistant), as well as a non-tumorigenic human fibroblast cell line (CCD-19Lu), using the MTT viability assay. Diphenanthrenes **4**, **5**, and **19** showed the most potent antiproliferative effects, with IC_50_ values ranging from 7.60 to 17.32 μM; however, these compounds lacked selectivity toward cancer cells. To explore potential chemosensitizing properties, the synergistic effects of the phenanthrenes with the anticancer drug doxorubicin were also examined in the COLO 320 cells. Notably, compound **2** exhibited very strong synergism (CI = 0.021), indicating a highly potent interaction. These findings highlight *J. tenuis* as a valuable source of phenanthrenes and demonstrate the synergistic anticancer potential of natural phenanthrenes with doxorubicin, offering promising prospects for overcoming multidrug resistance in colorectal cancer therapy.

## 1. Introduction

Phenanthrenes, a class of specialized secondary metabolites, are increasingly recognized for their diverse biological activities and structural uniqueness [1,2]. Among the plant families producing phenanthrenes, Juncaceae (the rush family) is one of the richest sources of these compounds [3]. The wide distribution of phenanthrenes is concentrated mainly in the genus *Juncus* within the family, with the structures ranging from simple monophenanthrenes, substituted with methyl, hydroxyl, and vinyl groups, to complex derivatives with additional functional groups. These compounds exhibit remarkable biological activities, including antioxidant, anti-inflammatory, antimicrobial, and cytotoxic effects, underscoring their potential as therapeutic agents [4]. Besides their pharmacological effects, phenanthrenes are hypothesized to play ecological roles in plant defense and stress tolerance [5]. However, to date, only a limited number of *Juncus* species (*n* = 13) have been investigated from phytochemical and pharmacological points of view. The most common phenanthrenes are juncunol, juncusol, effusol, juncuenin B, and dehydrojuncusol, of which juncusol can be considered a ubiquitous compound, as it has been isolated almost from all investigated species [4]. Dimeric phenanthrenes are rare in this genus; to date, most have been isolated from *J. acutus*, and seven of them have unusual hepta- and octacyclic structures [6,7,8]. Many *Juncus* species remain unexplored, leaving a wealth of potential bioactive compounds undiscovered.

Several phenanthrenes isolated from *Juncus* species have been screened for their in vitro cytotoxic activity in various cancer cell lines. First, juncusol was investigated, which proved to be active against NCI 90 KB (human epidermoid nasopharynx carcinoma) cells with an ED_50_ of 0.3 μg/mL [9]. The effects of effusol, dehydroeffusol, juncusol, dehydrojuncusol, juncuenin B, dehydrojuncuenin B, and juncuenin D on cell survival in HT22 cells were tested in a mouse hippocampal neuroblastoma cell line at 10, 30, and 100 µM for 24 h using the MTT assay. Among them, effusol, juncusol, juncuenin B, dehydrojuncuenin B, and juncuenin D resulted in a decrease in MTT reduction (9.9, 25.4, 13.6, 8.4, and 23.7%, respectively) at 100 µM and destroyed neuronal integrity [4,10].

With almost 2 million new cases and 1 million deaths worldwide in 2020, colorectal cancer ranks as the third and second most frequent cause of cancer incidence and mortality, respectively [11]. Doxorubicin is a commonly used chemotherapeutic drug in this type of human cancer as a result of its wide range of pharmacological activities, but at the same time, it causes a wide range of side effects (cardiotoxicity, neuropathy, hepatotoxicity, nephrotoxicity, alopecia, myelosuppression, neutropenia, anemia, and thrombocytopenia) [12,13]. In addition, common problems with monotherapy include drug resistance at the cellular and tumor levels; therefore, combination chemotherapy regimens containing two or more classical anticancer drugs have been applied for decades in clinical practice to treating a variety of cancers [14].

*Juncus tenuis*, commonly known as path rush, is a 10–80 cm height, widespread species in various ecological habitats, including disturbed soils and wetlands. It occurs in North America and Western and Central Europe, as well as East Asia, Australia, and New Zealand [15,16]. Despite its broad distribution and ecological adaptability, *J. tenuis* has been underexplored for its secondary metabolites. Previously, three phenanthrenes, namely effusol, juncusol, and 2,7-dihydroxy-1,8-dimethyl-5-vinyl-9,10-dihydrophenanthrene, were isolated from this plant and their antiproliferative activity tested against different tumor (A2780, A2780cis, KCR, MCF-7, HeLa, HTB-26, and T47D) and normal (MRC-5) cell lines. Juncusol and effusol possessed high activity in HeLa cells, with IC_50_ values of 0.5 and 2.3 μM [17].

This study presents the isolation and structural elucidation of phenanthrenes from *J. tenuis* using advanced chromatographic and spectroscopic techniques. We further evaluated their pharmacological properties, focusing on their antiproliferative activity against the COLO 205 (doxorubicin-sensitive) and COLO 320 (-resistant) human tumor cell lines. We also tested the synergistic effect of phenanthrenes with the therapeutically used anticancer agent doxorubicin. This work aims to expand our chemical and biological understanding of the phenanthrenes in *J. tenuis*. It contributes to the broader exploration of the Juncaceae family as a reservoir of bioactive natural products.

## 2. Results and Discussion

**Figure 1:**
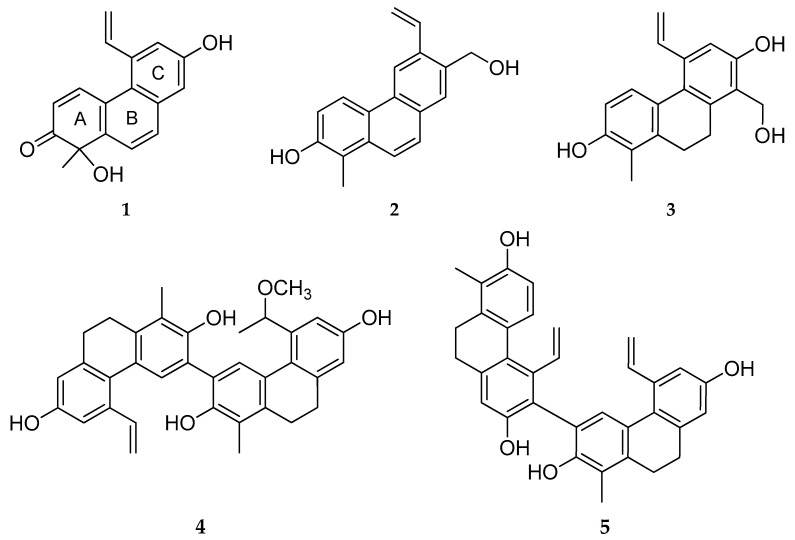
Structures of new compounds (**1**–**5**) isolated from *J. tenuis*.

Air-dried aerial parts of *J. tenuis* were ground into a powder and extracted using MeOH. After concentration, the extract was dissolved in 50% aqueous MeOH, and *n*-hexane, CHCl_3_, and EtOAc were used to perform solvent–solvent partitioning. Several chromatographic techniques [column chromatography (CC), vacuum liquid chromatography (VLC), Sephadex LH-20 gel chromatography, and high-performance liquid chromatography (HPLC)] were applied to isolating 24 compounds from the CHCl_3_ fraction, including 5 new (**1**–**5**) (Figure 1) and 14 known (**6**–**19**) phenanthrenes and 5 other compounds.

The structures of the compounds were elucidated using a comprehensive spectroscopic analysis, including one- and two-dimensional NMR (^1^H–^1^H COSY, HSQC, HMBC, NOESY) and high-resolution electrospray ionization mass spectrometry (HRESIMS) measurements, complemented by comparisons with previously reported spectral data.

### 2.1. Structural Elucidation of the Isolated Compounds

#### 2.1.1. Tenuin A (**1**)

Tenuin A (**1**) was isolated as a yellow amorphous solid, exhibiting an optical rotation of [α]_D_^26^ 0 (*c* 0.1, MeOH). HRESIMS revealed its molecular formula to be C_17_H_14_O_3_, based on the observed [M–H]^−^ ion at *m*/*z* 265.0871 (calculated for C_17_H_13_O_3_^−^, 265.0870) (Figure S8). The ^1^H NMR spectrum (Table 1, Figure S1) showed resonances corresponding to two *ortho*-coupled aromatic protons [*δ*_H_ 6.13 d (1H, d, *J* = 10.4 Hz, H-3), 8.73 d (1H, d, *J* = 10.4 Hz, H-4)] and four aromatic protons as singlets [*δ*_H_ 7.09 (1H, s, H-8), 7.18 (1H, s, H-6), and 2×7.79 (1H, s, H-9,10)]; one methyl singlet at *δ*_H_ 1.55 (3H, s, Me-11); and a vinylic system at *δ*_H_ 5.69 d (17.3 Hz), 5.44 d (10.8 Hz) (H-13), and 7.35 dd (17.3, 10.7) (H-12). In the JMOD spectrum, 17 carbon signals were detected (Table 1, Figure S2).

**Figure 2:**
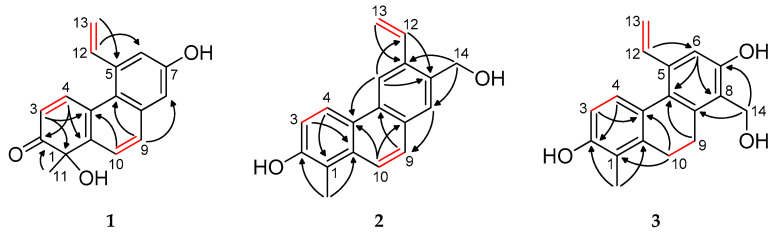

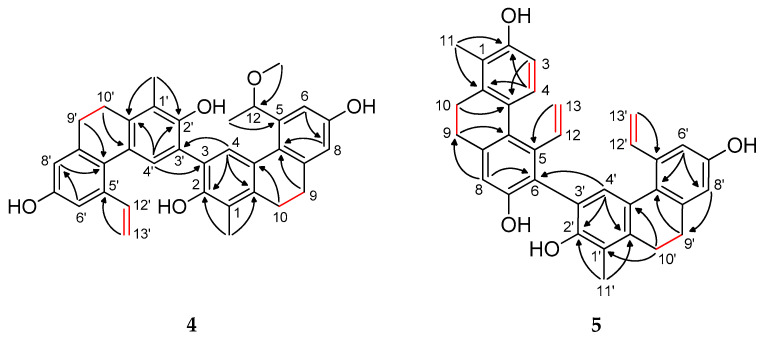
^1^H-^1^H COSY (–) and diagnostic HMBC (H→C) correlations of compounds **1**–**5**.

The resonance of a singlet aromatic proton at *δ*_H_ 7.79 (2H) was observed, which correlated in the HSQC spectrum (Figure S3) with carbon signals at *δ*_C_ 131.6 and 124.7 and was attributed to protons H-9 and H-10 in a phenanthrene scaffold. The ^1^H–^1^H COSY spectrum revealed three distinct spin systems: H-3/H-4 (*δ*_H_ 6.13, d and 8.73, d), H-12/H-13a (*δ*_H_ 7.35, dd and 5.69, d), and H-12/H-13b (*δ*_H_ 7.35, dd and 5.44, d) (Figure S4). A signal at *δ*_C_ 206.9 in the ^13^C JMOD NMR spectrum indicated the presence of a carbonyl group, which was assigned to C-2 based on the HMBC correlations from both the methyl protons of CH_3_-11 (*δ*_H_ 1.55, s) and H-4 (*δ*_H_ 8.73, d) to C-2 (δC 206.9) (Table 1, Figure 2 and Figure S5). The *ortho*-coupled doublets at *δ*_H_ 6.13 and 8.73 were assigned to H-3 and H-4, respectively, supported by long-range correlations: H-3 with C-1 and C-4a and H-4 with C-1a. The methyl group at *δ*_H_ 1.55 (s) was located at C-1, as confirmed by its HMBC cross-peaks with quaternary carbons at *δ*_C_ 146.7 (C-1a), 78.3 (C-1), and 206.9 (C-2). The attachment of hydroxy groups to C-1 and C-7 was inferred from the chemical shifts in the quaternary carbons at *δ*_C_ 78.3 (C-1) and *δ*_C_ 156.2 (C-7), respectively. Furthermore, HMBC correlations from H-13a and H-13b to C-5 and from H-12 to C-6 indicated the presence of a vinyl group at position C-5.

**Figure 3:**
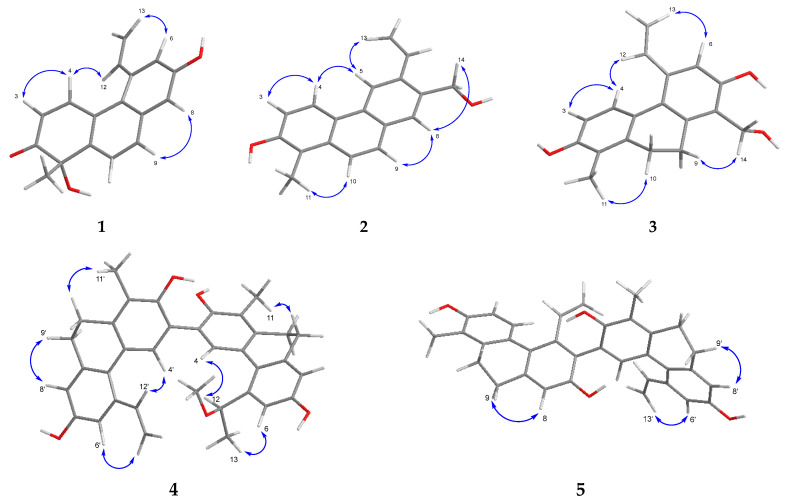
Key NOESY correlations (↔) for compounds **1**–**5**.

The structure of compound **1** was corroborated further by NOESY data, which revealed key nuclear Overhauser enhancements between H-3/H-4, H-4/H-12, H-13a/H-6, and H-9/H-8 (Figure 3 and Figure S6). These findings conclusively established the planar structure of compound **1**, herein named tenuin A.

Tenuin A (**1**) is a chiral natural product; however, it exhibited zero specific rotation (*c* 0.1, MeOH). Therefore, a chiral HPLC analysis of **1** was performed, revealing only one peak on a Lux Amylose-1 column. This result can be attributed to the low enantioselectivity of the biosynthetic step, which has also been observed for other natural phenanthrenes [18].

Compound **1** is structurally similar to luzulin A, isolated previously from *Luzula luzuloides* (Juncaceae) [19]. Ring A is the same in the case of the two compounds, containing a chiral carbon atom (C-1) substituted with methyl and hydroxyl groups and a carbonyl group at C-2 (Figure 1).

#### 2.1.2. Tenuin B (**2**)

Compound **2** (tenuin B) was obtained as a light yellow amorphous solid. Its molecular formula was established as C_18_H_16_O_2_ according to the molecular ion at *m*/*z* 263.1078 [M–H]^−^ (calcd for C_18_H_15_O_2_^−^, 263.1067) in HRESIMS (Figure S16). Based on the ^1^H NMR spectrum (Table 1, Figure S9), this compound is also a vinyl-substituted phenanthrene. In addition, the ^1^H NMR spectrum indicated one aromatic methyl group (*δ*_H_ 2.55 s), four *ortho*-coupled aromatic protons (*δ*_H_ 7.22 d, 7.72 d, 7.90 d, 8.50 d), two *para*-coupled aromatic protons (*δ*_H_ 7.84 s, 8.75 s), and one methylene signal (*δ*_H_ 4.86 s). The ^13^C JMOD NMR spectrum (Table 1, Figure S10) confirmed the presence of 18 carbon atoms (2 bearing oxygen atoms), 8 quaternary carbons, 7 methine, 2 methylene, and a methyl carbon. These NMR and MS data suggested a phenanthrene skeleton similar to dehydrojuncuenin A isolated from *J. sethcuensis* and *J. inflexus* [20]. The difference between the two compounds is a hydroxymethyl group in **2** instead of the methyl group in dehydrojuncuenin A at C-7 (Figures S11 and S12). The substitution of **2** was determined through an HMBC experiment (Figure S13). The key correlations between H_3_-11 (*δ*_H_ 2.55, s)/C-1 (*δ*_C_ 118.9), C-2 (*δ*_C_ 154.7), C-1a (*δ*_C_ 133.9), H-5 (*δ*_H_ 8.75, s)/C-12 (*δ*_C_ 136.1), H-13b (*δ*_H_ 5.43, dd)/C-6 (*δ*_C_ 136.7), H_2_-14 (*δ*_H_ 4.86, s)/C-6 (*δ*_C_ 136.7), and C-8 (*δ*_C_ 128.7) suggested the methyl group at C-1, the vinyl substituent at C-6, and the hydroxymethyl group at C-7 (Figure 2).

The structure of compound **2** was further confirmed by the NOESY experiment (Figure S14). Overhauser effects were detected between H_3_-11/H-10, H-3/H-4, H-4/H-5, H-5/H-13a, H_2_-14/H-8, and H-8/H-9, confirming the planar structure of **2,** and it was named tenuin B (Figure 3).

#### 2.1.3. Tenuin C (**3**)

Tenuin C (**3**) was determined to possess the molecular formula C_18_H_18_O_3_, as established by its [M–H]^−^ peak at *m*/*z* 281.1184 in the HRESIMS (calculated for C_18_H_17_O_3_^−^, 281.1183) (Figure S24). The ^1^H and ^13^C JMOD NMR spectra (Figures S17 and S18) revealed features consistent with a 9,10-dihydrophenanthrene skeleton, evidenced by the methylene signals at *δ*_H_ 2.76 and 2.69 (each 2H, m) and the corresponding carbon signals at *δ*_C_ 26.9 and 26.5. The substitution pattern included one methyl group, two hydroxyl groups, a hydroxymethyl, and a vinyl moiety (Figures S19 and S20). Additionally, the ^1^H NMR spectrum displayed an isolated aromatic singlet at *δ*_H_ 6.92 (1H) and two *ortho*-coupled aromatic protons at *δ*_H_ 6.63 and 7.13 (each 1H, d, *J* = 8.4 Hz). The HMBC correlations of H_3_-11 with C-1 and C-2, H-3 with C-4a, and H-4 with C-2 confirmed the assembly of ring A (Figure 2 and Figure S21). The methylene protons H_2_-9 and H_2_-10 exhibited correlations with C-8a, C-1, C-1a, and C-4a, thereby connecting rings A and C. The vinyl group and the hydroxymethyl substituent were positioned at C-5 and C-8, respectively, as indicated by the HMBC cross-peaks from H-12 to C-6 and from H_2_-14 to both C-7 and C-8. Additional HMBC correlations from H-6 (*δ*_H_ 6.92) to C-5a, C-7 (*δ*_C_ 155.3), and C-8 (*δ*_C_ 124.4) supported the placement of a hydroxyl group at C-7, leading to the full structural assignment of compound **3** as 2,7-dihydroxy-8-hydroxymethyl-1-methyl-5-vinyl-9,10-dihydrophenanthrene. The NOESY spectrum further supported this structure by showing key spatial correlations between H_2_-9/H_2_-14, H-4/H-12, and H_2_-10/H_3_-11 (Figure 3 and Figure S22). Structurally, tenuin C (**3**) closely resembles 2,7-dihydroxy-1,8-dimethyl-5-vinyl-9,10-dihydrophenanthrene, previously reported from *Juncus effusus* and *Juncus acutus* [6]. The only structural difference lies in the substitution at C-8; in tenuin C (**3**), a hydroxymethyl group can be found instead of a methyl group at C-8.

#### 2.1.4. Tenuin D (**4**)

The molecular formula for compound **4** (tenuin D) is C_35_H_34_O_5_ based on the HRESIMS analysis (*m*/*z* 533.2330 [M–H]^−^, calcd for C_35_H_33_O_5_^–^, 533.2334) (Figure S32). Analysis of the ^1^H NMR, ^13^C NMR JMOD, HSQC and ^1^H–^1^H COSY spectra (Figures S25–S28) of this compound revealed a heterodimeric phenanthrenoid structure comprising two dihydrophenanthrene units (Table 2). Through an evaluation of the 2D NMR data, it was apparent that the building blocks of compound **4** are effususol A and effusol (**10**) [10]. The C-3–C-3′ connection of the two monomers was proved by observing the HMBC correlations of H-4 (*δ*_H_ 7.04 s) with C-3′ (*δ*_C_ 125.3) and H-4′ (*δ*_H_ 7.41 s) with C-3 (*δ*_C_ 125.2) (Figure 2 and Figure S29). The NOESY correlations between H-4/H-12, H-4′/H-12′, H-6/H-13a, and H-6′/H-13′a corroborated the elucidated structure (Figure 3 and Figure S30).

Compound **4** contains a stereogenic center at position C-12 within unit A. The specific optical rotation of the compound was measured as [α]_D_^26^ 0 (*c* 0.1, MeOH), indicating the absence of optical activity. The chiral HPLC analysis of tenuin D (**4**) revealed two well-resolved peaks of an equal area, each displaying identical UV spectra. These results indicate that compound **4** is a racemic mixture.

#### 2.1.5. Tenuin E (**5**)

**Figure 4:**
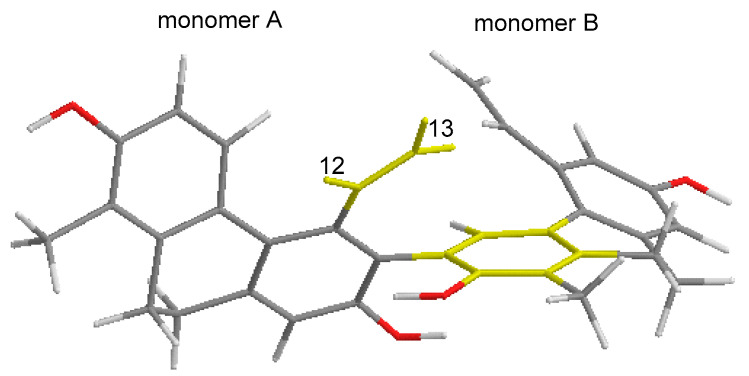
The calculated molecular structure of tenuin E (**5**). It is shown that the shielded allylic protons H-12 and H-13 of the A monomer are situated within the shielding cone of the aromatic ring of the B monomer.

The molecular formula for compound **5** (tenuin E) is C_34_H_30_O_4_ based on the HRESIMS analysis (*m*/*z* 501.2066 [M–H]^−^, calcd for C_34_H_29_O_4_^−^, 501.2071) (Figure S40). An analysis of the ^1^H NMR, ^13^C NMR JMOD, HSQC, and NOESY spectra (Figures S33–S35 and S38) for this compound revealed a dihydrophenanthrenoid dimer comprising two effusol (**10**) units (Table 2). In compound **5**, the methine groups at C-6 of unit A and C-3′ of unit B were replaced by quaternary carbons (*δ*_C_ 123.8 and 122.4). This hypothesis was confirmed further through an analysis of the ^1^H−^1^H COSY (Figure S36) and HMBC data (Figure 2 and Figure S37). The HMBC long-range correlation observed from H-4′ (*δ*_H_ 7.00 s) to C-6 (*δ*_C_ 123.8) proved the C-6–C-3′connection of the two monomers (Figure 2), establishing the structure of **5** as 1,1′-dimethyl-5,5′-divinyl-9,9′,10,10′-tetrahydro-[6,3′-biphenanthrene]-2,2′,7,7′-tetraol. The anisotropic effect of the aromatic ring of monomer B, which caused shielding of the H-12 and H-13 signals, can be observed (Figure 4).

**Figure 5:**
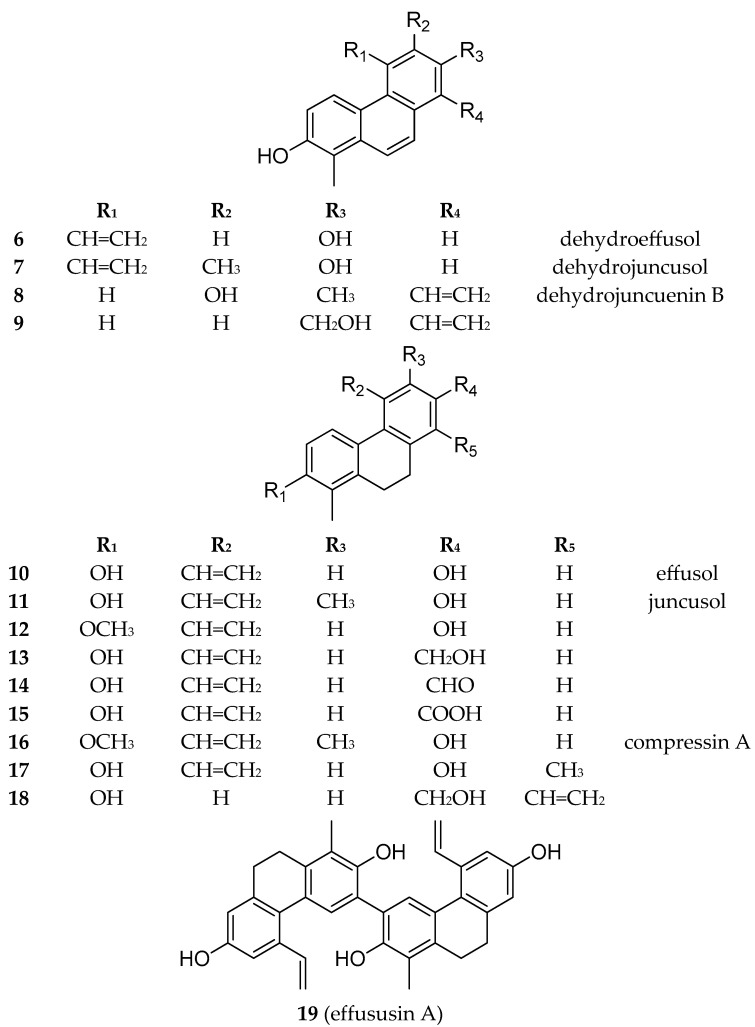
Structures of known phenanthrenes (**6**–**19**) isolated from *J. tenuis*.

Besides the new compounds tenuins A–E (**1**–**5**), 14 known phenanthrenes (**6**–**19**) (Figure 5), with 4 dehydrophenanthrenes among them, namely dehydroeffusol (**6**) (Figure S41) [21], dehydrojuncusol (**7**) (Figure S42) [22], dehydrojuncuenin B (**8**) (Figure S43) [20], and its hydroxyderivative (**9**) (Figure S44) [23]; 9 dihydrophenanthrenes (**10**–**18**) (Figures S45–S53) [24,25,26,27,28,29]; 1 dimer (effususin A, **19**) (Figure S54) [30]; luteolin; 5,7-dihydroxy-4-chromone; *p*-hydroxybenzoic [31] acid; vanillic [32] acid; and *trans-p*-coumaric acid were identified from *J. tenuis*. Structural characterization was carried out using HRESIMS, together with one- and two-dimensional NMR experiments. The ^1^H and ^13^C chemical shifts were assigned and compared with data from the literature for confirmation. Except for effusol (**10**), juncusol (**11**), and 2,7-dihydroxy-1,8-dimethyl-5-vinyl-9,10-dihydrophenanthrene (**17**), all of the compounds were isolated from this plant species for the first time.

**Figure 6:**
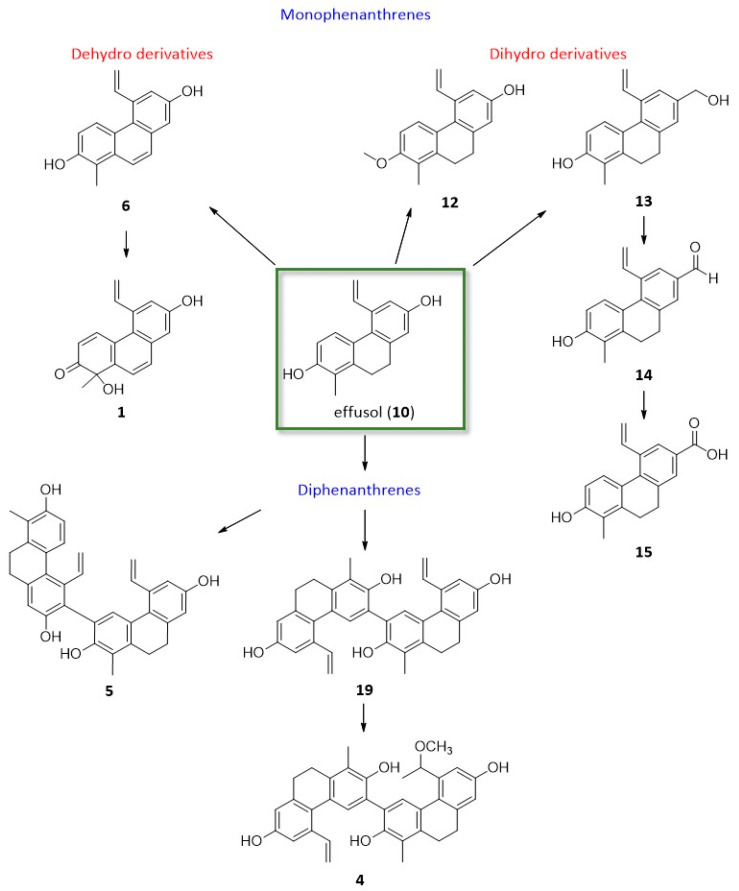
Effusol (**10**) derivatives from *J. tenuis*.

Effusol (**10**) and juncusol (**11**) are the main phenanthrenes from *J. tenuis*. Dehydro derivatives (**6** and **7**) of these compounds were also identified from the plant. Similarly, compounds **9** and **18** are also dehydro–dihydro pairs. Most of the phenanthrenes are dihydro derivatives (**3**–**5**, **10**–**19**). Among the phenanthrenes, mono- (**1**–**3**, **6**–**18**) and diphenanthrenes (**4**, **5**, **19**) also occur. All compounds are vinyl-substituted. The vinyl group is mainly joined at C-5; in the case of **2**, it was connected at C-6, while in **8**, **9**, and **18**, it was connected at C-8. Most probably, the 5-vinyl phenanthrenes could originate from effusol (**10**) and juncusol (**11**) (Figure 6). In compound **4**, one of the vinyl groups was modified into a methoxy ethylene group. Compounds **10** and **13**–**15** differ only in the substituent at C-7: the hydroxyl group in **10**, the hydroxymethyl group in **13**, the formyl group in **14**, and the carboxyl group in **15**. Based on our results, it can be stated that the phenanthrene content of this plant sample was very different from that investigated previously by our group.

### 2.2. The Antiproliferative Activity of the Compounds

The antiproliferative effects of the isolated phenanthrenes (compounds **1**–**19**) were evaluated using human cancer cell lines, including doxorubicin-sensitive colonic adenocarcinoma COLO 205 and multidrug-resistant colonic adenocarcinoma COLO 320/MDR-LRP (which expresses P-glycoprotein [MDR1] and LRP), as well as non-cancerous human lung fibroblast CCD-19Lu cells. CCD-19Lu was chosen because it is a well-characterized, non-tumorigenic cell line commonly used to assess general cytotoxicity. Moreover, data from the literature support the use of lung fibroblast cell lines—including CCD-19Lu—as normal controls in studies involving colon cancer cells such as Colo 205 and Colo 320 [33]. The thiazolyl blue tetrazolium bromide (MTT) assay was employed to determine the concentration of each compound required to inhibit 50% of the cell viability (IC_50_), as summarized in Table 3. Among the tested compounds, the dimeric phenanthrene tenuin D (compound **4**) exhibited the most potent antiproliferative activity against the COLO 205 cell line, with an IC_50_ value of 7.60 μM. The other two dimers (**5** and **19**) were also proven to be active, especially against the COLO 205 cell line (IC_50_ values of 11.6 μM for **5** and 10.9 μM for **19**, respectively). Effusol (**10**), the monomer of the dimers, was less active (IC_50_ values of 52.7 μM in COLO 205 and 49.7 μM in the COLO 320 cell line). Saturation of the double bond between C-9 and C-10 led to decreased activity in the case of dehydroeffusol (**6**) and effusol (**10**), whereas no significant difference was observed for the other pairs, **7** and **11**, and **9** and **18**.

The isolated phenanthrenes were slightly selective or non-selective towards the tumor cells. Compound **4** was the most selective towards one of the tumor cell lines (COLO 205, SI = 0.49), while compressin A (**16**) showed selectivity towards the multi-drug resistant COLO 320 cells (SI = 1.87). Considering the tumor selectivity, the highest selectivity index (SI CCD-19Lu/COLO 320) was observed for tenuin B (**2**, SI = 2.51) and compound **18** (SI = 2.25).

All of the tested compounds were evaluated in four parallel measurements. The selectivity index (SI) was calculated as the ratio of the IC_50_ value in the non-tumorigenic cells to the IC_50_ value in the cancer cell lines. An SI value greater than 6 indicates strong selectivity toward cancer cells, values between 3 and 6 denote moderate selectivity, values between 1 and 3 indicate slight selectivity, and an SI below 1 suggests a lack of selectivity.

### 2.3. The Drug Combination Assay

Many types of cancer exhibit significant resistance to the currently available chemotherapeutic agents, highlighting the need for novel, effective, and well-tolerated therapeutic strategies. One promising approach involves the discovery of new bioactive natural products. To explore this, a chemosensitivity assay was conducted to examine the in vitro interactions between the isolated compounds and the antineoplastic agent doxorubicin, a substrate of P-glycoprotein (P-gp). A combination chemotherapy model was applied using human COLO 320 colon carcinoma cells. The combination index (CI), calculated according to the Chou–Talalay method, was used to evaluate drug–drug interactions, classifying them as synergistic (CI < 1), additive (CI = 1), or antagonistic (CI > 1) (Table 4) [34]. As shown in Table 4, the majority of the tested compounds demonstrated synergistic interactions with doxorubicin (CI < 1) in the COLO 320 cell line. Tenuin B (compound **2**) exhibited a particularly strong synergistic effect, with combination index (CI) values below 0.1. This compound showed weak antiproliferative activity (IC_50_: 48.4 and 38.3 μM) (Table 3). Moreover, strong synergisms were detected for compounds **6**, **7**, **13**, **14**, and **18**.

In recent years, several phenanthrenes from the Juncaceae family have been tested for their in vitro cytotoxicity against various cancer cell lines using different test systems, exhibiting promising activities. Their in vitro effects may be mediated by several potential mechanisms, e.g., cell membrane rupture, impaired cell metabolism, DNA damage, or a combination of these [36]. The anticancer activity of phenanthrenes is strongly influenced by their structural features, including the oxidation state, degree of saturation, substitution pattern, and dimerization. In this study, newly isolated phenanthrenes from *J. tenuis*, particularly the diphenanthrenes tenuin D (**4**), tenuin E (**5**), and effususin A (**19**), showed the most potent in vitro antiproliferative activity against the COLO 205 colorectal cancer cell line, with IC_50_ values in the range of 7.6–11.6 μM. Their superior activity compared to that of their monomeric counterparts suggests that dimerization enhances their cytotoxic potential, potentially by increasing the molecular size, lipophilicity, or interaction with multiple cellular targets. Reports from the literature also support the enhanced bioactivity of phenanthrene dimers. For example, effususin B isolated from *J. effusus* showed strong cytotoxic activity against three cancer cell lines (SMMC-7721, HepG-2, and MCF-7), with IC_50_ values of 13.60, 12.93, and 12.49 µM, respectively [30].

Among the monomers, e.g., juncunol had an IC_50_ value of 18 µM in the HepG2 cells, and it induced an increase in the number of apoptotic cells in a concentration-dependent manner (IC_50_ value ± 25%) accompanied by a decrease in Δψm [37]. The compound induced cel cycle arrest in the G0/G1 phase, while showing no hemolytic properties. In silico studies indicate that that compound seems to bind between GC base pairs and thus may act as a DNA intercalator [38].

The presence and position of vinyl groups can affect the activity of Juncaceae phenanthrenes. Most of the phenanthrenes in this study carried a vinyl substituent, often at C-5. However, tenuin B (**2**), with a vinyl group at C-6, demonstrated a unique profile, including very strong synergism with doxorubicin (CI = 0.021), despite its relatively weak antiproliferative activity. This suggests that the side-chain positioning may modulate interactions with efflux transporters or drug-metabolizing enzymes, potentially influencing chemosensitization.

Further studies incorporating molecular docking, metabolic profiling, and target identification are needed to deepen our understanding of how these structural motifs govern anticancer efficacy and selectivity.

## 3. Materials and Methods

### 3.1. The General Procedures

Optical rotations were measured using a JASCO P-2000 polarimeter (JASCO, Tokyo, Japan), and the UV spectra were obtained using a Shimadzu UV-1800 spectrophotometer. Vacuum liquid chromatography (VLC) was performed on silica gel (silica gel GF254, 15 µm, Merck, Darmstadt, Germany). Sephadex LH-20 (25–100 µm, Sigma-Aldrich, Budapest, Hungary) was employed for gel filtration. HPLC was performed on a Shimadzu HPLC system using reversed-phase columns (Phenomenex Luna Phenyl-Hexyl, 5 µm, 100 A, 250 × 10 mm; Phenomenex Kinetex Phenyl-Hexyl, 5 µm, 100 A, 150 × 4.6 mm). For the analysis of the compounds with chiral carbon atoms, a Lux Amylose-1 column (5 µm, 250 × 4.6 mm, Phenomenex, Torrance, CA, USA) was utilized with cyclohexane–isopropanol 82:18 as the mobile phase. All of the solvents used for CC were of at least analytical grade (VWR Ltd., Szeged, Hungary).

The NMR spectra were recorded in methanol-*d*_4_ using a Bruker Avance DRX 500 spectrometer (Bruker, Germany) operating at 500 MHz for ^1^H and 125 MHz for ^13^C. The residual solvent signals of CD_3_OD (*δ*_H_ 4.78 and 3.31; *δ*_C_ 49.2) were used as internal references. The chemical shift values (*δ*) are reported in parts per million (ppm), and coupling constants (*J*) are given in hertz (Hz). Two-dimensional NMR experiments were performed using standard Bruker software (TopSpin 3.6.2), employing gradient-enhanced techniques for the COSY, HSQC, and HMBC spectra. High-resolution mass spectrometry (HRMS) data were acquired using a Thermo Scientific Q-Exactive Plus Orbitrap mass spectrometer equipped with an electrospray ionization (ESI) source, operating in both positive and negative ionization modes. The data acquisition and processing were carried out using MassLynx software (Version 4.1).

### 3.2. The Plant Material

Whole plants of *Juncus tenuis* Willd. were collected during the flowering period in June 2020 from a sandy, dried lakebed near Barcs, Hungary (GPS coordinates: 45°58′30.817″ N, 17°32′17.764″ E). The plant material was botanically identified by Dragica Purger (Department of Pharmacognosy, University of Pécs, Hungary). A voucher specimen (No. 901) was deposited into the herbarium of the Department of Pharmacognosy, the University of Szeged, Szeged, Hungary.

### 3.3. Extraction and Isolation

The air-dried whole plant material of *Juncus tenuis* (3.38 kg) was ground and extracted at room temperature through percolation with methanol (30 L). The resulting crude methanolic extract (0.5 kg) was concentrated under reduced pressure and stored at −20 °C until further processing. Then, it was subjected to solvent–solvent partitioning using *n*-hexane (8 × 500 mL), chloroform (CHCl_3_, 10 × 500 mL), and ethyl acetate (EtOAc, 9 × 500 mL). The concentrated chloroform-soluble fraction (40 g) was separated further through vacuum liquid chromatography (VLC) on silica gel, employing a gradient elution system of cyclohexane–EtOAc–MeOH (ranging from 98:2:0 to 50:50:0; 1500 mL per eluent), followed by MeOH. A total of 11 major fractions (designated as fractions 1–11) were collected in 100 mL portions. The fractionation was monitored through thin-layer chromatography (TLC), and fractions with similar profiles were combined based on their chromatographic patterns. Subsequently, all major fractions were separated further through gel chromatography on a Sephadex LH-20 stationary phase using CH_2_Cl_2_–MeOH (1:1) as the eluent.

Fraction 11/1 was pure and yielded luteolin (26.6 mg). Fraction 1/3 was purified through RP-HPLC using a MeOH–H_2_O gradient solvent system (from 78:22 to 88:12 in 9 min; flow rate: 1 mL/min) as the mobile phase, yielding compounds **12** (*t*_R_ = 5.50 min, 2.1 mg) and **16** (*t*_R_ = 6.53 min, 1.4 mg). Purification of fraction 2/2 was performed through RP-HPLC under gradient conditions using MeOH–H_2_O (from 78:22 to 88:12 in 9 min; flow rate: 1 mL/min) as the mobile phase, yielding compound **14** (*t*_R_ = 6.63 min, 1.0 mg). Fraction 4/2 was purified through RP-HPLC using MeOH–H_2_O (65:35) as the eluent at a flow rate of 1 mL/min, yielding compounds **10** (*t*_R_ = 5.46 min, 574.9 mg), **11** (*t*_R_ = 6.78 min, 374.7 mg), and **17** (*t*_R_ = 8.12 min, 48.3 mg). Fraction 5/3 was separated through gel chromatography on a Sephadex LH-20 stationary phase using CH_2_Cl_2_–MeOH (1:1) as the eluent. Subfraction 5/3/2 was separated further through RP-HPLC under gradient conditions using MeOH–H_2_O (from 8:2 to 1:0 in 9 min; flow rate: 3 mL/min) as the mobile phase to yield compound **7** (*t*_R_ = 8.32 min, 1.0 mg). Fraction 6/3 was purified through RP-HPLC using a gradient solvent system of MeOH–H_2_O (from 85:15 to 1:0 in 8 min; flow rate: 3 mL/min) as the mobile phase, yielding seven subfractions. Subfraction 6/3/2 was separated further through RP-HPLC using a gradient solvent system of MeOH–H_2_O (from 55:45 to 1:0 in 7 min; flow rate: 1 mL/min) as the mobile phase, yielding compound **1** (*t*_R_ = 5.18 min, 2.4 mg). Purification of subfraction 6/3/4 was performed through RP-HPLC using MeOH–H_2_O (65:35) as the eluent at a flow rate of 1 mL/min, yielding compounds **13** (*t*_R_ = 6.30 min, 20.4 mg) and **18** (*t*_R_ = 6.79 min, 2.2 mg). Purification of fraction 7/2 was performed through RP-HPLC under gradient conditions using MeOH–H_2_O (from 7:3 to 1:0 in 11 min; flow rate: 3 mL/min) as the mobile phase, yielding six subfractions. Subfraction 7/2/1 was purified further through RP-HPLC using a gradient solvent system of MeOH–H_2_O (from 45:55 to 1:0 in 9 min; flow rate: 1 mL/min), yielding 5,7-dihydroxy-4-chromone (*t*_R_ = 5.00 min, 1.5 mg,). Subfraction 7/2/2 was separated through RP-HPLC using a gradient solvent system of MeOH–H_2_O (from 62:38 to 77:23 in 9 min; flow rate: 1 mL/min) to yield compounds **2** (*t*_R_ = 5.91 min, 1.5 mg) and **15** (*t*_R_ = 7.78 min, 3.1 mg). Purification of subfraction 7/2/3 was performed through RP-HPLC using a gradient solvent system of MeOH–H_2_O (from 7:3 to 85:15 in 10 min; flow rate: 1 mL/min) as the mobile phase, yielding compound **5** (*t*_R_ = 7.28 min, 1.9 mg). Subfraction 7/2/4 was separated through RP-HPLC under gradient conditions using MeOH–H_2_O (from 73:27 to 8:2 in 10 min; flow rate: 1 mL/min) as the mobile phase, yielding four subfractions. Subfraction 7/2/4/1 was pure and yielded compound **8** (*t*_R_ = 5.96 min, 0.9 mg). Subfraction 7/2/4/3 was purified further through RP-HPLC using a gradient solvent system of MeOH–H_2_O (from 1:1 to 8:2 in 8 min; flow rate 1 mL/min) as the mobile phase, yielding compound **19** (*t*_R_ = 6.30 min, 0.8 mg).

Fraction 8/3 was separated using gel chromatography with a Sephadex LH-20 stationary phase using CH_2_Cl_2_–MeOH (1:1) as the eluent. Subfraction 8/3/2 was purified further through RP-HPLC using a gradient solvent system of MeOH–H_2_O (from 85:15 to 1:0 in 9 min; flow rate: 3 mL/min) as the mobile phase, yielding six subfractions. Further purification of fraction 8/3/2/1 was performed through RP-HPLC under gradient conditions using MeOH–H_2_O (from 27:73 to 1:1 in 10 min; flow rate: 1 mL/min) as the mobile phase, yielding *p*-hydroxybenzoic acid (*t*_R_ = 4.93 min, 1.6 mg), vanillic acid (*t*_R_ = 5.46 min, 7.0 mg), and *trans-p*-coumaric acid (*t*_R_ = 7.20 min, 3.2 mg). Subfraction 8/3/2/2 was separated further through RP-HPLC under gradient conditions using MeOH–H_2_O (from 25:75 to 52:48 in 8.5 min; flow rate: 1 mL/min) as the mobile phase to yield compound **3** (*t*_R_ = 7.79 min, 1.2 mg). Subfraction 8/3/2/3 was purified further through RP-HPLC using a gradient solvent system of MeOH–H_2_O (from 35:65 to 85:15 in 8 min; flow rate: 1 mL/min) as the mobile phase, yielding compound **9** (*t*_R_ = 6.65 min, 1.6 mg). Further purification of fraction 8/3/2/6 was performed through RP-HPLC using MeOH–H_2_O (73:27) as the eluent at a flow rate of 1 mL/min, yielding compound **4** (*t*_R_ = 7.24 min, 1.5 mg). Purification of fraction 9/2 was performed through RP-HPLC using a gradient solvent system of MeOH–H_2_O (from 55:45 to 90:10 in 8 min; flow rate: 1 mL/min) as the mobile phase, yielding six subfractions. Subfraction 9/2/5 was purified further through RP-HPLC using a gradient solvent system of MeOH–H_2_O (from 63:37 to 8:2 in 7 min; flow rate: 1 mL/min) as the mobile phase, yielding compound **6** (*t*_R_ = 5.76 min, 1.0 mg).

### 3.4. Physical Characteristics of New Compounds

Tenuin A (**1**). Yellow amorphous solid; [α]_D_^26^ 0 (c 0.1, MeOH); UV (MeOH) λ_max_ (log ε) = 222 (4.09), 278 (3.85), 356 (3.12) nm (Figure S7); ^1^H and ^13^C NMR data: see Table 1; (–)-HRESIMS *m*/*z* 265.0871 [M–H]^−^ (calcd for C_17_H_14_O_3_, 265.0870).

Tenuin B (**2**). Light yellow amorphous solid; UV (MeOH) λ_max_ (log ε) = 215 (4.52), 269 (4.43) nm (Figure S15); ^1^H and ^13^C NMR data: see Table 1; (–)-HRESIMS *m*/*z* 263.1078 [M–H]^−^ (calcd for C_18_H_16_O_2_, 263.1067).

Tenuin C (**3**). Light brown amorphous solid; UV (MeOH) λ_max_ (log ε) = 210 (3.92), 266 (3.51) nm (Figure S23); ^1^H and ^13^C NMR data: see Table 1; (–)-HRESIMS *m*/*z* 281.1184 [M–H]^−^ (calcd for C_18_H_18_O_3_, 281.1183).

Tenuin D (**4**). Light yellow amorphous solid; [α]_D_^26^ 0 (c 0.1, MeOH); UV (MeOH) λ_max_ (log ε) = 212 (3.96), 244 (3.64), 272 (3.54) nm (Figure S31); ^1^H and ^13^C NMR data: see Table 2; (–)-HRESIMS *m*/*z* 533.2330 [M–H]^−^ (calcd for C_35_H_34_O_5_, 533.2334).

Tenuin E (**5**). Yellow amorphous solid; UV (MeOH) λ_max_ (log ε) = 215 (4.49), 283 (3.84) nm (Figure S39); ^1^H and ^13^C NMR data: see Table 2; (–)-HRESIMS *m*/*z* 501.2066 [M–H]^−^ (calcd for C_34_H_30_O_4_, 501.2071).

### 3.5. The Antiproliferative Assays

#### 3.5.1. Cell Lines

The human colon adenocarcinoma cells—COLO 205 (CCL-222, doxorubicin-sensitive) and COLO 320/MDR-LRP (ATCC-CCL-220.1, multidrug-resistant, expressing P-gp)—were cultured in RPMI 1640 medium supplemented with 10% heat-inactivated fetal bovine serum, 2 mM of L-glutamine, 1 mM of sodium pyruvate, and 100 mM of HEPES. The non-cancerous human lung fibroblast cell line CCD-19Lu was maintained in Eagle’s Minimal Essential Medium (EMEM; containing 4.5 g/L of glucose) supplemented with a non-essential amino acid mixture, selected vitamins, and 10% heat-inactivated fetal bovine serum. All of the cell lines were detached using 0.25% trypsin and 0.02% EDTA for 5 min at 37 °C. The cell lines were obtained from LGC Promochem (Teddington, England).

#### 3.5.2. The Antiproliferative Assay

Human colonic adenocarcinoma cell lines—doxorubicin-sensitive COLO 205 and multidrug-resistant COLO 320—as well as the non-cancerous human lung fibroblast cell line CCD-19Lu were used to evaluate the effects of the test compounds on cell proliferation. The assays were conducted in 96-well flat-bottomed microtiter plates. Stock solutions of the compounds were prepared in dimethyl sulfoxide (DMSO), with the final DMSO concentration kept below 1% in all samples. The compounds were diluted in 100 µL of the appropriate culture medium. Adherent cells were cultured in EMEM supplemented with 10% heat-inactivated fetal bovine serum and seeded into 96-well plates at a density of 6 × 10^3^ cells per 100 µL per well. The cells were allowed to adhere for 24 h at 37 °C in a humidified incubator with 5% CO_2_. After incubation, the medium was removed and replaced with 100 µL of fresh medium. Serial dilutions of the compounds were prepared in separate plates and added to the cells, starting from a concentration of 100 µM with twofold serial dilutions (final concentration range: 100–0.19 µM). The cells were then incubated for 72 h at 37 °C. At the end of the incubation period, 20 µL of thiazolyl blue tetrazolium bromide (MTT) solution (5 mg/mL, Sigma) was added to each well. After 4 h of incubation at 37 °C, 100 µL of 10% sodium dodecyl sulfate (SDS) in 0.01 M HCl was added to solubilize the formazan crystals, and the plates were incubated overnight at 37 °C. Cell viability was assessed by measuring the optical density (OD) at 540/630 nm using a Multiscan EX ELISA reader (Thermo Labsystems, Cheshire, WA, USA).

The inhibitory concentration that reduced cell growth by 50% (IC_50_) was determined from the dose–response curves. The percent inhibition was calculated using the formula

100 − ((OD_sample − OD_medium control)/(OD_cell control − OD_medium control)) × 100

Dose–response curves were generated by plotting the percent inhibition against the logarithm of the compound concentrations and fitted using Prism5 software (Version 6, GraphPad Software Inc., San Diego, CA, USA). The IC_50_ values were derived from four independent experiments conducted in parallel for each cell line and are reported as the mean values [38].

All reagents and cell culture media used for cell maintenance were obtained from Merck (Darmstadt, Germany).

#### 3.5.3. The Drug Combination Assay

The multidrug-resistant COLO 320 cell line was used to perform the drug combination assay. Doxorubicin (2 mg/mL, Teva Pharmaceuticals, Budapest, Hungary) was serially diluted horizontally, starting with 8.6 µM. The resistance modifier was subsequently diluted vertically, with the starting concentration determined based on the IC_50_ value. Doxorubicin was diluted horizontally in 100 µL, while the resistance modifiers were diluted vertically in 50 µL in the microtiter plate. The compounds and doxorubicin were prepared as separate dilutions. The cells were seeded at a density of 6 × 10^3^ cells per well into 100 µL of medium and incubated for 24 h at 37 °C in a 5% CO_2_ atmosphere prior to treatment. After this period, the culture medium was removed, and 50 µL of fresh medium was added to each well, followed by the addition of 50 µL of the diluted compounds, resulting in a final volume of 200 µL per well. The plates were then incubated for 72 h at 37 °C under 5% CO_2_. The cell viability was assessed at the end of incubation using the MTT assay, as described previously. Drug interactions were analyzed using CompuSyn software (Version 1.00) [39].

The dose–response curves for individual agents and their combinations were fitted to a linear model based on the median-effect equation to calculate the median effective dose (IC_50_) and the slope (m) [35,40]. The quality of the fit was evaluated using the linear correlation coefficient (r), and only data with r > 0.90 are reported. The extent of drug interaction was quantified using the combination index (CI), where CI ≈ 1 indicated an additive effect, CI < 1 signified synergy, and CI > 1 denoted antagonism.

## 4. Conclusions

In this study, 19 phenanthrenes—including tenuins A–E (**1**–**5**) as new natural products and 14 known compounds (**6**–**19**)—along with luteolin, 5,7-dihydroxy-4-chromone, *p*-hydroxybenzoic acid, vanillic acid, and *trans-p*-coumaric acid were characterized from the whole plant of *J. tenuis*. Comprehensive spectroscopic data elucidated the planar structures of these compounds. Except for effusol (**10**), juncusol (**11**), and 2,7-dihydroxy-1,8-dimethyl-5-vinyl-9,10-dihydrophenanthrene (**17**), all of the compounds were reported from this plant for the first time. The phenanthrene dimers **4**, **5**, and **19** exhibited in vitro antiproliferative activity against the COLO 205 tumor cell line, with tenuin D (**4**) identified as the most promising compound, with a strong effect (IC_50_ = 7.6 μM). In addition, tenuin B (**2**) exhibited very strong synergism with doxorubicin in the drug combination assay.

These findings expand the phytochemical and pharmacological knowledge of *J. tenuis* and support the Juncaceae family as a valuable source of bioactive phenanthrenes. Future research should focus on further mechanistic studies to understand the molecular basis of the synergistic effects observed with doxorubicin, particularly in multidrug-resistant cancer models. Additionally, structure–activity relationship (SAR) studies and semi-synthetic modifications of the newly identified phenanthrenes may help improve their tumor selectivity and pharmacokinetic profiles. In vivo evaluations and formulation approaches such as nanoencapsulation or targeted delivery systems could enhance their therapeutic potential while minimizing their toxicity. Continued exploration of other *Juncus* species and related genera may also uncover novel phenanthrene scaffolds with promising biological properties.

## Figures and Tables

**Table 1:** ^1^H (500 MHz) and ^13^C NMR (125 MHz) data for compounds **1**–**3** in methanol-*d*_4_.

Position	1		2		3	
	*δ*_H_ (*J* in Hz)	*δ*_C_, Type	*δ*_H_ (*J* in Hz)	*δ*_C_, Type	*δ*_H_ (*J* in Hz)	*δ*_C_, Type
1		78.3, C		118.9, C		121.7, C
1a		146.7, C		133.9, C		140.0, C
2		206.9, C		154.7, C		155.1, C
3	6.13, d (10.4)	121.6, CH	7.22, d (8.9)	117.5, CH	6.63, d (8.4)	112.3, CH
4	8.73, d (10.4)	145.4, CH	8.50, d (8.9)	122.4, CH	7.13, d (8.4)	128.6, CH
4a		125.7, C		125.2, C		127.3, C
5a		125.4, C		131.3, C		136.7, C
5		139.5, C	8.75, s	120.3, CH		128.3, C
6	7.18, s	122.5, CH		136.7,* C	6.92, s	113.1, CH
7		156.2, C		136.8,* C		155.3, C
8	7.09, s	111.3, CH	7.84, s	128.7, CH		124.4, C
8a		137.3, C		131.8, C		141.1, C
9	7.79, s	131.6, CH	7.72, d (9.2)	127.6, CH	2.76, m	26.9, CH_2_
10	7.79, s	124.7, CH	7.90, d (9.2)	123.8, CH	2.69, m	26.5, CH_2_
11	1.55, s	32.1, CH_3_	2.55, s	11.0, CH_3_	2.22, s	11.7, CH_3_
12	7.35, dd (17.3, 10.7)	141.8, CH	7.27, dd (17.4, 11.0)	136.1, CH	6.90, dd (17.4, 11.0)	140.7, CH
13	5.69, d (17.3) 5.44, d (10.8)	115.7, CH_2_	5.93, dd (17.4, 1.2) 5.43, dd (11.0, 1.2)	116.6, CH_2_	5.64, dd (17.4, 1.4) 5.18, dd (11.0, 1.3)	113.2, CH_2_
14			4.86, s	63.4, CH_2_	4.79, s	56.7, CH_2_

* Interchangeable signals. d doublet, m multiplet, s singulet.

**Table 2:** ^1^H (500 MHz) and ^13^C NMR (125 MHz) data for compounds **4** and **5** in methanol-*d*_4_.

Position	4		5	
	δH (J in Hz)	δC, Type	δH (J in Hz)	δC, Type
1		124.1,^a^ C		121.6, C
1a		140.0, C		140.4, C
2		151.6,^b^ C		154.8, C
3		125.2, C	6.58, d (8.5)	112.2, CH
4	7.04, s	129.9, CH	7.44, d (8.5)	129.4, CH
4a		128.4, C		127.3, C
5a		128.7, C		128.5, C
5		142.4, C		137.9, C
6	6.87, d (2.8)	112.6, CH		123.8, C
7		152.7, C		154.6, C
8	6.67, d (2.8)	114.7, CH	6.80, s	114.7, CH
8a		141.9,^c^ C		141.8, C
9	2.72, m	32.0, CH_2_	2.69, m	31.8, CH_2_
10	2.54, m, 2.94, m	27.0, CH_2_	2.69, m	26.8, CH_2_
11	2.35, s	12.6,^d^ CH_3_	2.22, s	11.8, CH_3_
12	4.98, q (6.2)	76.5, CH	6.63, dd (17.9, 11.8)	138.5, CH
13	1.47 d (6.2)	23.6, CH_3_	5.03, dd (11.8, 1.9) 4.85, dd (18.0, 1.9)	119.2, CH_2_
12-OCH_3_	2.93, s	55.9, CH_3_		
1′		124.1,^a^ C		122.6, C
1a′		139.5, C		139.2, C
2′		151.6,^b^ C		152.2, C
3′		125.3, C		122.4, C
4′	7.41, s	130.8, CH	7.00, s	131.3, CH
4a′		128.1, C		127.8, C
5a′		127.3, C		127.7, C
5′		137.7, C		137.5, C
6′	6.86, d (2.7)	113.9, CH	6.82, d (2.5)	113.5, CH
7′		156.7, C		156.4, C
8′	6.68, d (2.7)	115.1, CH	6.65, d (2.5)	115.0, CH
8a′		141.9,^c^ C		141.4, C
9′	2.69, m	31.5, CH_2_	2.69, m	31.6, CH_2_
10′	2.84, m, 2.96, m	26.8, CH_2_	2.80, m	26.7, CH_2_
11′	2.35, s	12.5,^d^ CH_3_	2.28, s	12.3, CH_3_
12′	7.03, dd (18.4, 10.6)	140.4, CH	7.00, dd (17.7, 10.6)	140.5, CH
13′	5.63, dd (17.4, 1.1) 5.15, dd (10.9, 1.0)	114.0, CH_2_	5.54, dd (17.4, 0.9) 5.07, dd (10.7, 1.5)	113.4, CH_2_

^a,b,c,d^ Overlapping signals. m multiplet, s singulet, q quartet

**Table 3:** The antiproliferative activity (IC_50_ values) of the isolated compounds (**1**–**19**) (SI is the selectivity index).

Compound	IC_50_ (µM) ± SD			SI COLO 205/ COLO 320	SI CCD-19Lu/ COLO 205	SI CCD-19Lu/ COLO 320
	COLO 205	COLO 320	CCD-19Lu			
**1**	>100	>100	>100			
**2**	48.35 ± 1.03	38.34 ± 1.84	96.08 ± 0.82	1.26	1.99	2.51
**3**	>100	>100	96.80 ± 1.79		≤0.97	≤0.97
**4**	7.60 ± 0.13	15.65 ± 2.20	9.93 ± 0.84	0.49	1.31	0.63
**5**	11.59 ± 0.22	11.97 ± 1.58	13.79 ± 0.82	0.97	1.19	1.15
**6**	36.74 ± 2.29	35.65 ± 3.89	58.17 ± 0.51	1.03	1.58	1.63
**7**	28.16 ± 0.27	45.33 ± 2.78	42.04 ± 0.39	0.62	1.49	0.93
**8**	20.74 ± 0.47	17.93 ± 1.59	22.78 ± 0.55	1.16	1.10	1.27
**9**	>100	42.55 ± 2.98	75.27 ± 2.59	≥2.35	≤0.75	1.77
**10**	52.74 ± 1.85	49.69 ± 2.12	96.01 ± 2.66	1.06	1.82	1.93
**11**	31.93 ± 1.91	36.17 ± 3.81	44.43 ± 2.82	0.88	1.39	1.23
**12**	34.73 ± 0.10	25.46 ± 2.05	28.66 ± 1.11	1.36	0.83	1.13
**13**	37.04 ± 3.28	35.24 ± 4.68	69.34 ± 1.77	1.05	1.87	1.97
**14**	57.20 ± 3.98	39.58 ± 0.27	66.44 ± 1.58	1.45	1.16	1.68
**15**	45.43 ± 2.16	47.58 ± 1.01	>100	0.95	≥2.20	≥2.10
**16**	35.83 ± 2.70	19.16 ± 0.32	28.04 ± 2.73	1.87	0.78	1.46
**17**	43.18 ± 3.82	35.05 ± 1.10	64.70 ± 3.36	1.23	1.50	1.85
**18**	50.16 ± 0.74	31.47 ± 2.65	70.77 ± 3.25	1.59	1.41	2.25
**19**	10.93 ± 0.51	17.32 ± 0.87	18.80 ± 0.06	0.63	1.72	1.09
doxorubicin	0.53 ± 0.05	1.72 ± 0.50	1.03 ± 0.18			
DMSO	>1	>1	>1			

**Table 4:** Interaction between doxorubicin and phenanthrenes (**2**, **4**–**16**, **18**, **19**) in multidrug-resistant COLO 320 cells.

Compound	CI at ED50	SD	Ratio	Interaction
**2**	0.021	0.009	11.6:1	very strong synergism
**4**	0.669	0.132	23.2:1	synergism
**5**	0.433	0.107	11.6:1	synergism
**6**	0.266	0.074	11.6:1	strong synergism
**7**	0.117	0.043	34.9:1	strong synergism
**8**	2.126	0.521	371.2:1	antagonism
**9**	1.427	0.251	558.4:1	moderate antagonism
**10**	1.123	0.204	558.4:1	slight antagonism
**11**	0.582	0.102	11.6:1	synergism
**12**	1.347	0.235	186.4:1	moderate antagonism
**13**	0.163	0.033	11.6:1	strong synergism
**14**	0.219	0.099	11.6:1	strong synergism
**15**	1.927	0.171	34.9:1	antagonism
**16**	2.312	0.301	185.6:1	antagonism
**17**	0.670	0.149	69.6:1	synergism
**18**	0.189	0.098	11.6:1	strong synergism
**19**	1.125	0.029	371.2:1	moderate antagonism

The combination index (CI) values are presented as the mean ± standard deviation (SD), calculated from different drug ratios at the median effective dose (ED_50_). The interpretation of the CI values is as follows: CI < 0.1 indicates very strong synergism; 0.1 < CI < 0.3, strong synergism; 0.3 < CI < 0.7, synergism; 0.7 < CI < 0.9, moderate to slight synergism; 0.9 < CI < 1.1, a nearly additive effect; 1.1 < CI < 1.45, slight to moderate antagonism; and 1.45 < CI < 3.3, antagonism [35].

## Data Availability

The original contributions presented in this study are included in the article/Supplementary Material. Further inquiries can be directed to the corresponding author.

## Supplementary Materials

The following supporting information can be downloaded at https://www.mdpi.com/article/10.3390/ijms26167665/s1.
