# Supplementary material for: Phenanthrene Monomers and Dimers from Juncus tenuis with Antiproliferative Activity and Synergistic Effect with Doxorubicin Against Human Colon Cancer Cell Lines
Source: Int J Mol Sci. 2025 Aug 8;26(16):7665. doi: 10.3390/ijms26167665 (PMC12386809; doi:10.3390/ijms26167665)
Supplement: Supplementary file 1 [file ijms-26-07665-s001.zip › ijms-3755125-supplementary.pdf]

SUPPLEMENTARY MATERIALS

Phenanthrene Monomers and Dimers from Juncus tenuis with Antiproliferative Activity and Synergistic Effect with Doxorubicin Against Human Colon Cancer Cell Lines

TABLE OF CONTENTS

Figure S1. ^{1}H NMR spectrum of compound **1** (500 MHz, in CD_3OD). 4
Figure S2. ^{13}C (JMOD) NMR spectrum of compound **1** (125 MHz, in CD_3OD). 4
Figure S3. HSQC spectrum of compound **1** (in CD_3OD). 5
Figure S4. ^{1}H-^{1}H COSY spectrum of compound **1** (in CD_3OD). 5
Figure S6. NOESY spectrum of compound **1** (in CD_3OD). 6
Figure S7. UV spectrum of compound **1** in MeOH. 7
Figure S8. (-)-HRESIMS spectrum of compound **1**. 7
Figure S9. ^{1}H NMR spectrum of compound **2** (500 MHz, in CD_3OD). 8
Figure S10. ^{13}C (JMOD) NMR spectrum of compound **2** (125 MHz, in CD_3OD). 8
Figure S11. HSQC spectrum of compound **2** (in CD_3OD). 9
Figure S12. ^{1}H-^{1}H COSY spectrum of compound **2** (in CD_3OD). 9
Figure S13. HMBC spectrum of compound **2** (in CD_3OD). 10
Figure S15. UV spectrum of compound **2** in MeOH. 11
Figure S16. (-)-HRESIMS spectrum of compound **2**. 11
Figure S17. ^{1}H NMR spectrum of compound **3** (500 MHz, in CD_3OD). 12
Figure S18. ^{13}C (JMOD) NMR spectrum of compound **3** (125 MHz, in CD_3OD). 12
Figure S19. HSQC spectrum of compound **3** (in CD_3OD). 13
Figure S20. ^{1}H-^{1}H COSY spectrum of compound **3** (in CD_3OD). 13
Figure S21. HMBC spectrum of compound **3** (in CD_3OD). 14
Figure S22. NOESY spectrum of compound **3** (in CD_3OD). 14
Figure S23. UV spectrum of compound **3** in MeOH. 15
Figure S24. (-)-HRESIMS spectrum of compound **3**. 15
Figure S25. ^{1}H NMR spectrum of compound **4** (500 MHz, in CD_3OD). 16
Figure S26. ^{13}C (JMOD) NMR spectrum of compound **4** (125 MHz, in CD_3OD). 16
Figure S27. HSQC spectrum of compound **4** (in CD_3OD). 17
Figure S28. ^{1}H-^{1}H COSY spectrum of compound **4** (in CD_3OD). 17
Figure S29. HMBC spectrum of compound **4** (in CD_3OD). 18
Figure S30. NOESY spectrum of compound **4** (in CD_3OD). 18
Figure S31. UV spectrum of compound **4** in MeOH. 19
Figure S32. (-)-HRESIMS spectrum of compound **4**. 19
Figure S33. ^{1}H NMR spectrum of compound **5** (500 MHz, in CD_3OD). 20

Figure S34. ^{13}C (JMOD) NMR spectrum of compound **5** (125 MHz, in CD_3OD). 20
Figure S35. HSQC spectrum of compound **5** (in CD_3OD).. 21
Figure S36. ^1H-^1H COSY spectrum of compound **5** (in CD_3OD). 21
Figure S37. HMBC spectrum of compound **5** (in CD_3OD).. 22
Figure S38. NOESY spectrum of compound **5** (in CD_3OD). 22
Figure S39. UV spectrum of compound **5** in MeOH. 23
Figure S40. (-)-HRESIMS spectrum of compound **5**. 23
Figure S41. ^1H NMR spectrum of compound **6** (500 MHz, in CD_3OD).................................... 24
Figure S42. ^1H NMR spectrum of compound **7** (500 MHz, in CD_3OD).................................... 24
Figure S43. ^1H NMR spectrum of compound **8** (500 MHz, in CD_3OD).................................... 25
Figure S44. ^1H NMR spectrum of compound **9** (500 MHz, in CD_3OD).................................... 25
Figure S45. ^1H NMR spectrum of compound **10** (500 MHz, in CD_3OD).................................. 26
Figure S46. ^1H NMR spectrum of compound **11** (500 MHz, in CD_3OD).................................. 26
Figure S47. ^1H NMR spectrum of compound **12** (500 MHz, in CD_3OD).................................. 27
Figure S48. ^1H NMR spectrum of compound **13** (500 MHz, in CD_3OD).................................. 27
Figure S49. ^1H NMR spectrum of compound **14** (500 MHz, in CD_3OD).................................. 28
Figure S50. ^1H NMR spectrum of compound **15** (500 MHz, in CD_3OD).................................. 28
Figure S51. ^1H NMR spectrum of compound **16** (500 MHz, in CD_3OD).................................. 29
Figure S52. ^1H NMR spectrum of compound **17** (500 MHz, in CD_3OD).................................. 29
Figure S53. ^1H NMR spectrum of compound **18** (500 MHz, in CD_3OD).................................. 30
Figure S54. ^1H NMR spectrum of compound **19** (500 MHz, in CD_3OD).................................. 30

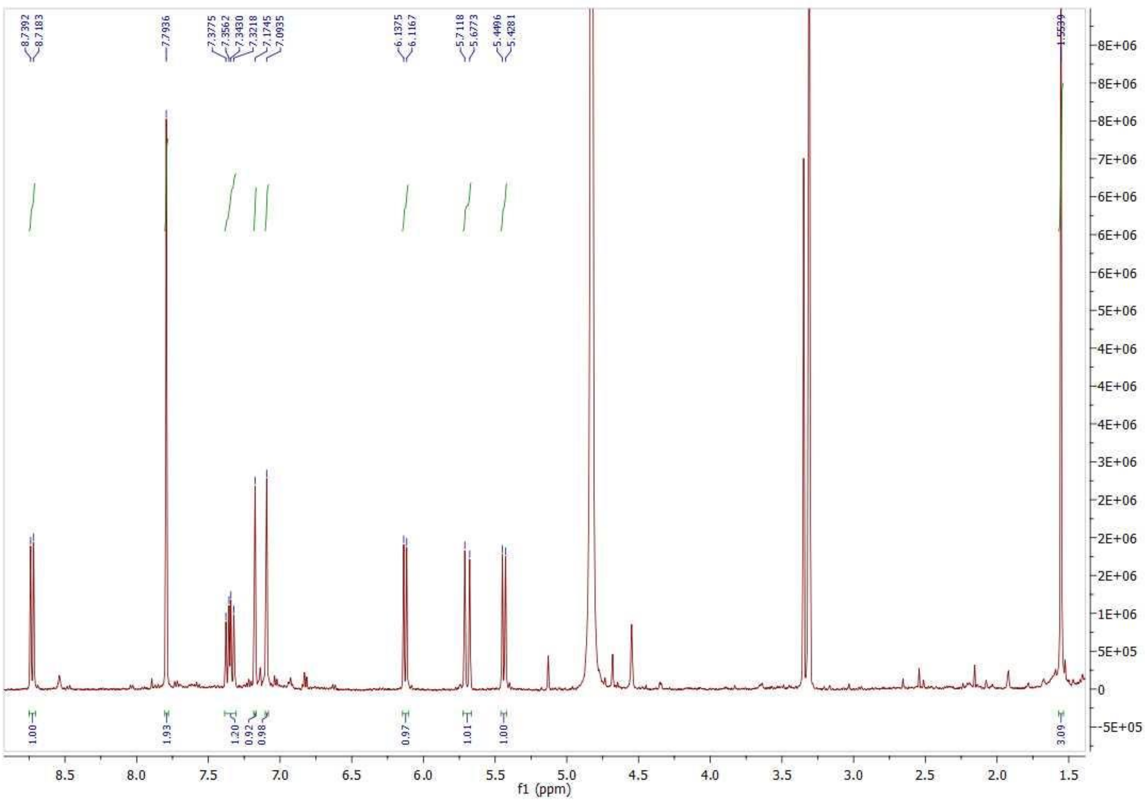

Figure S1. ^{1}H NMR spectrum of compound **1** (500 MHz, in CD_3OD).

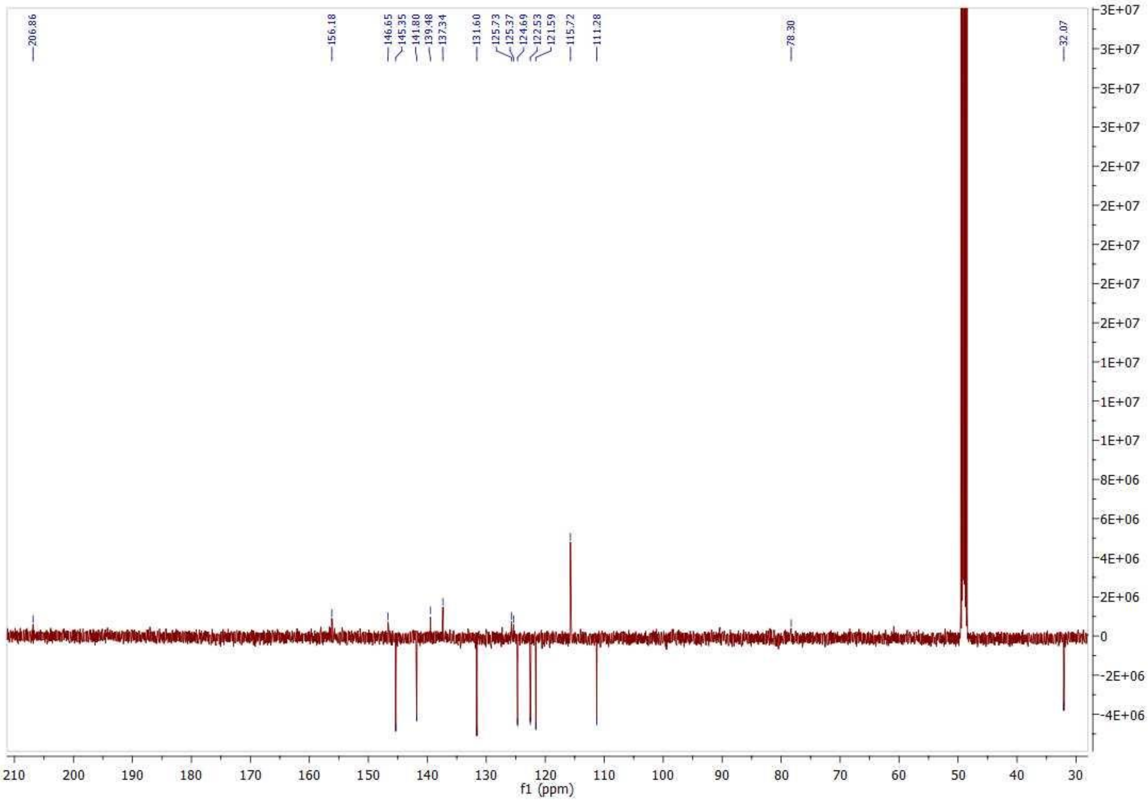

Figure S2. ^{13}C (JMOD) NMR spectrum of compound **1** (125 MHz, in CD_3OD).

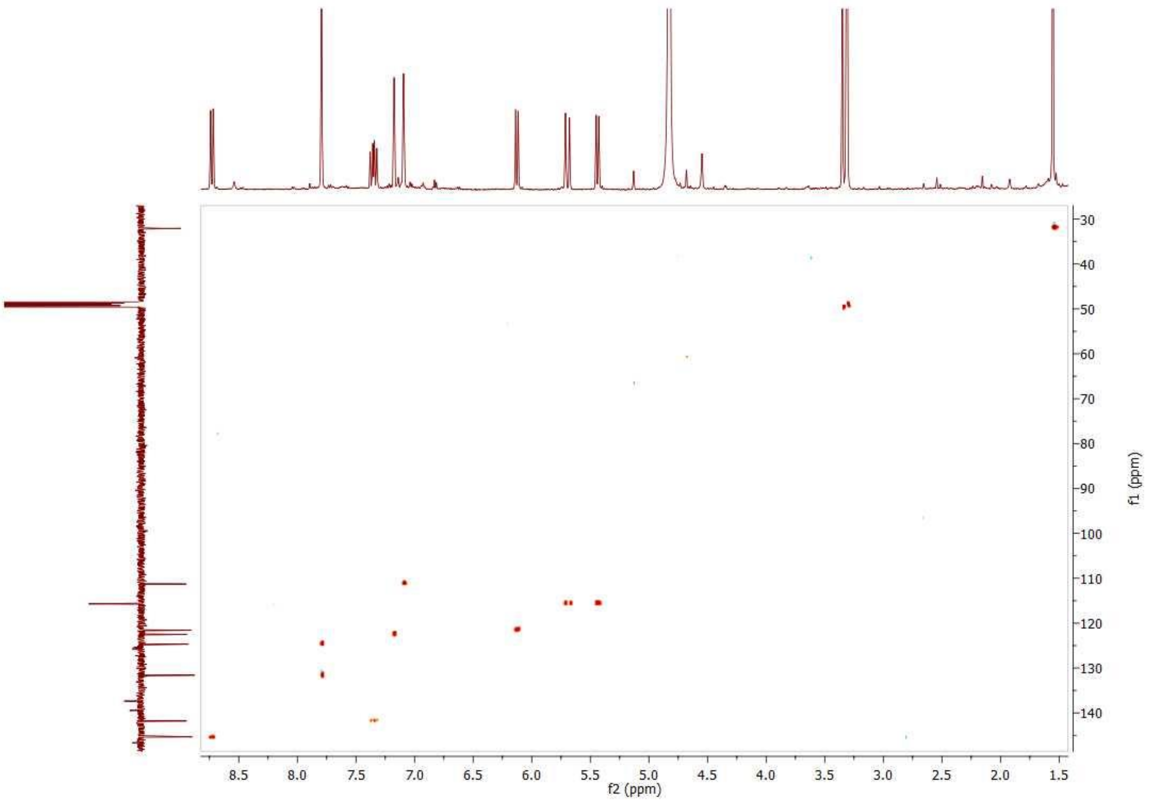

Figure S3. HSQC spectrum of compound **1** (in CD_3OD).

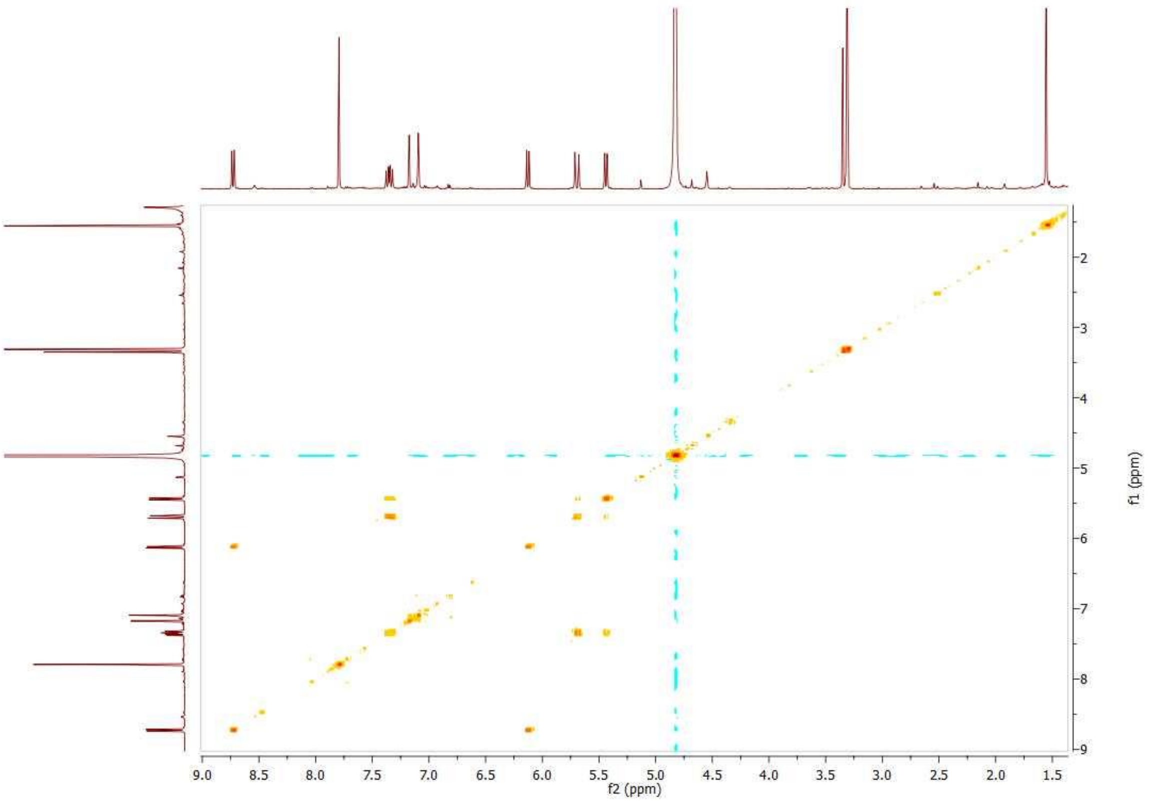

Figure S4. ^1H-^1H COSY spectrum of compound **1** (in CD_3OD).

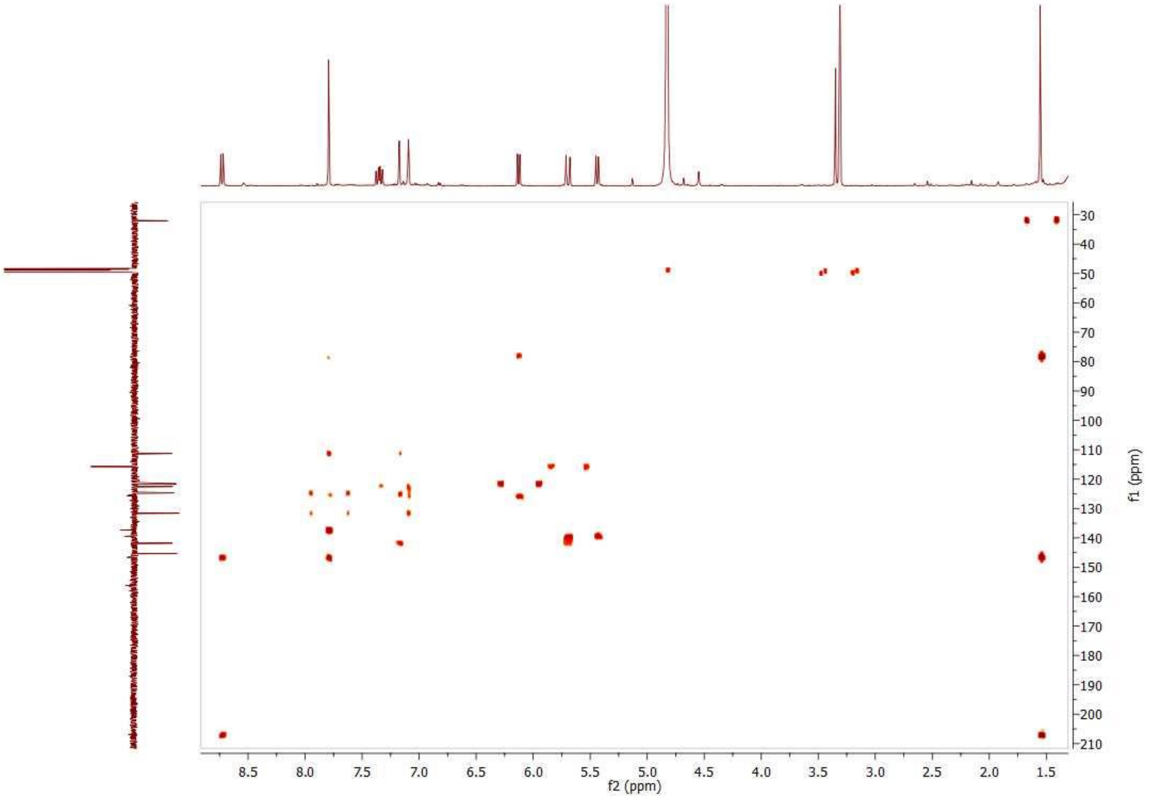

Figure S5. HMBC spectrum of compound **1** (in CD3OD).

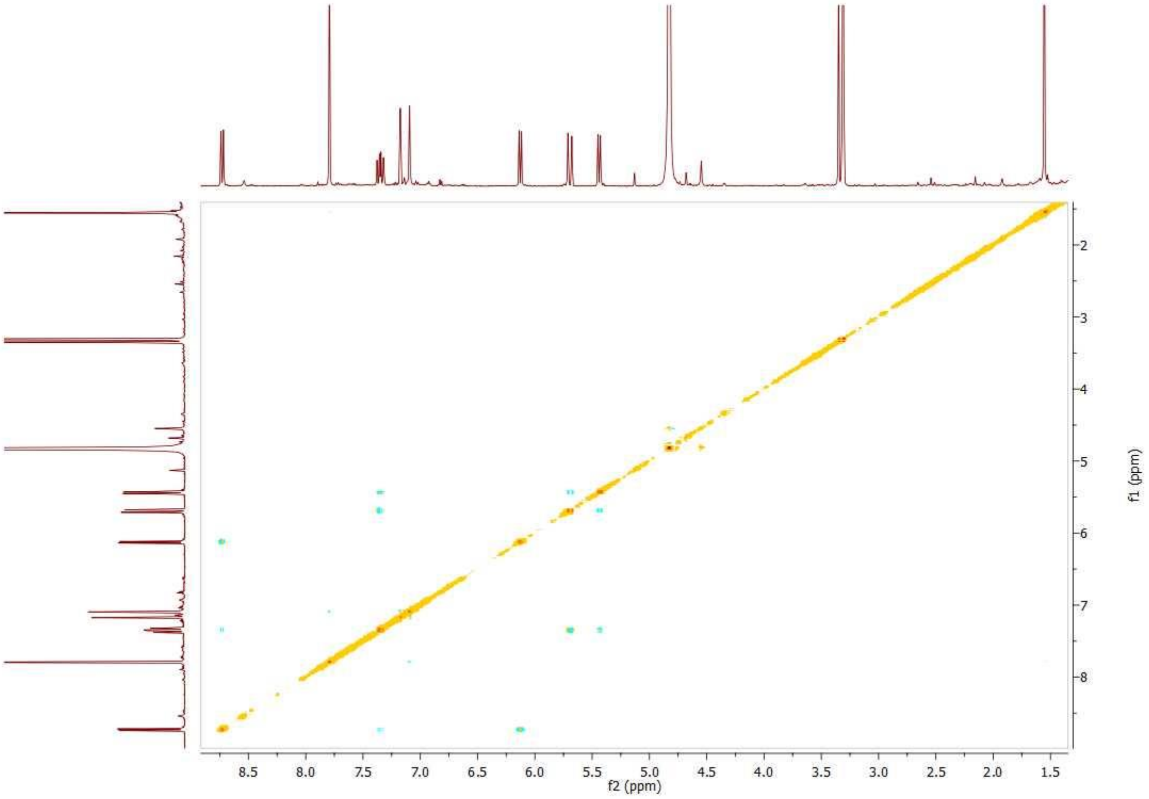

Figure S6. NOESY spectrum of compound **1** (in CD3OD).

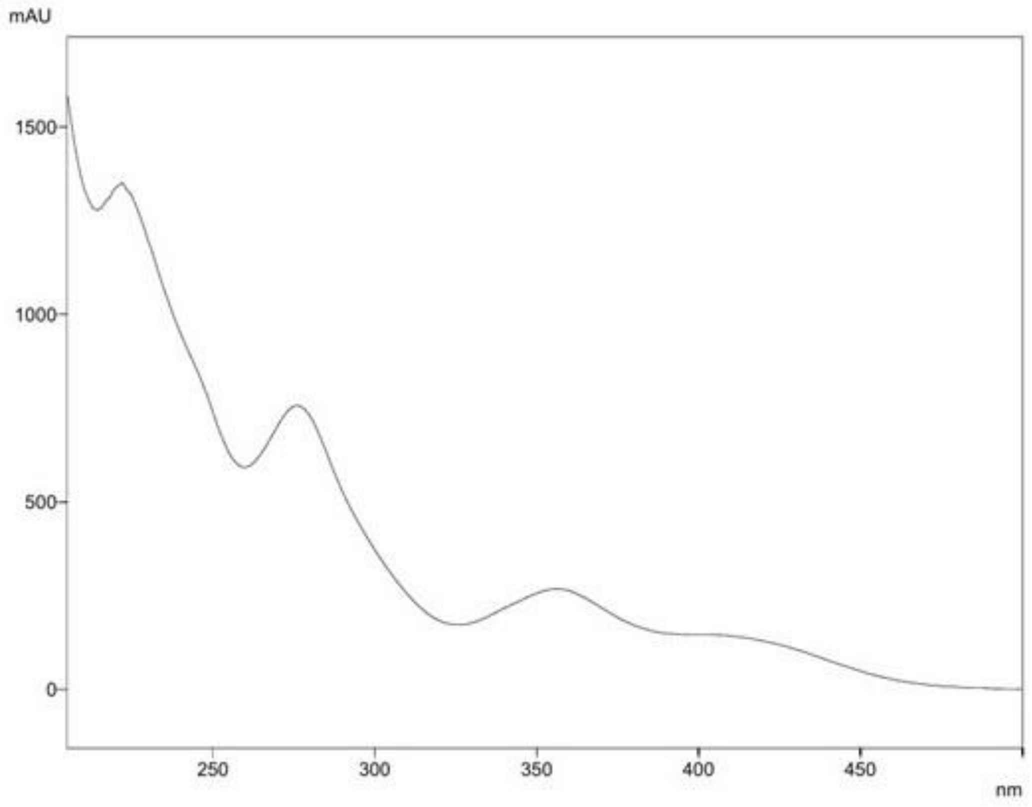

Figure S7. UV spectrum of compound **1** in MeOH.

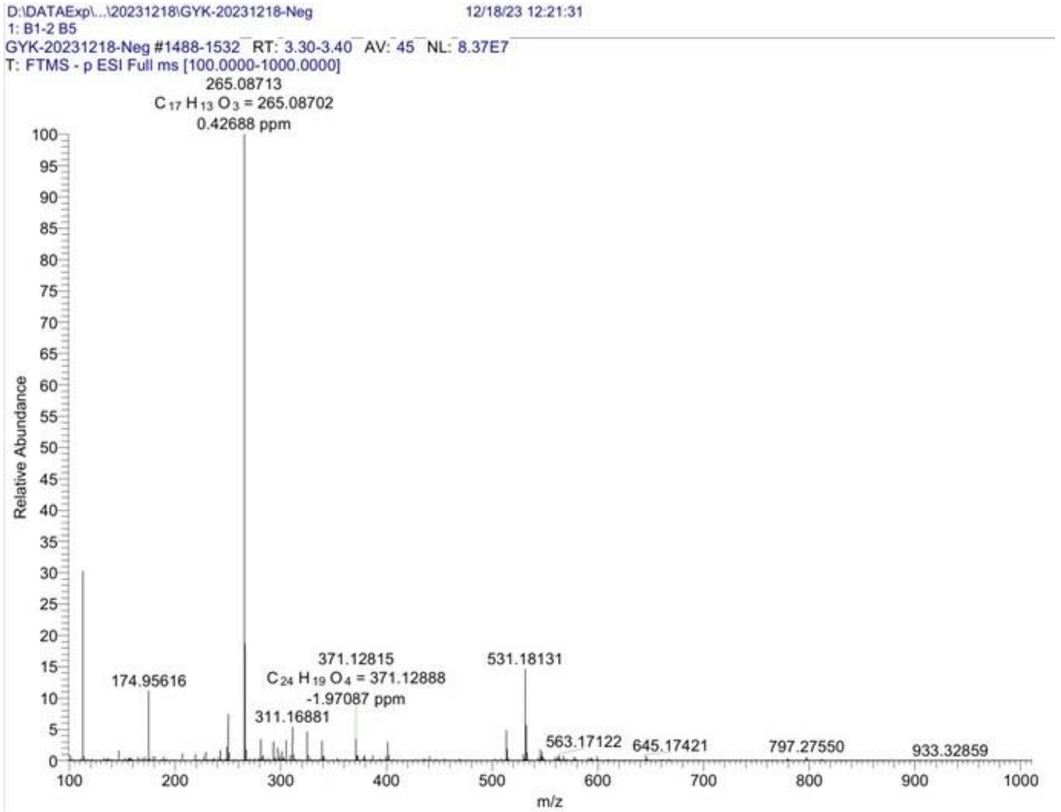

Figure S8. (-)-HRESIMS spectrum of compound **1**.

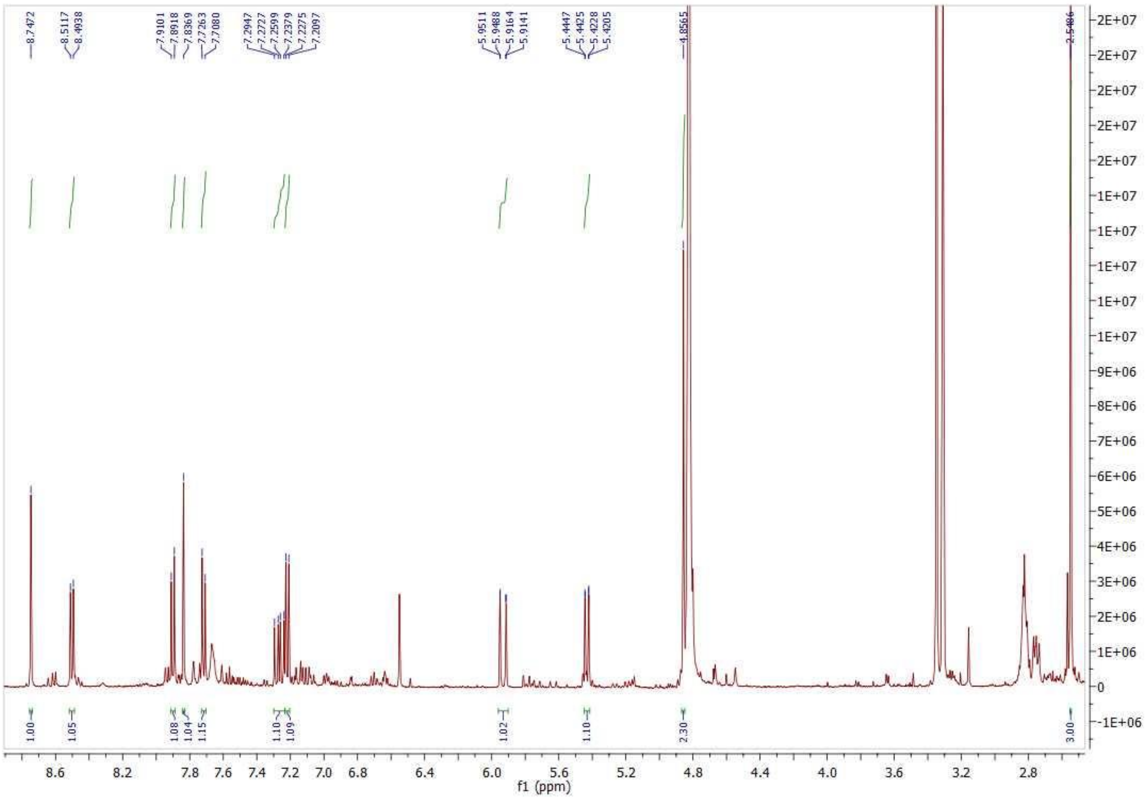

Figure S9. ^{1}H NMR spectrum of compound **2** (500 MHz, in CD_3OD).

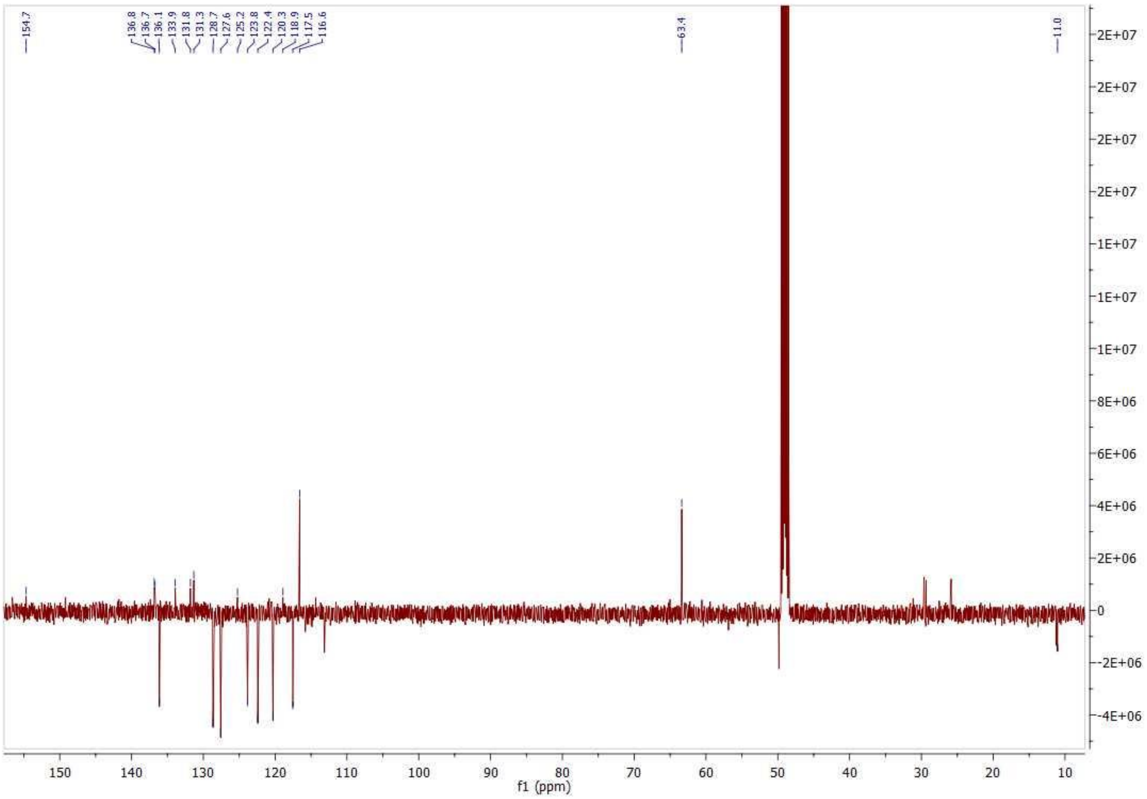

Figure S10. ^{13}C (JMOD) NMR spectrum of compound **2** (125 MHz, in CD_3OD).

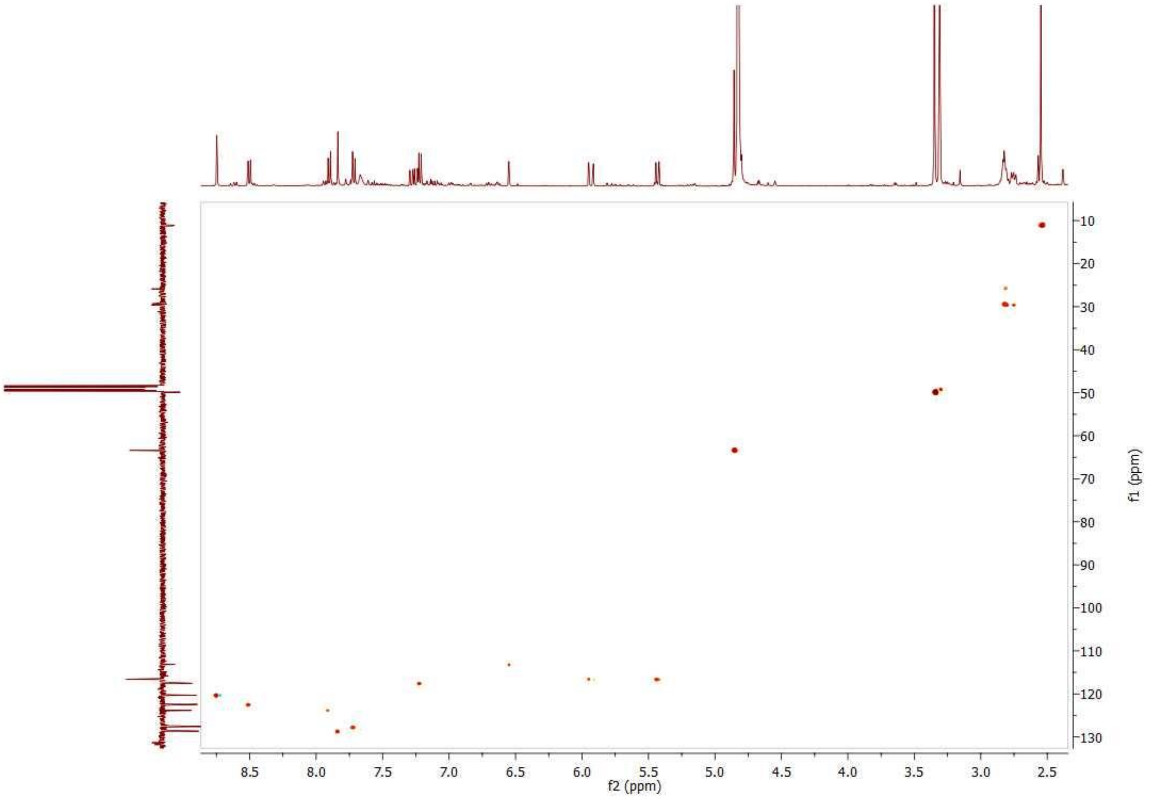

Figure S11. HSQC spectrum of compound **2** (in CD_3OD).

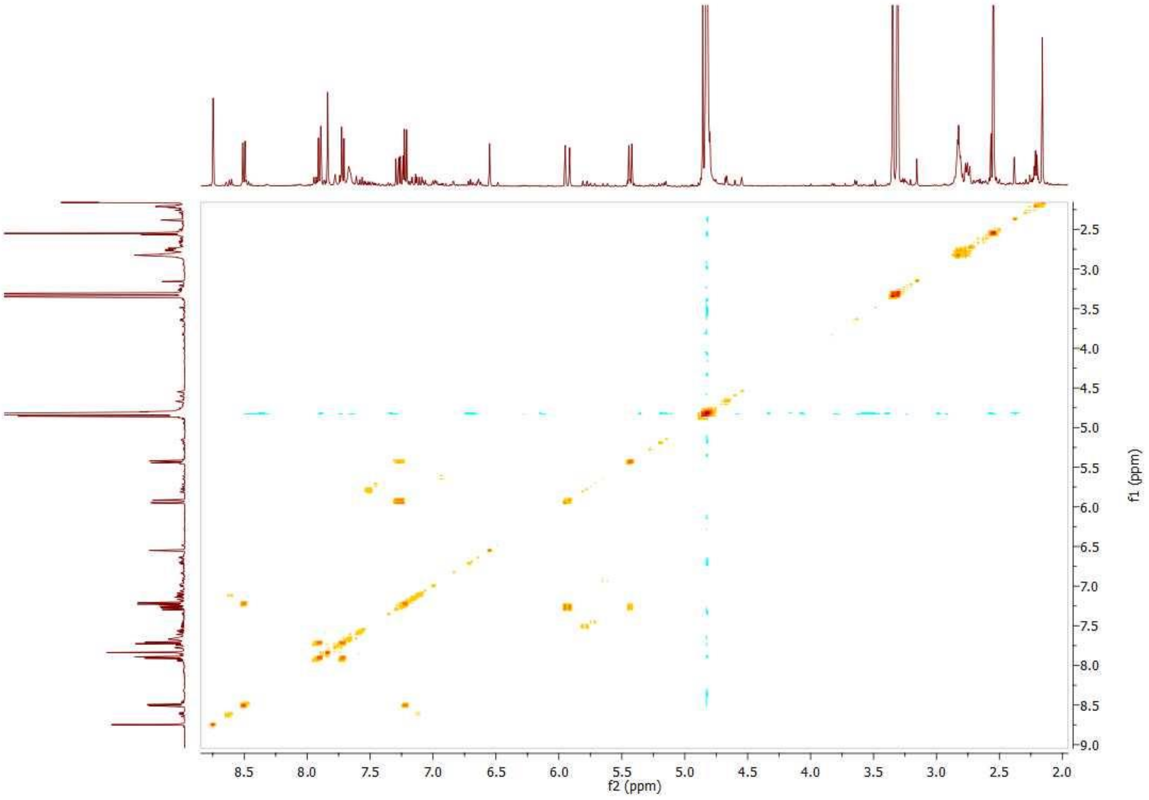

Figure S12. ^1H-^1H COSY spectrum of compound **2** (in CD_3OD).

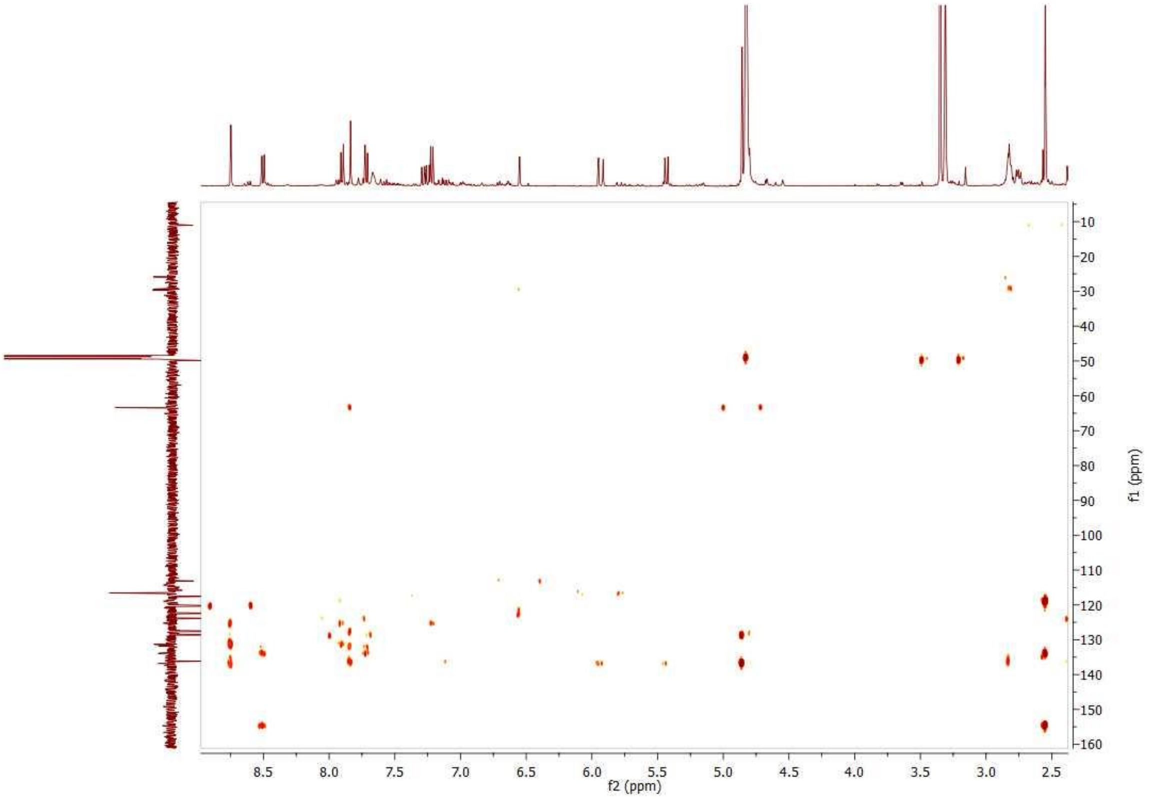

Figure S13. HMBC spectrum of compound **2** (in CD_3OD).

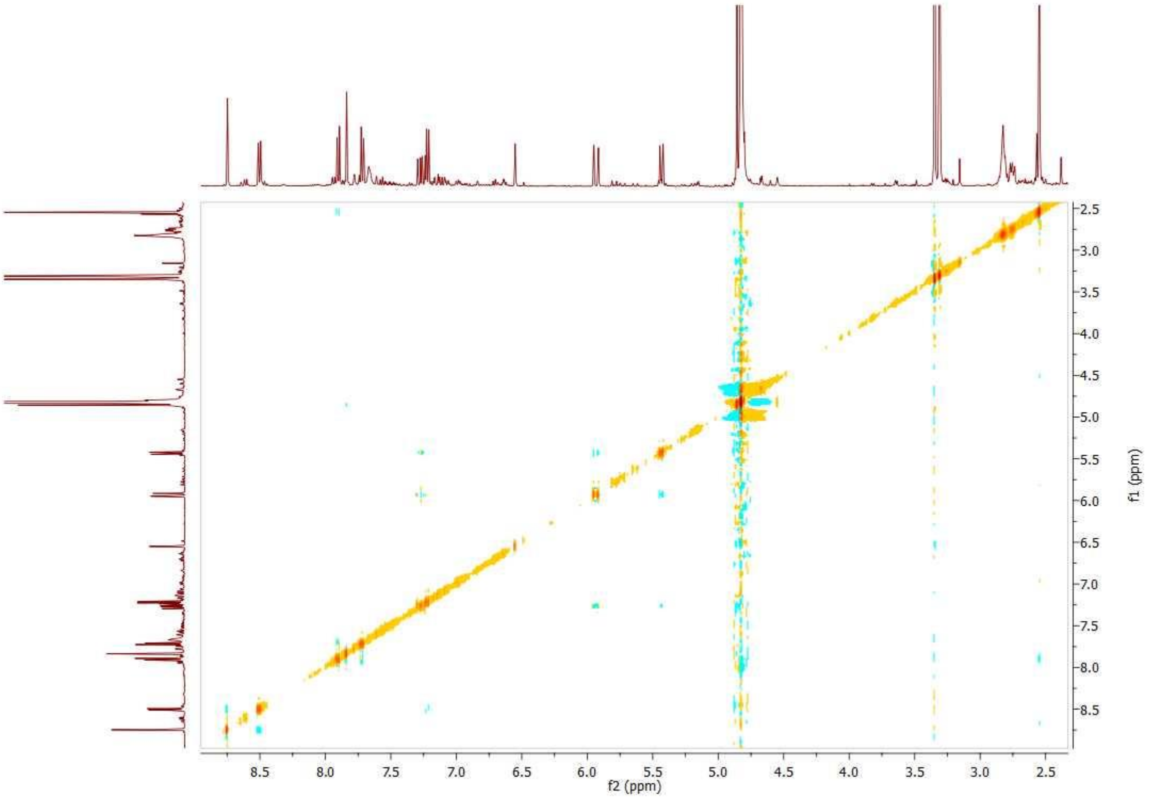

Figure S14. NOESY spectrum of compound **2** (in CD_3OD).

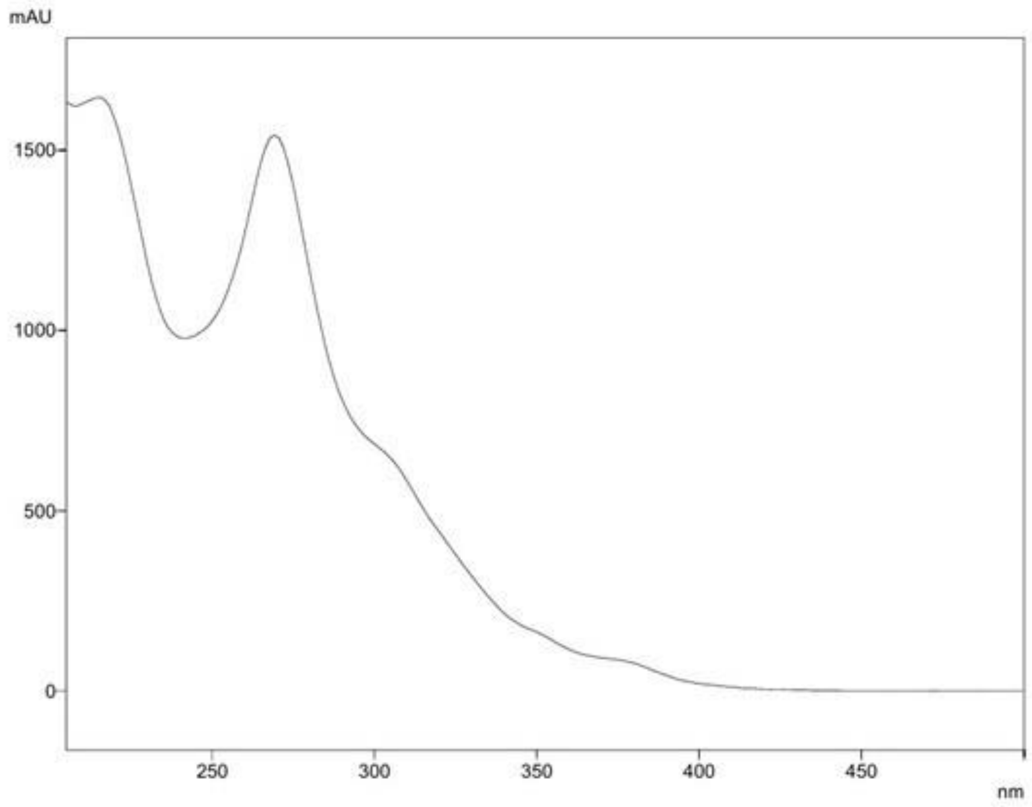

Figure S15. UV spectrum of compound **2** in MeOH.

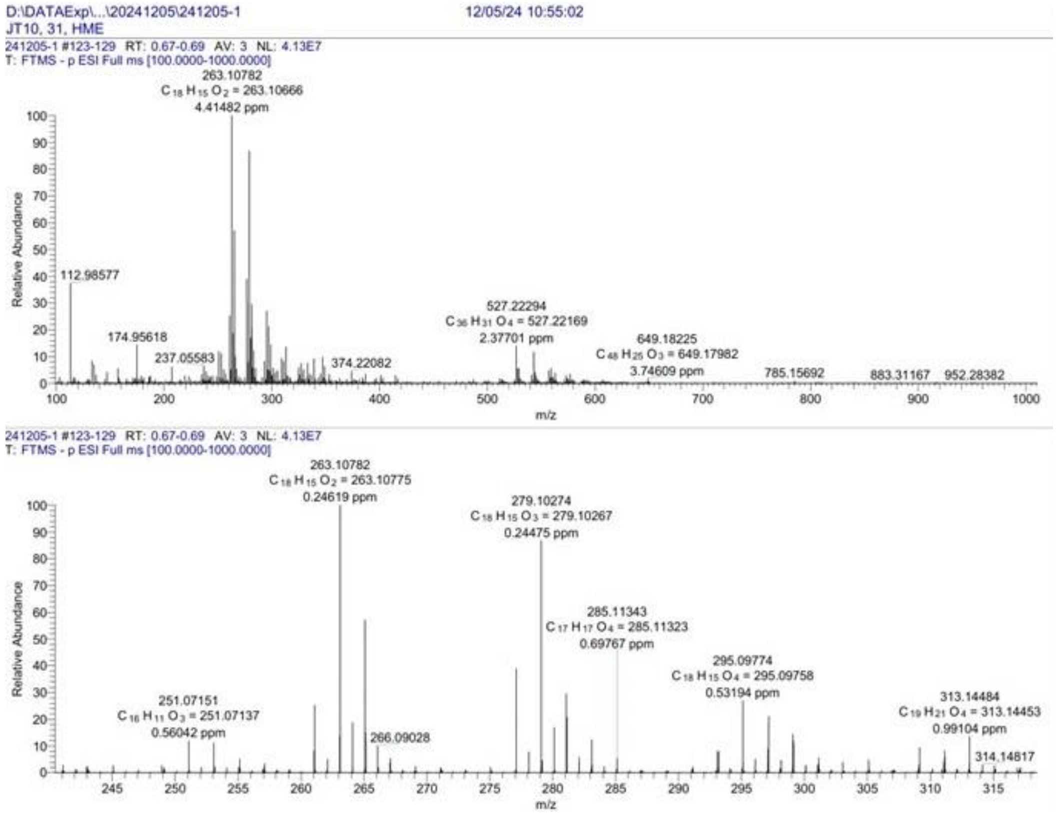

Figure S16. (-)-HRESIMS spectrum of compound **2**.

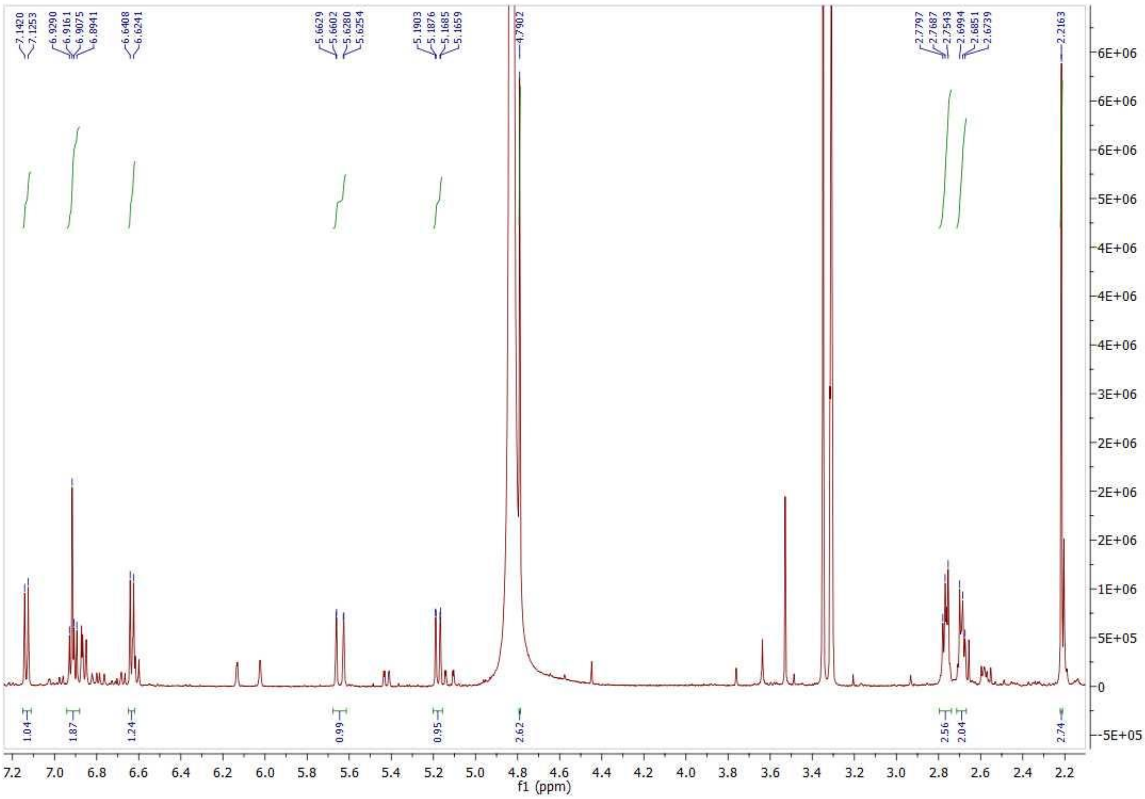

Figure S17. ^{1}H NMR spectrum of compound **3** (500 MHz, in CD_3OD).

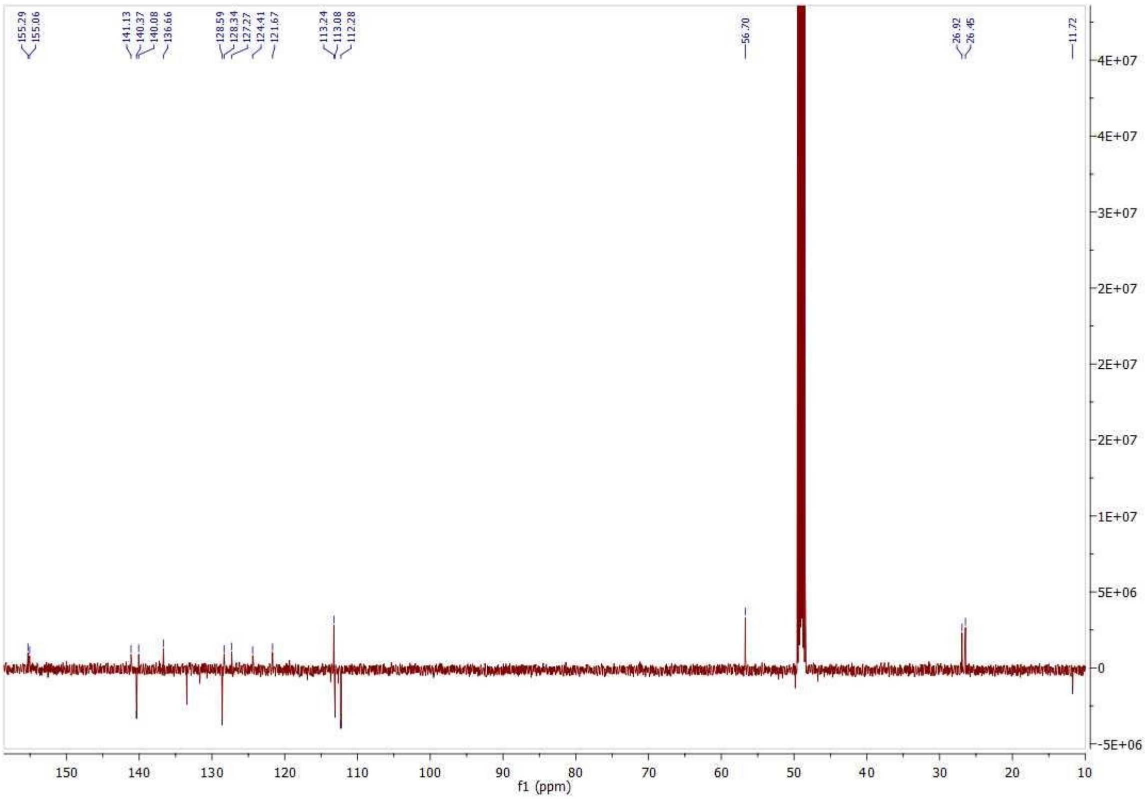

Figure S18. ^{13}C (JMOD) NMR spectrum of compound **3** (125 MHz, in CD_3OD).

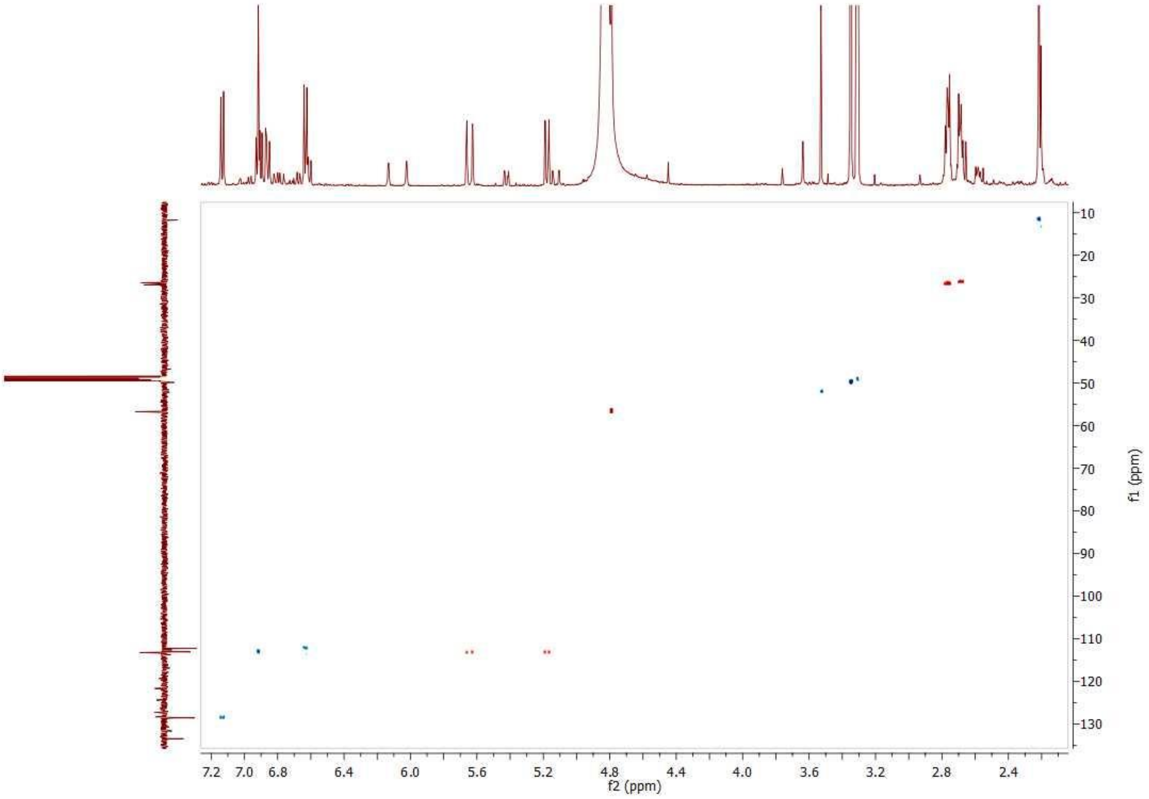

Figure S19. HSQC spectrum of compound **3** (in CD_3OD).

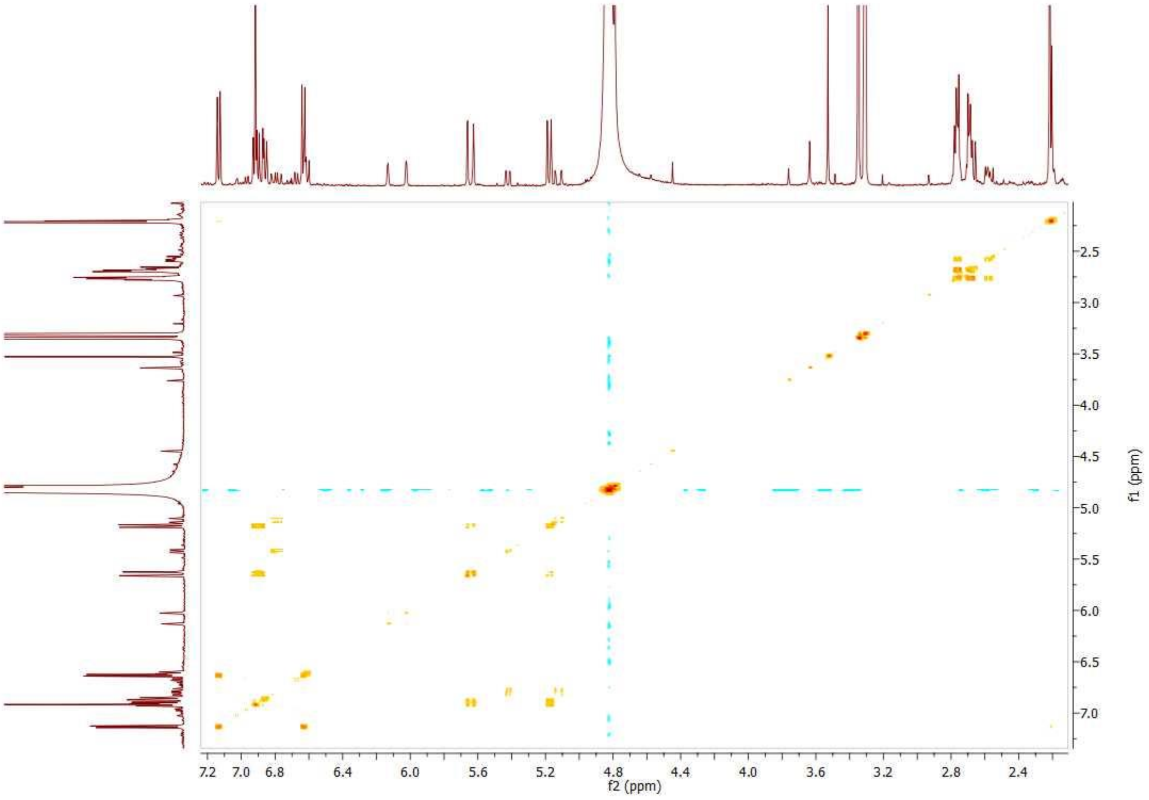

Figure S20. ^1H-^1H COSY spectrum of compound **3** (in CD_3OD).

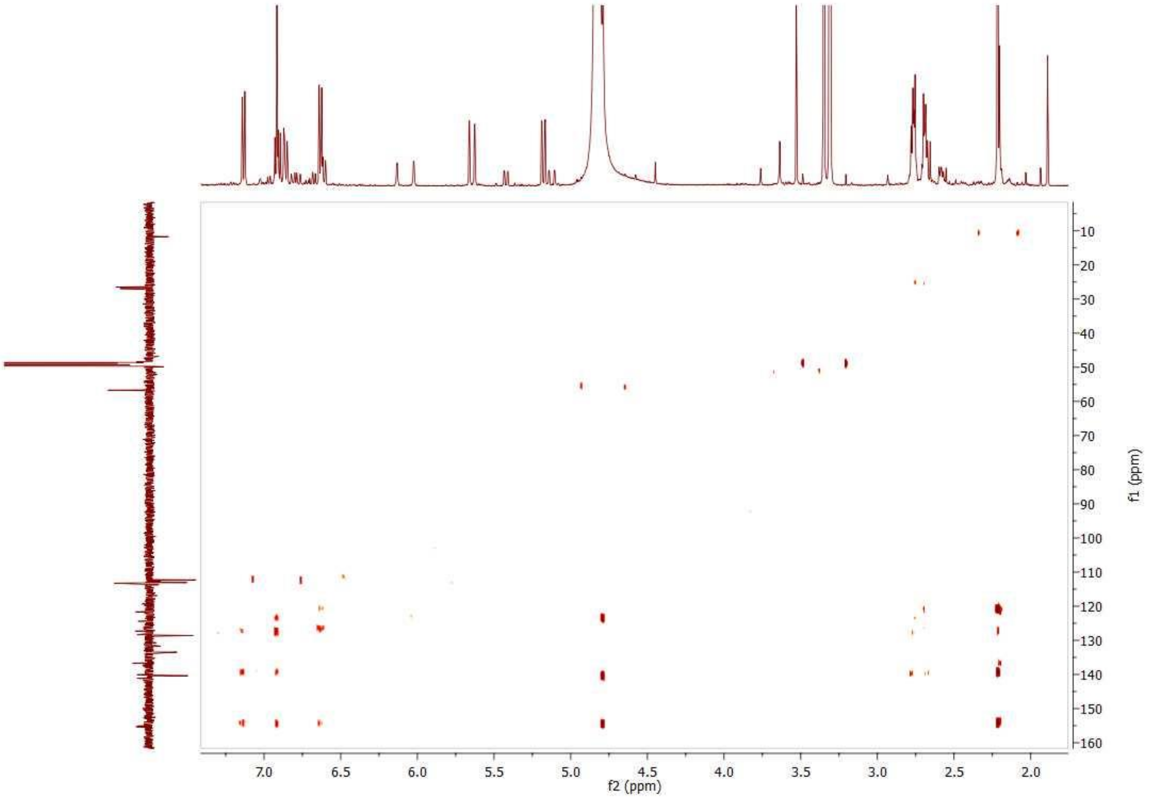

Figure S21. HMBC spectrum of compound **3** (in CD_3OD).

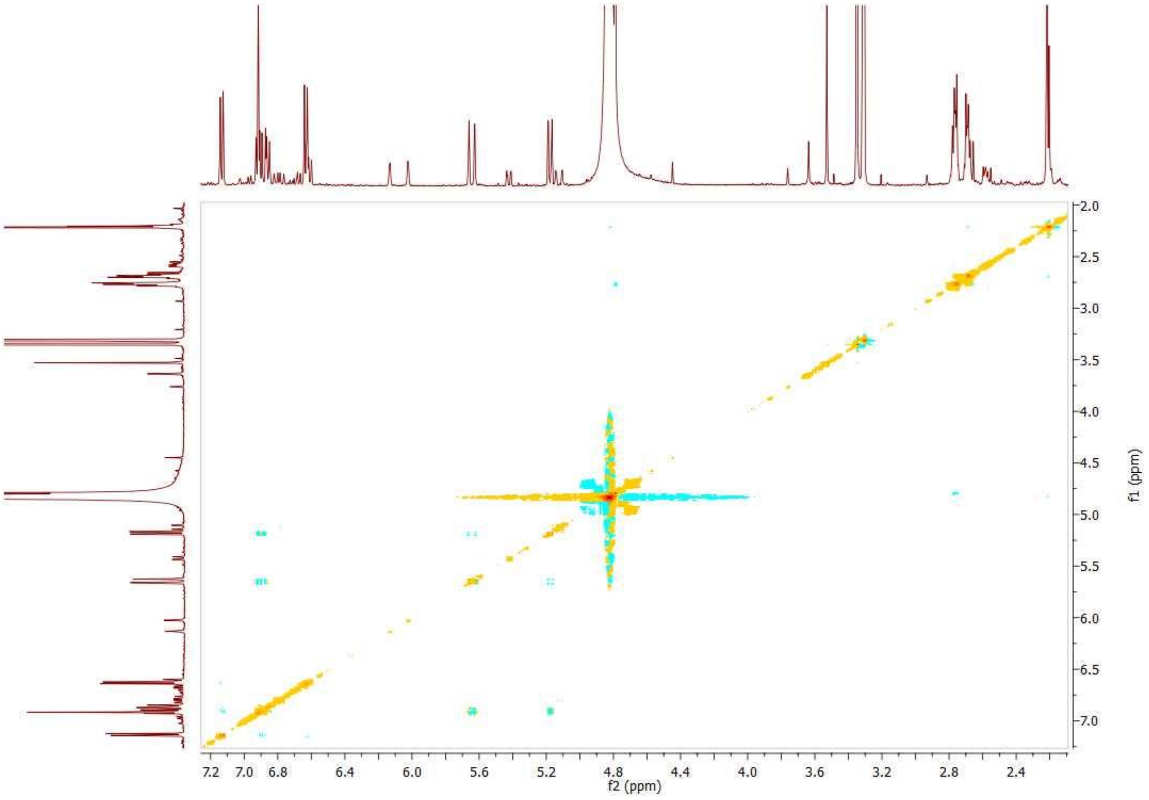

Figure S22. NOESY spectrum of compound **3** (in CD_3OD).

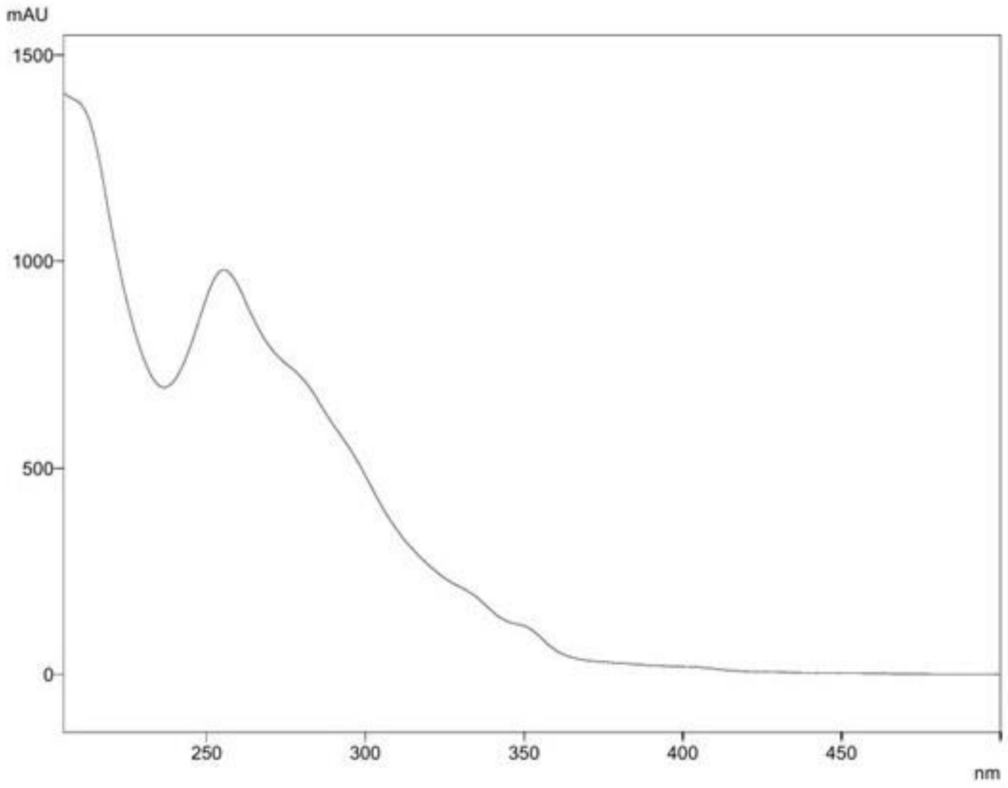

Figure S23. UV spectrum of compound **3** in MeOH.

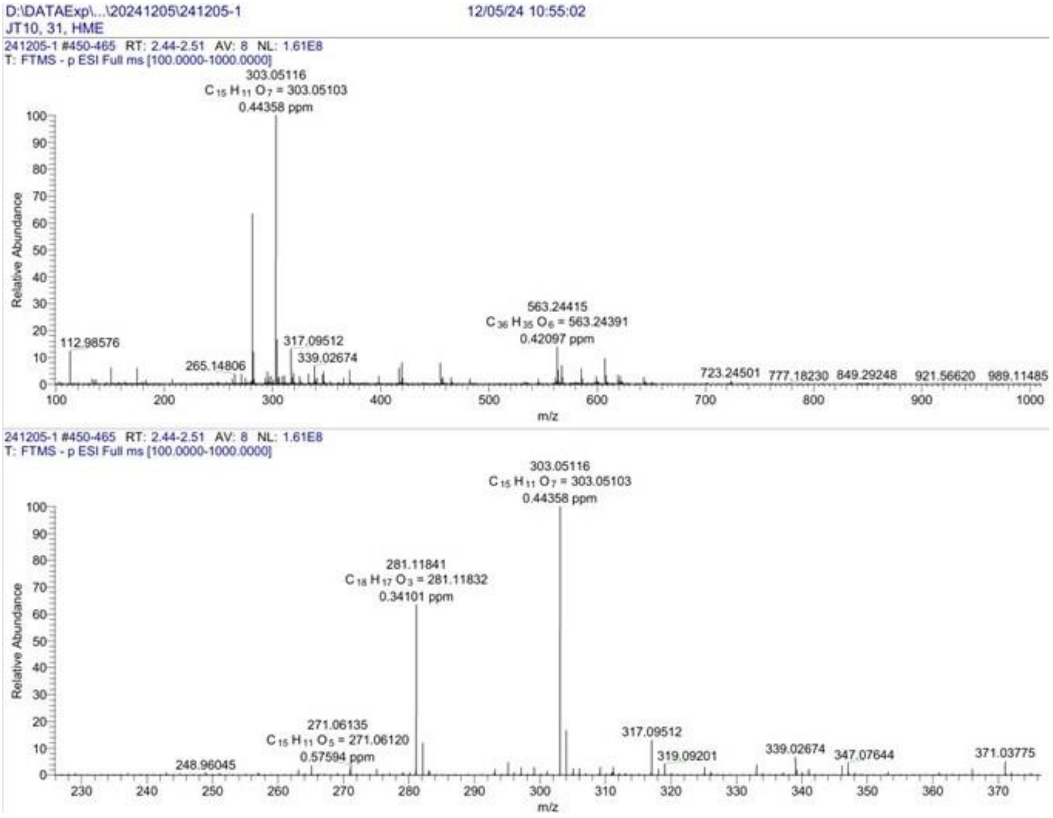

Figure S24. (-)-HRESIMS spectrum of compound **3**.

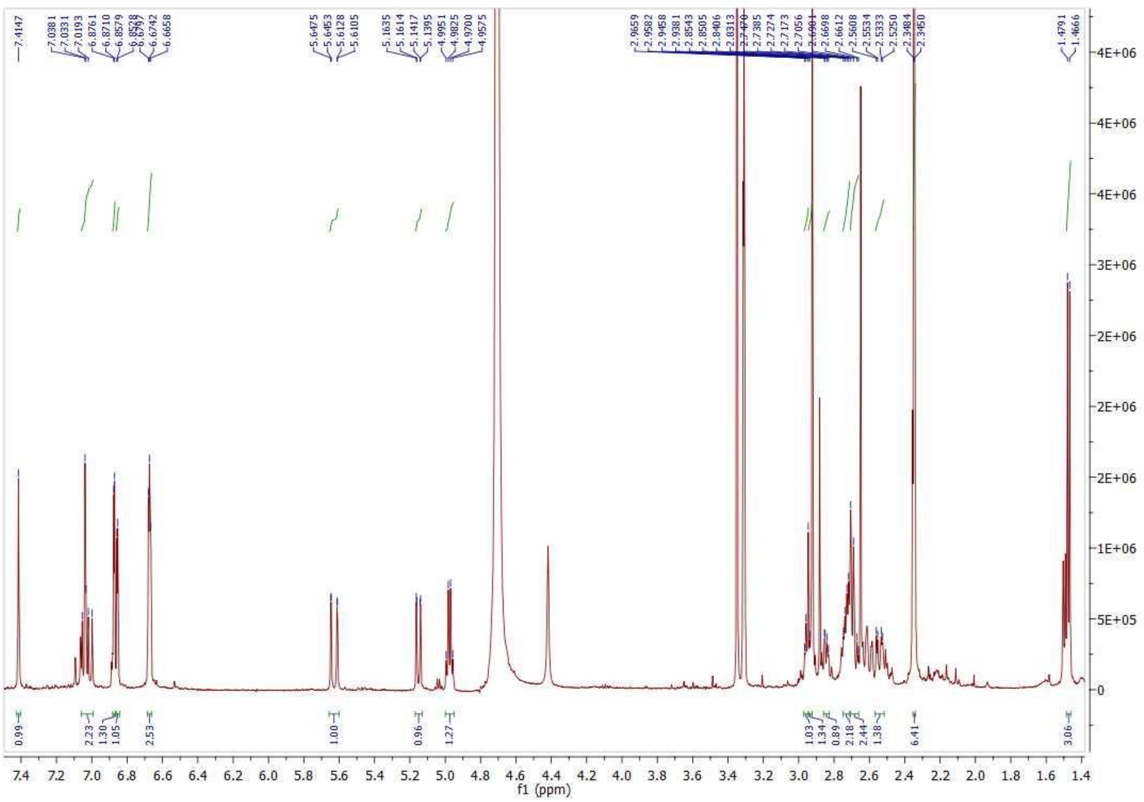

Figure S25. ^{1}H NMR spectrum of compound **4** (500 MHz, in CD_3OD).

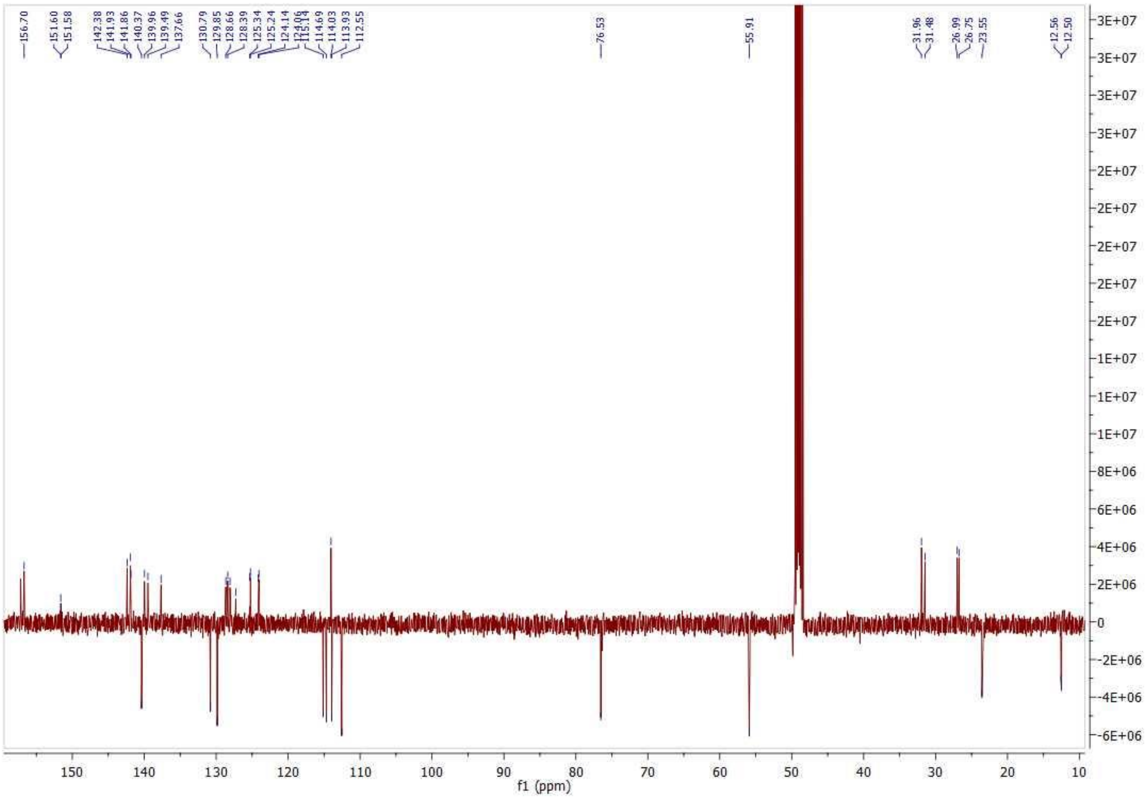

Figure S26. ^{13}C (JMOD) NMR spectrum of compound **4** (125 MHz, in CD_3OD).

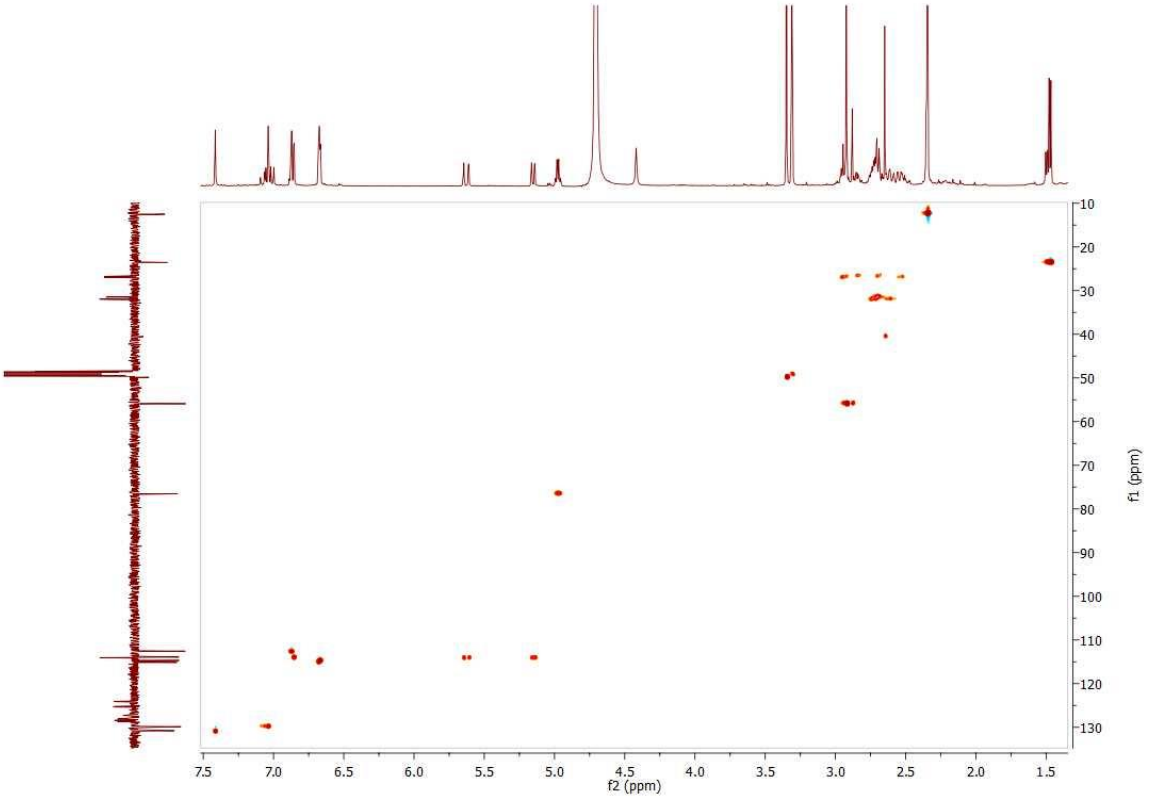

Figure S27. HSQC spectrum of compound **4** (in CD_3OD).

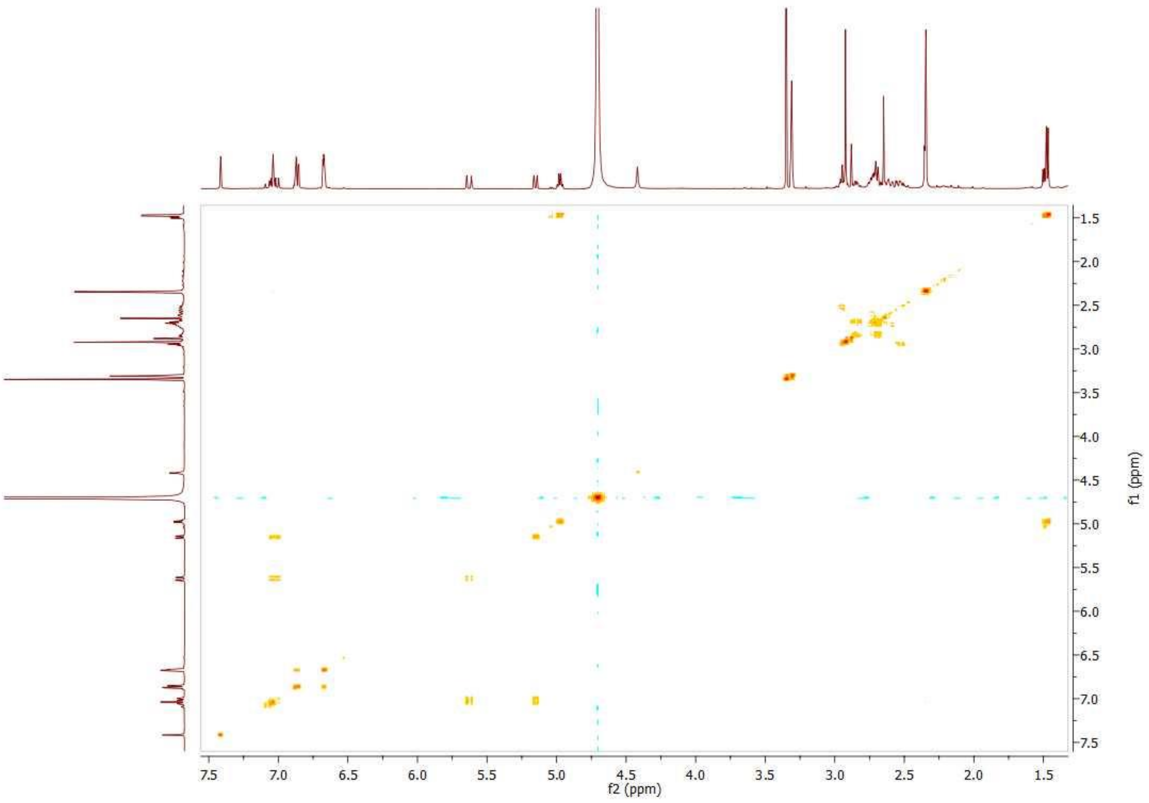

Figure S28. ^1H-^1H COSY spectrum of compound **4** (in CD_3OD).

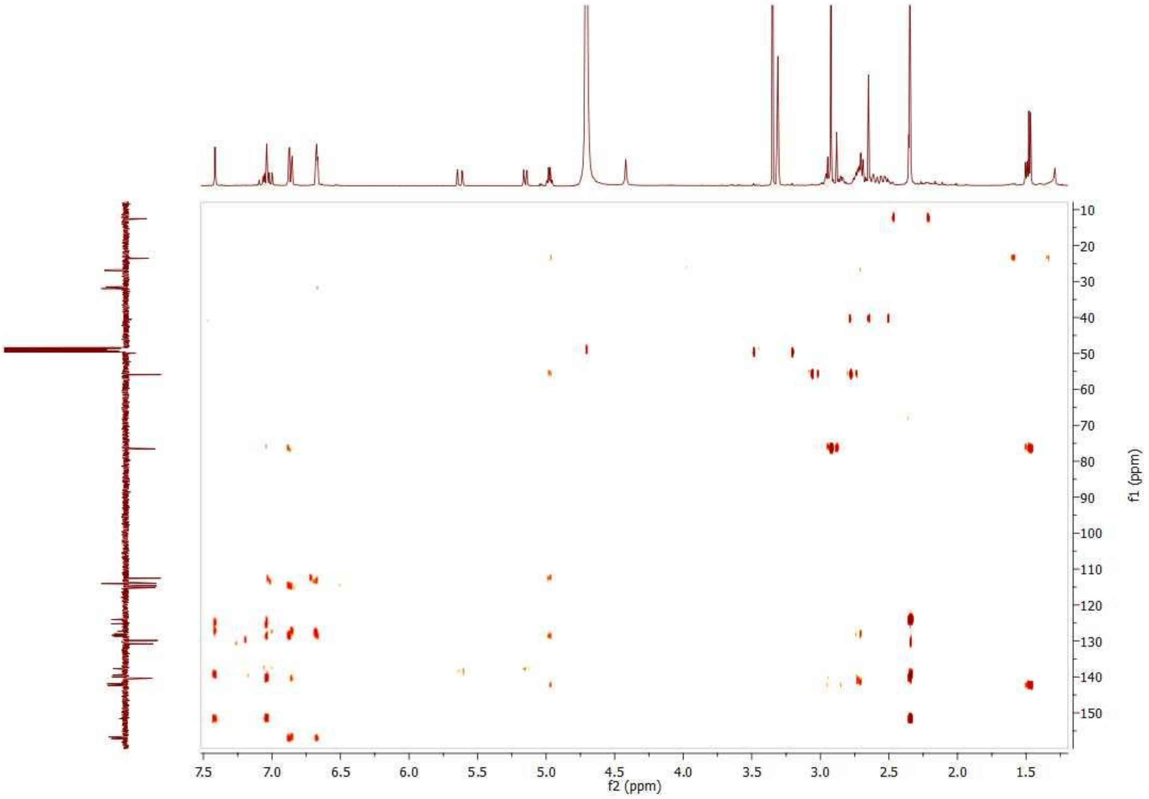

Figure S29. HMBC spectrum of compound **4** (in CD_3OD).

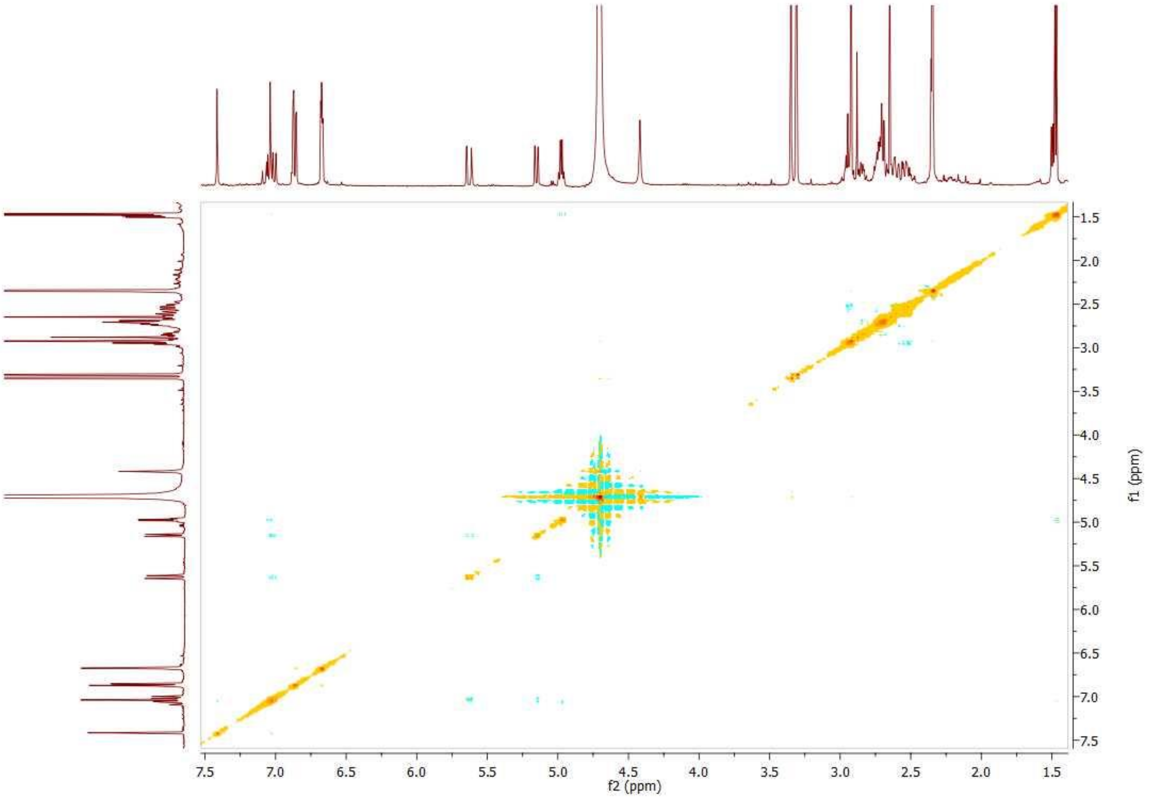

Figure S30. NOESY spectrum of compound **4** (in CD_3OD).

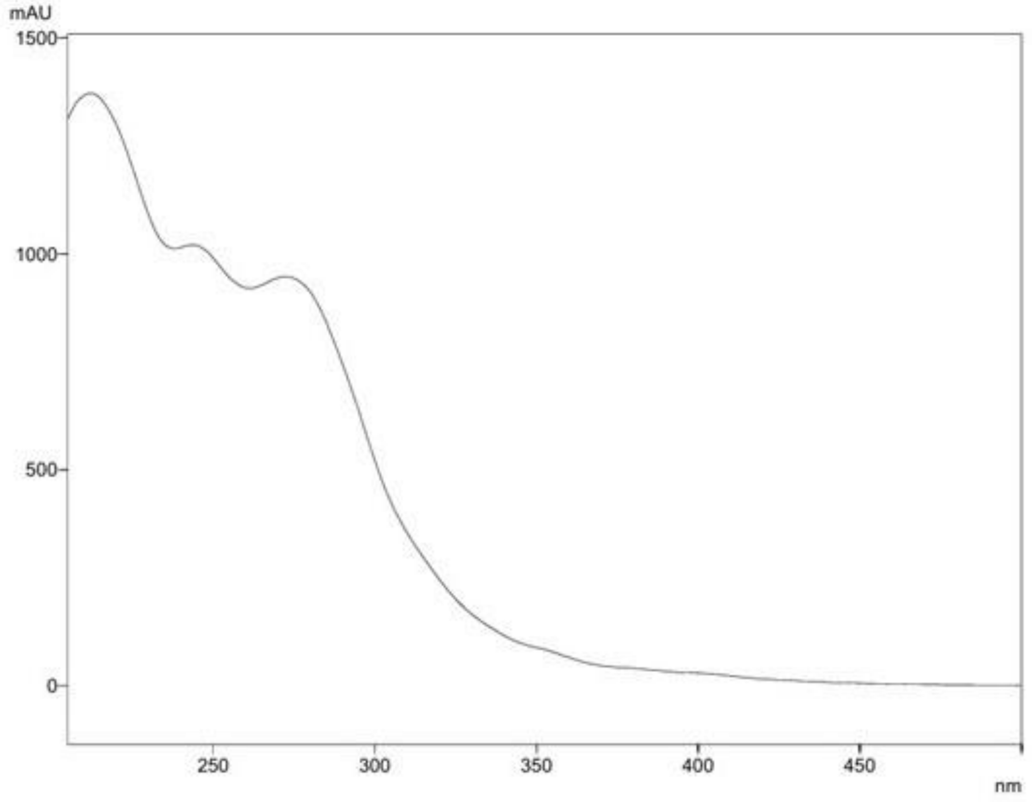

Figure S31. UV spectrum of compound **4** in MeOH.

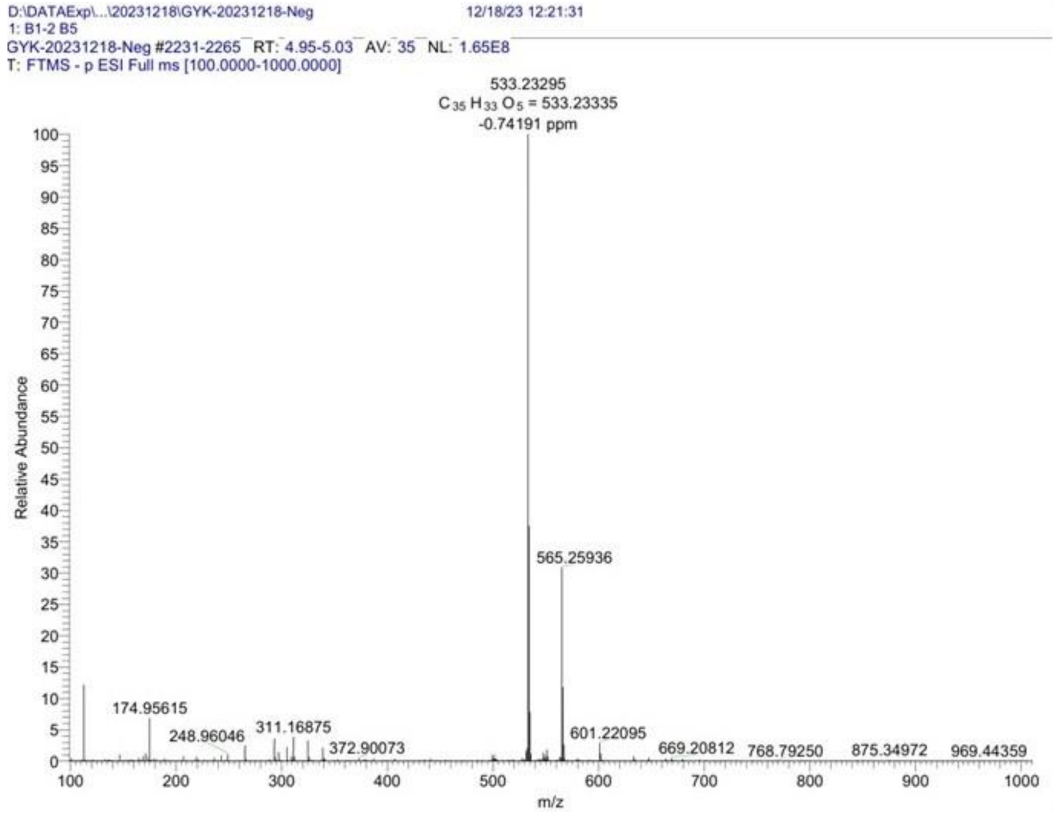

Figure S32. (-)-HRESIMS spectrum of compound **4**.

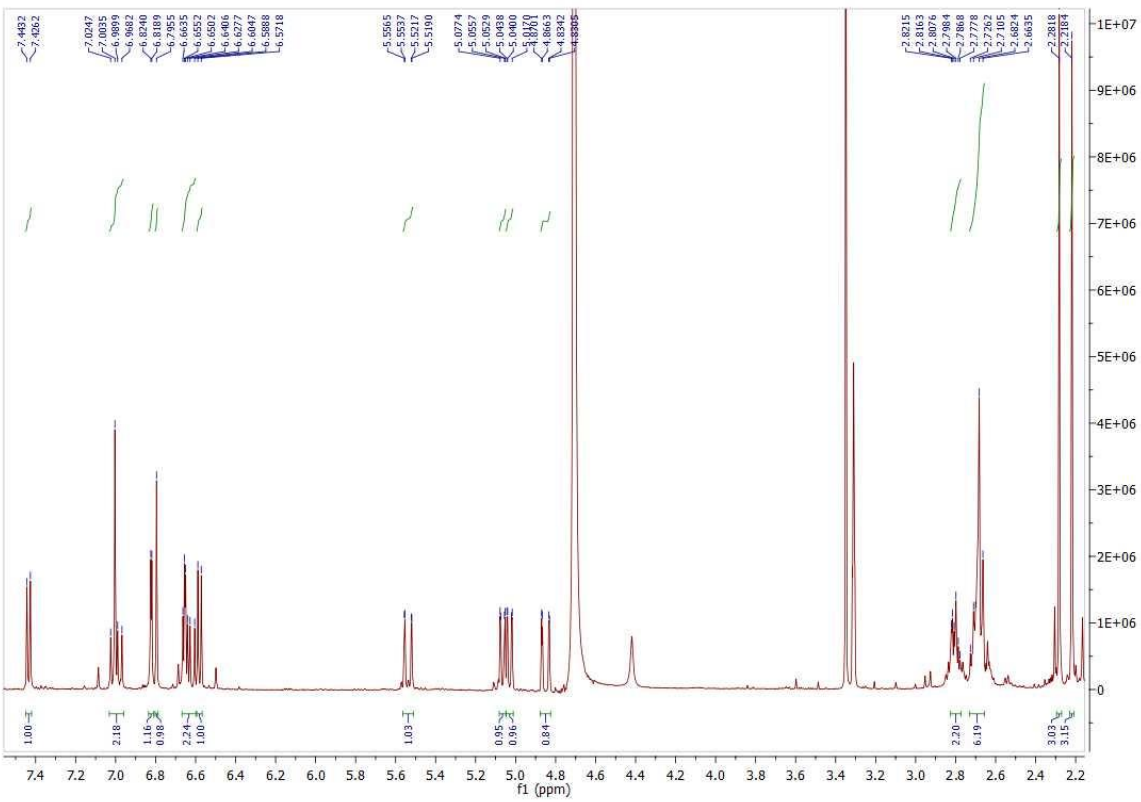

Figure S33. ^{1}H NMR spectrum of compound **5** (500 MHz, in CD_3OD).

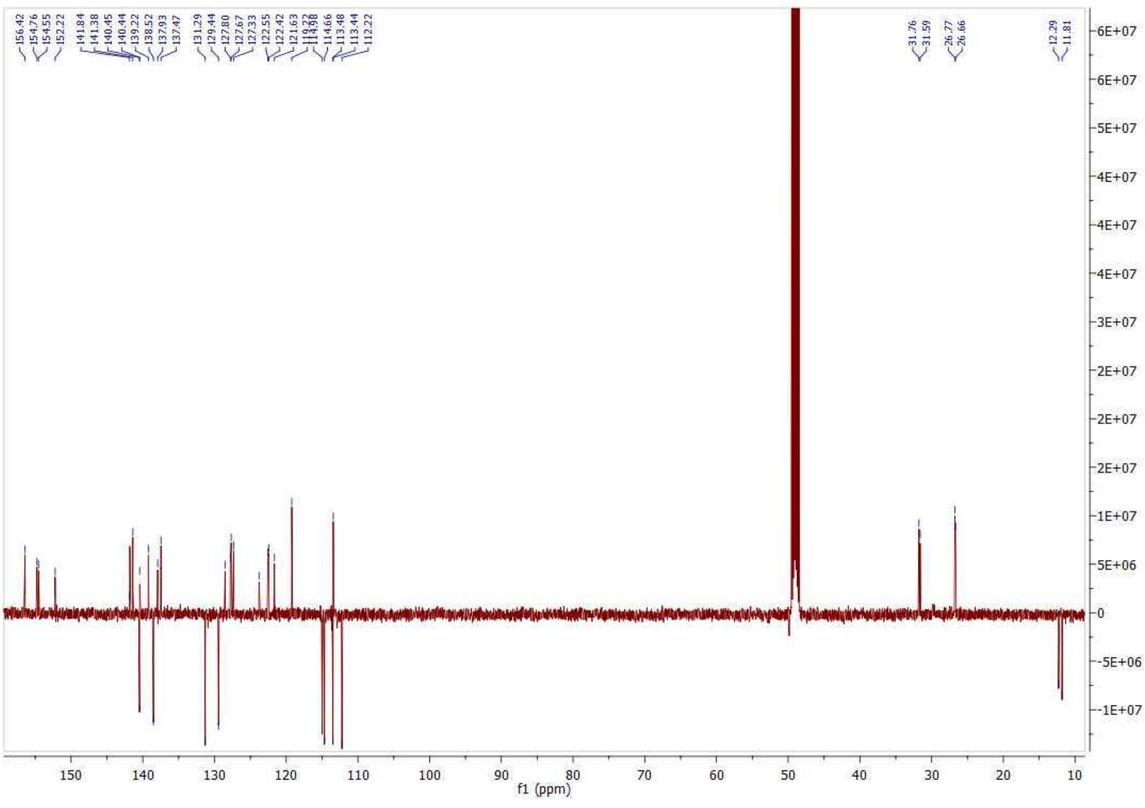

Figure S34. ^{13}C (JMOD) NMR spectrum of compound **5** (125 MHz, in CD_3OD).

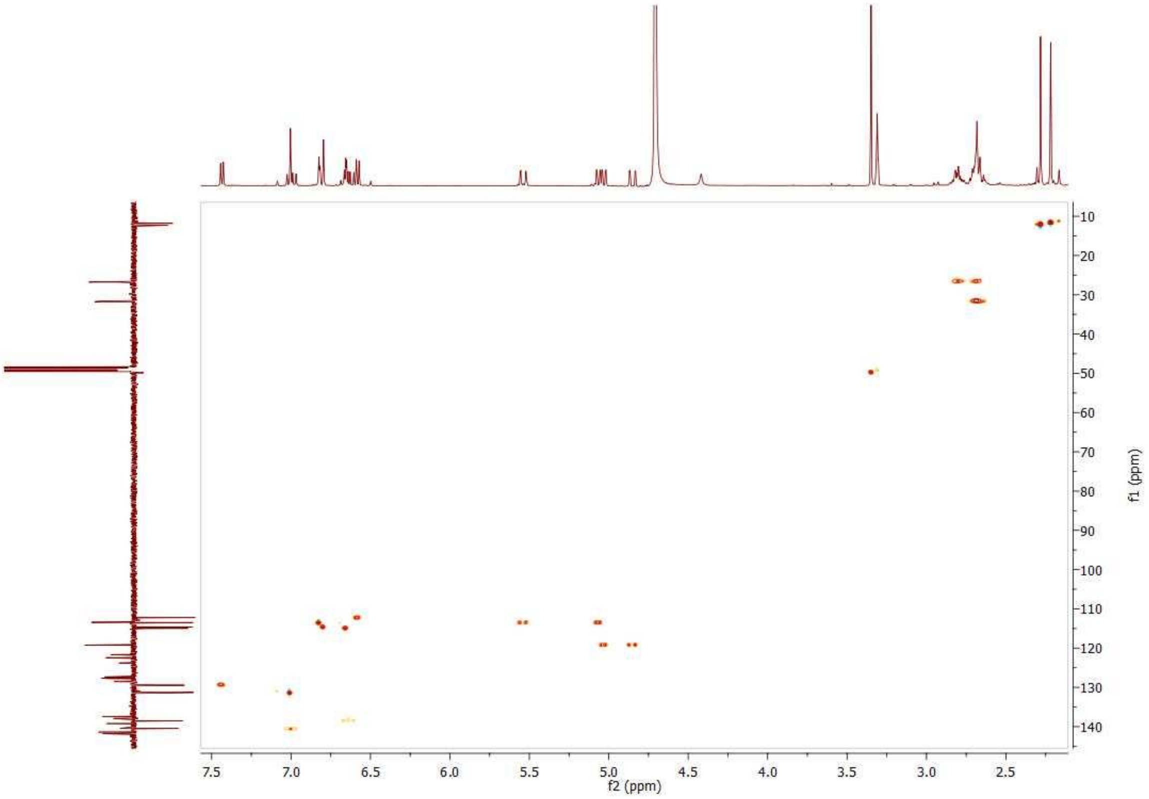

Figure S35. HSQC spectrum of compound **5** (in CD_3OD).

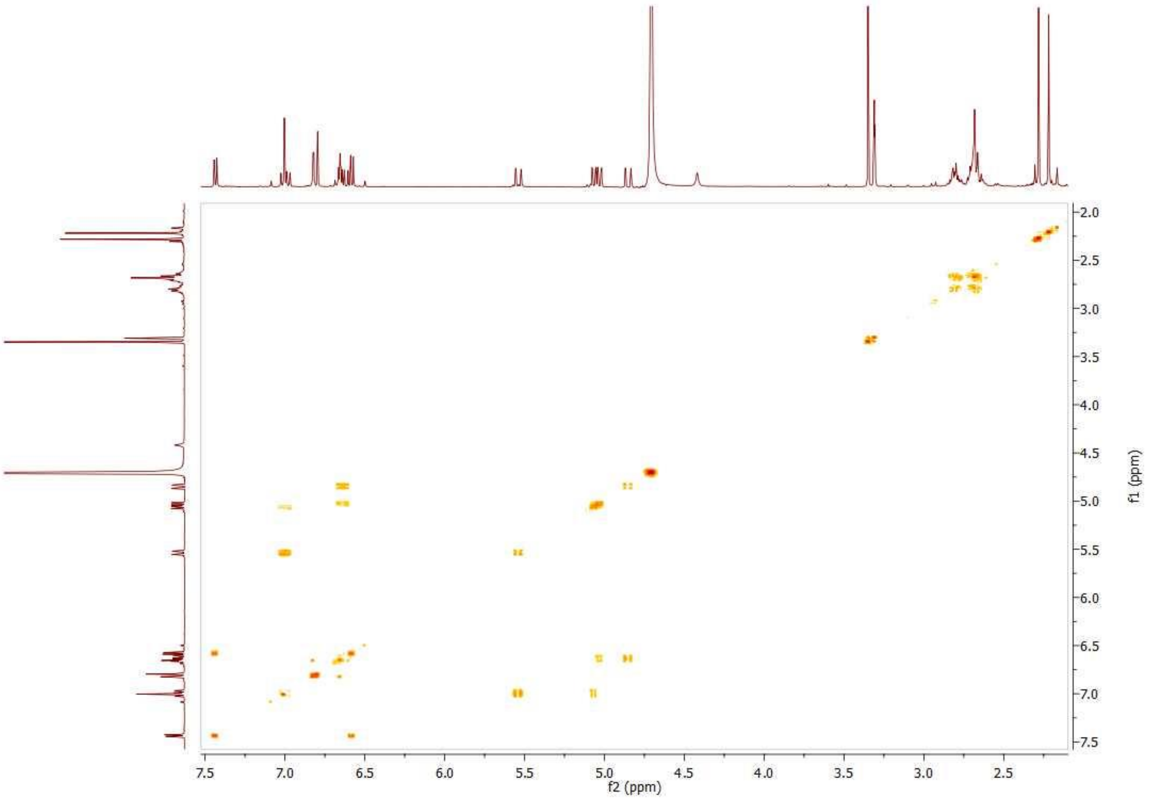

Figure S36. ^1H-^1H COSY spectrum of compound **5** (in CD_3OD).

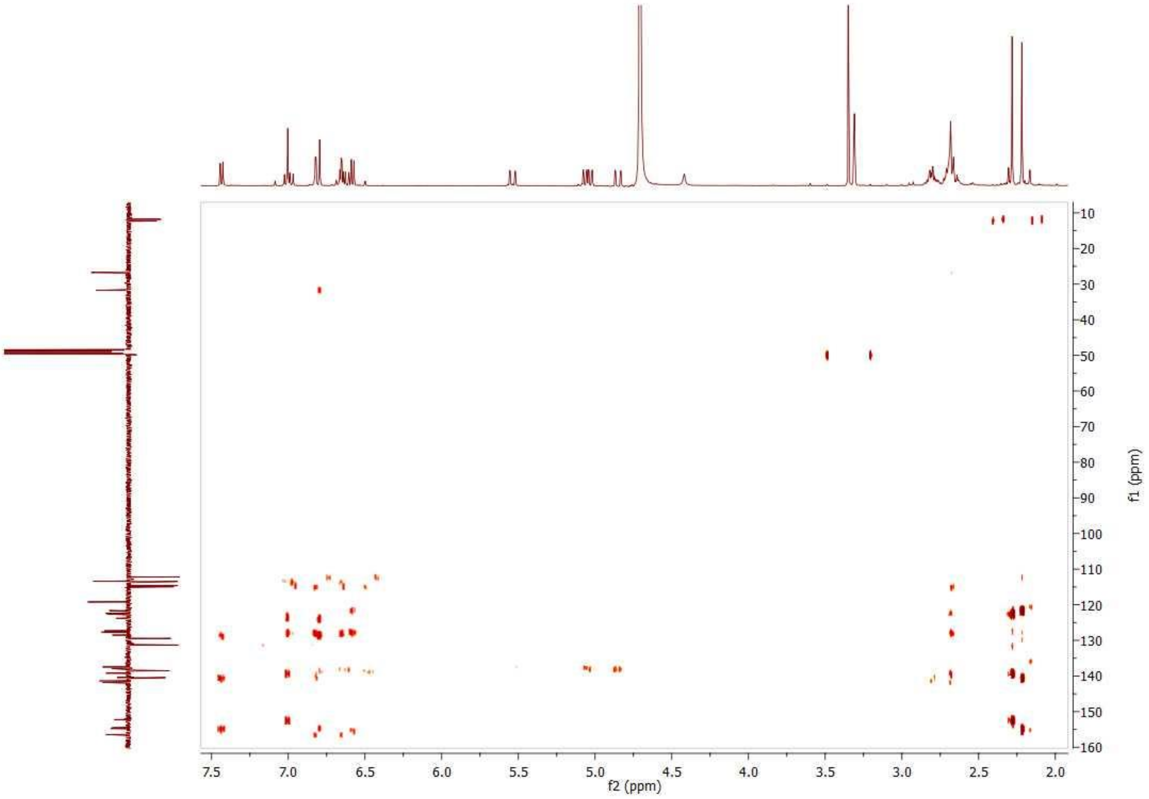

Figure S37. HMBC spectrum of compound **5** (in CD_3OD).

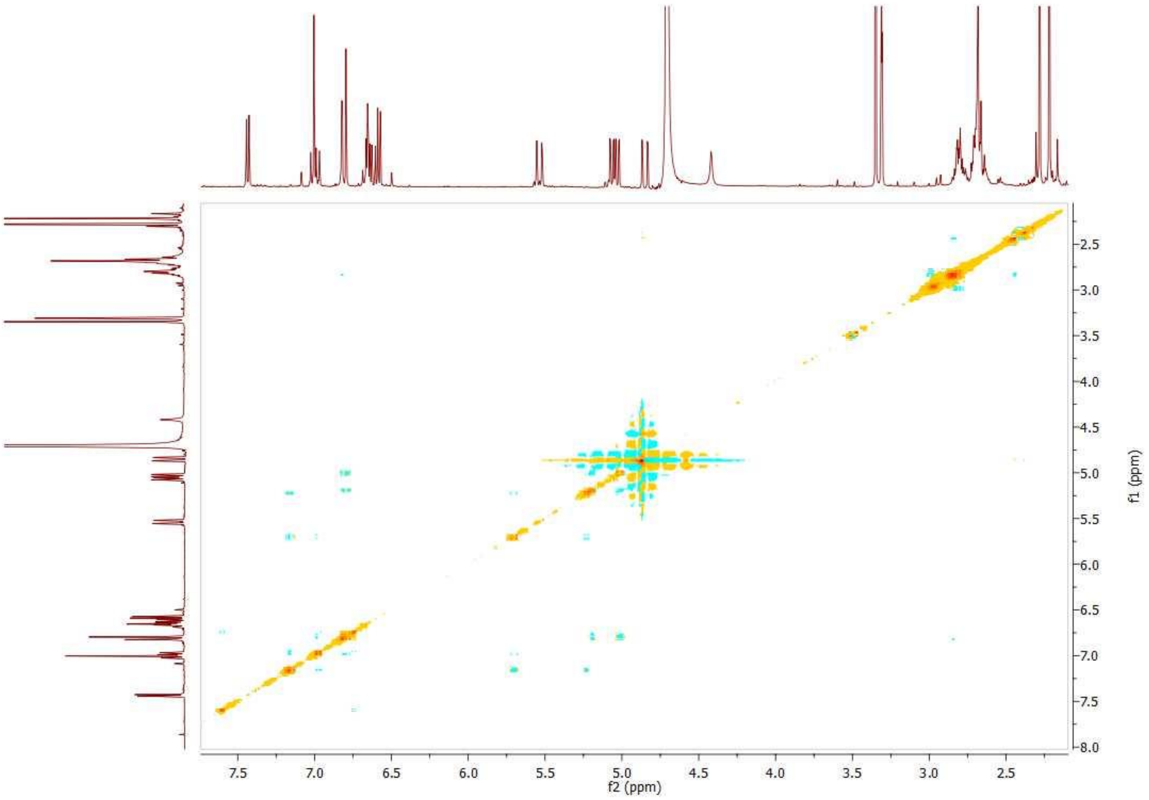

Figure S38. NOESY spectrum of compound **5** (in CD_3OD).

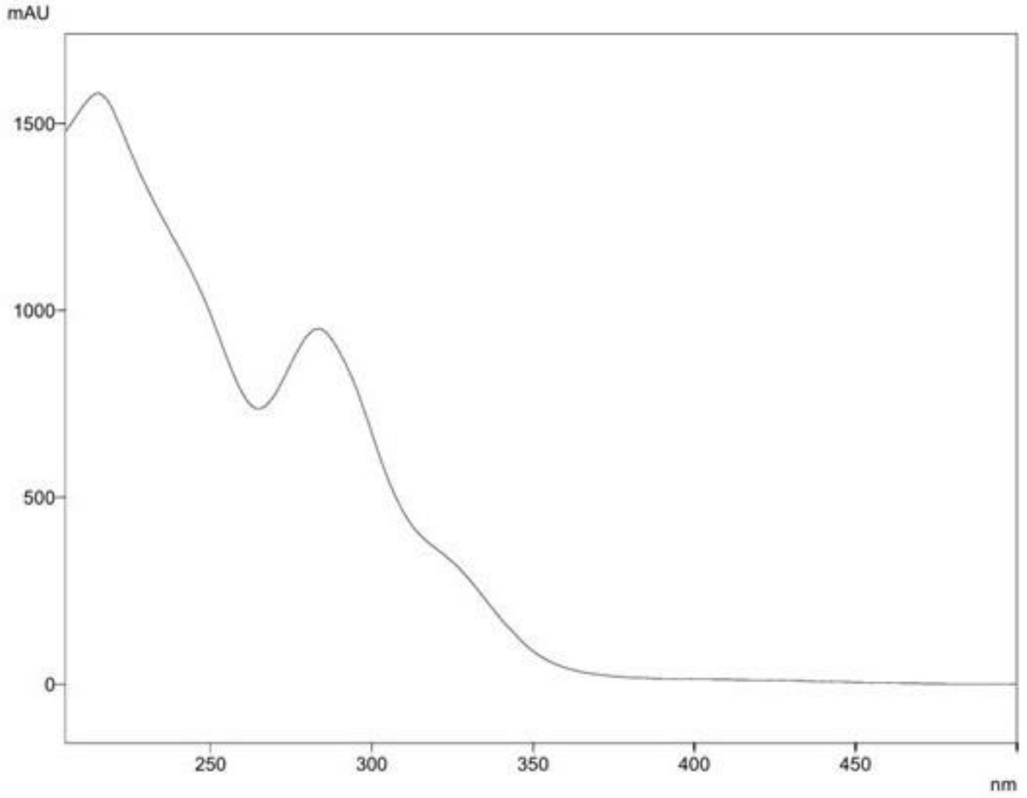

Figure S39. UV spectrum of compound **5** in MeOH.

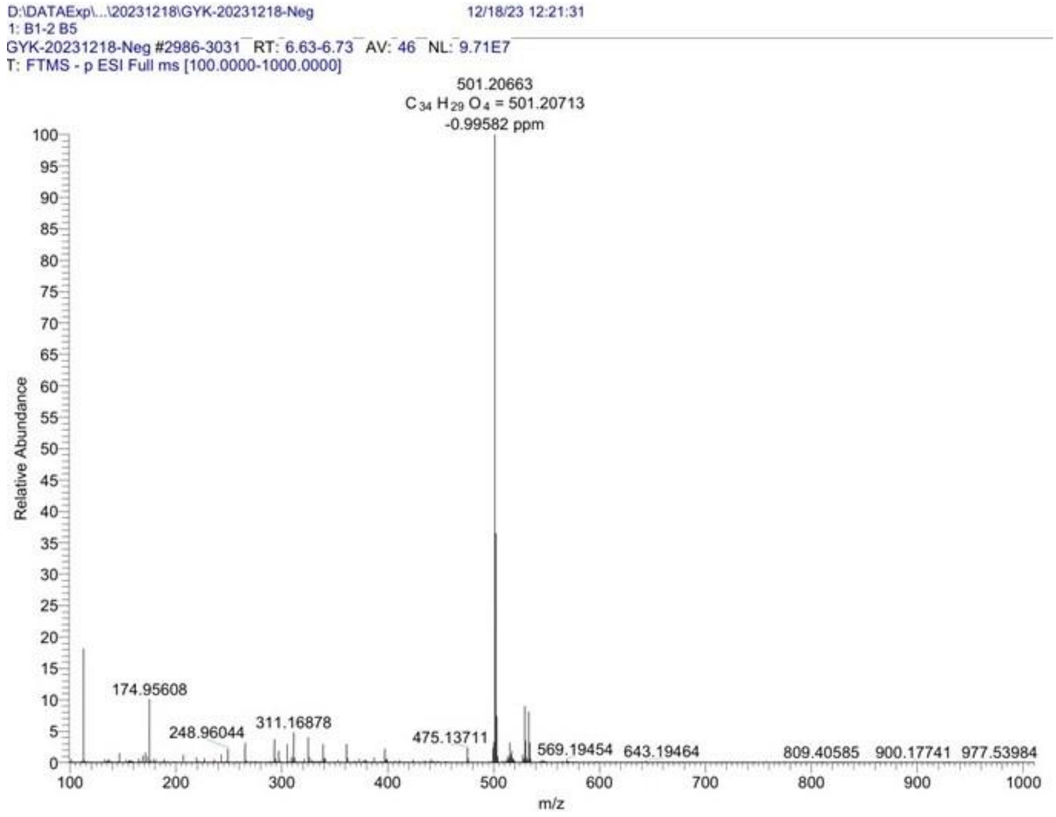

Figure S40. (-)-HRESIMS spectrum of compound **5**.

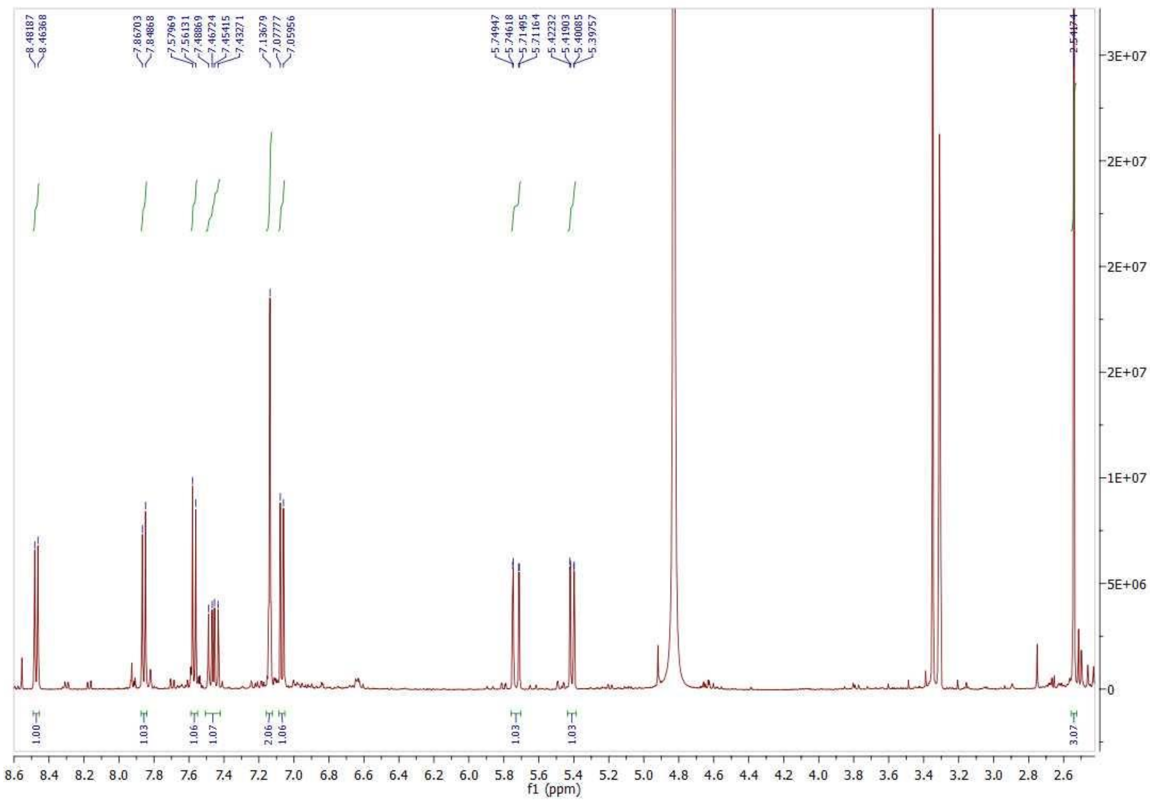

Figure S41. ^{1}H NMR spectrum of compound **6** (500 MHz, in CD_3OD).

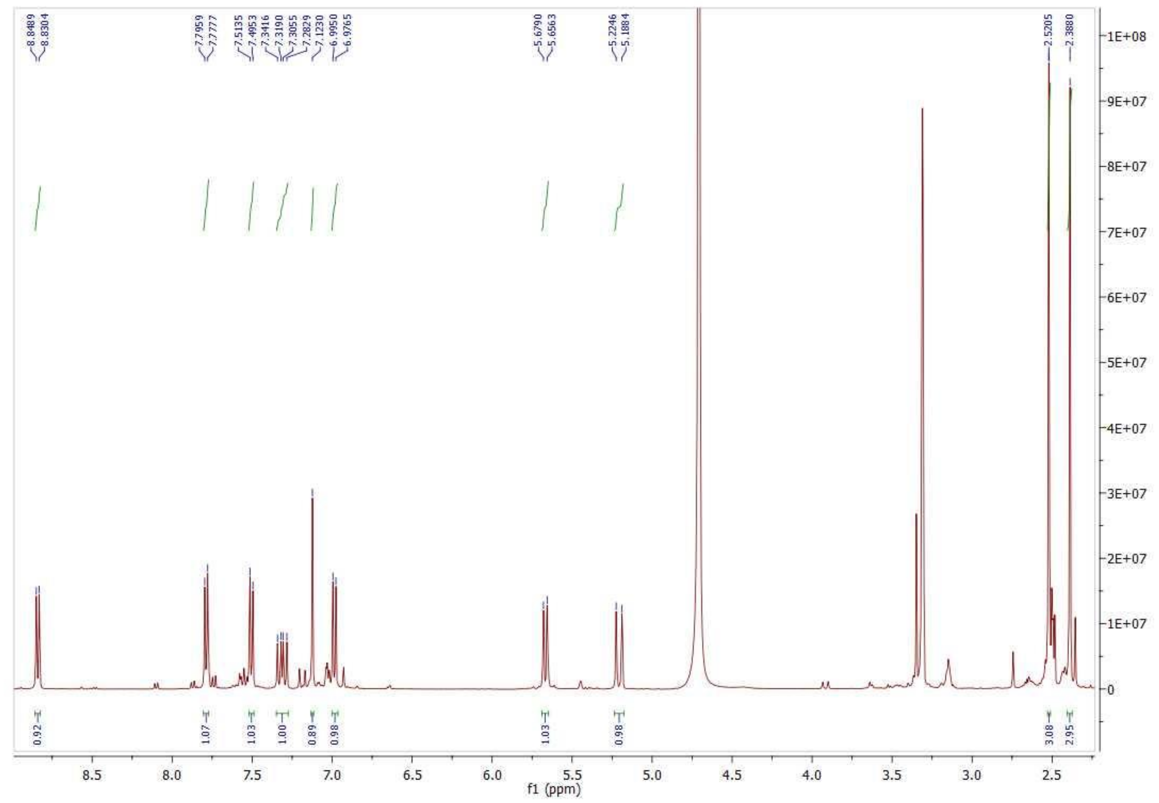

Figure S42. ^{1}H NMR spectrum of compound **7** (500 MHz, in CD_3OD).

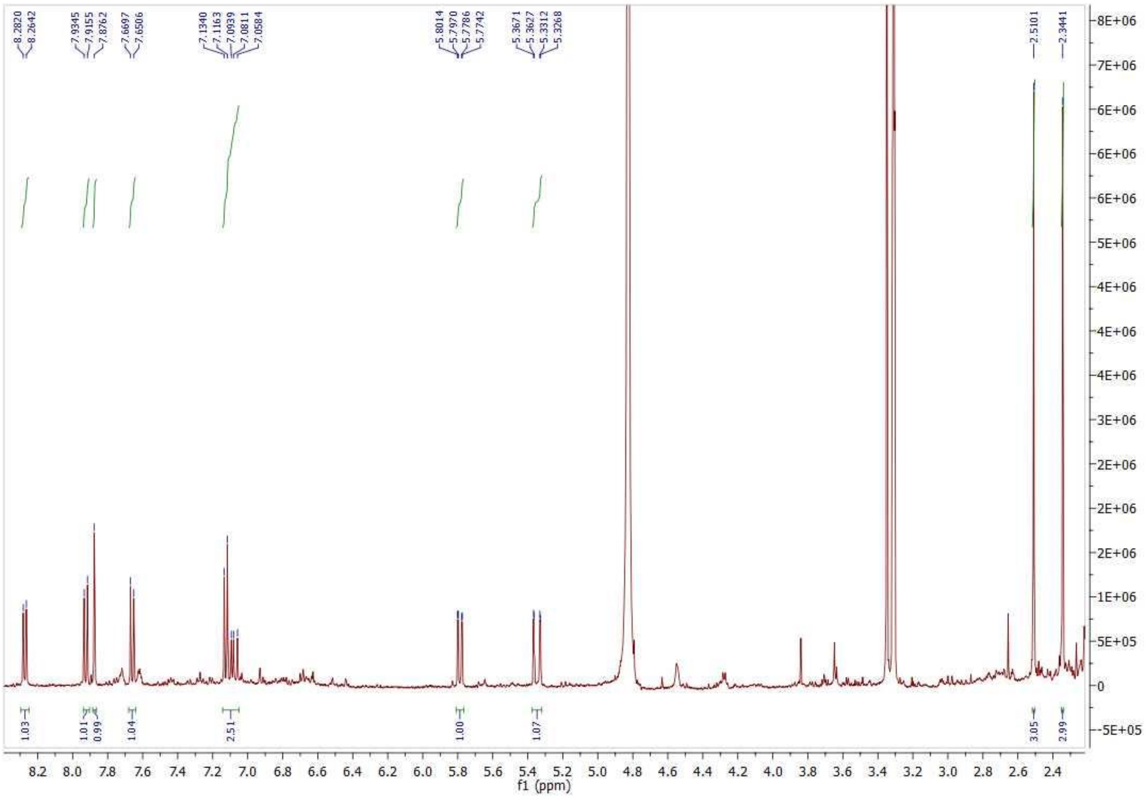

Figure S43. ^{1}H NMR spectrum of compound **8** (500 MHz, in CD_3OD).

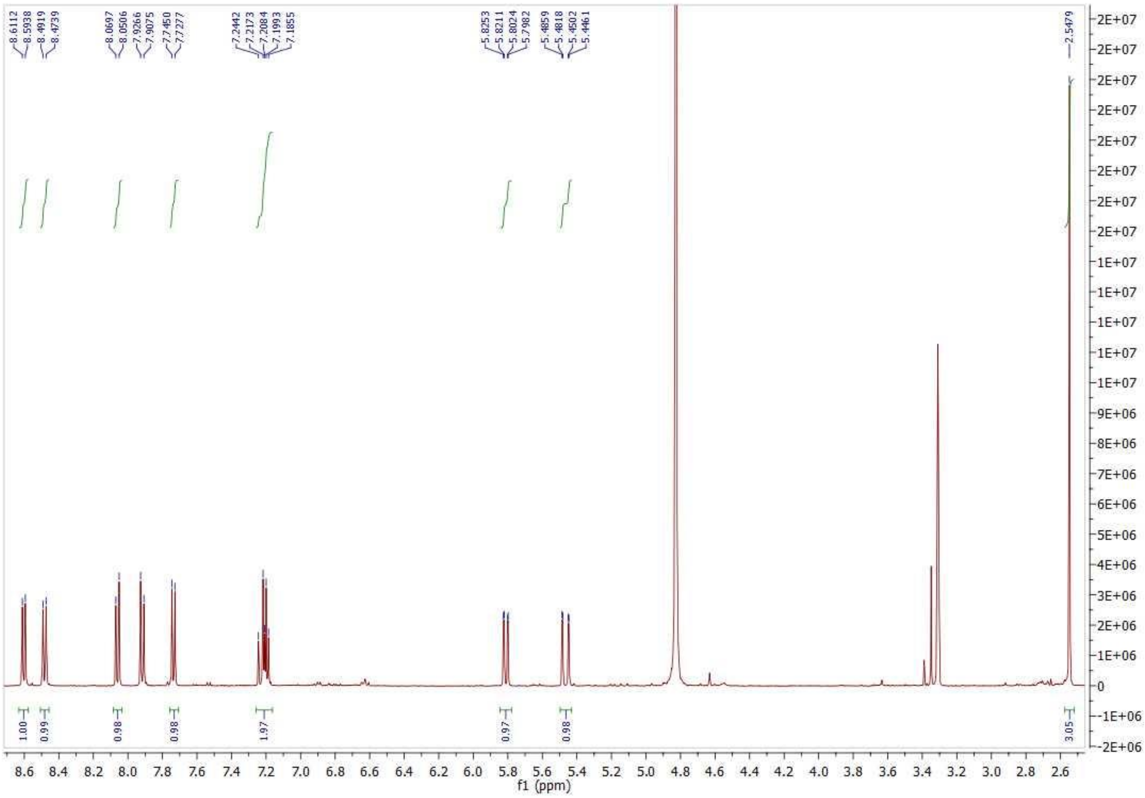

Figure S44. ^{1}H NMR spectrum of compound **9** (500 MHz, in CD_3OD).

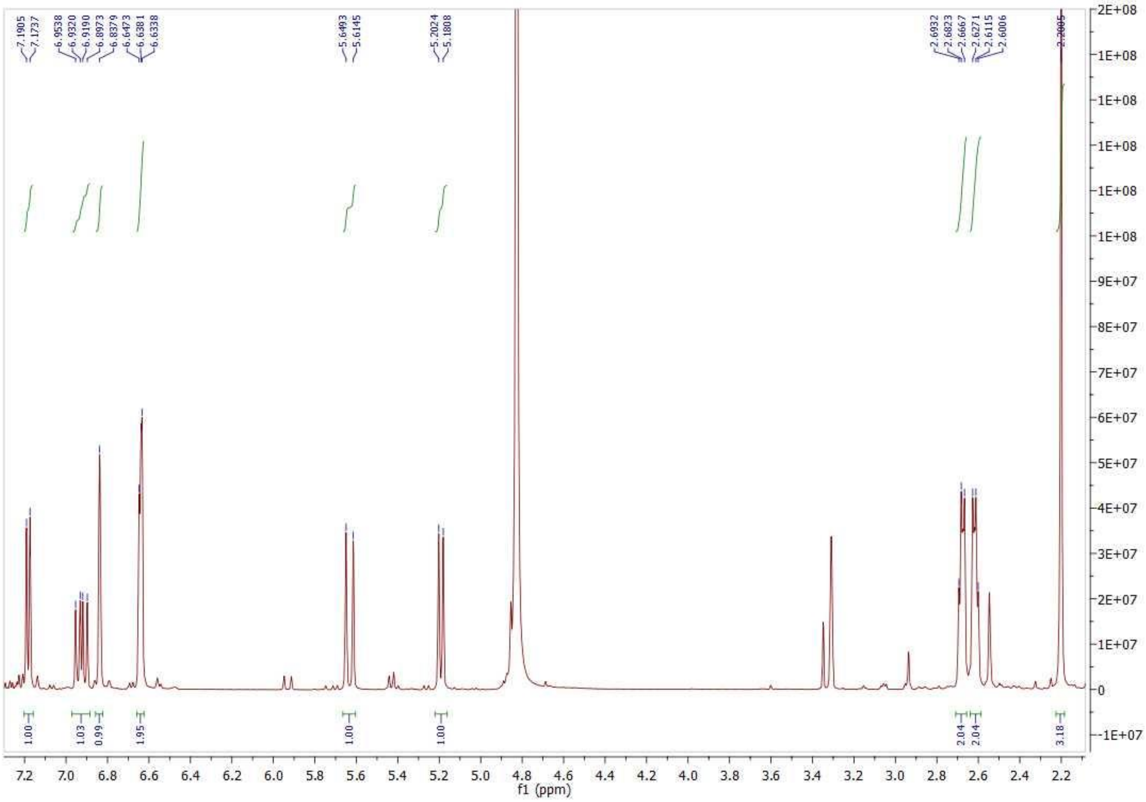

Figure S45. ^{1}H NMR spectrum of compound **10** (500 MHz, in CD_3OD).

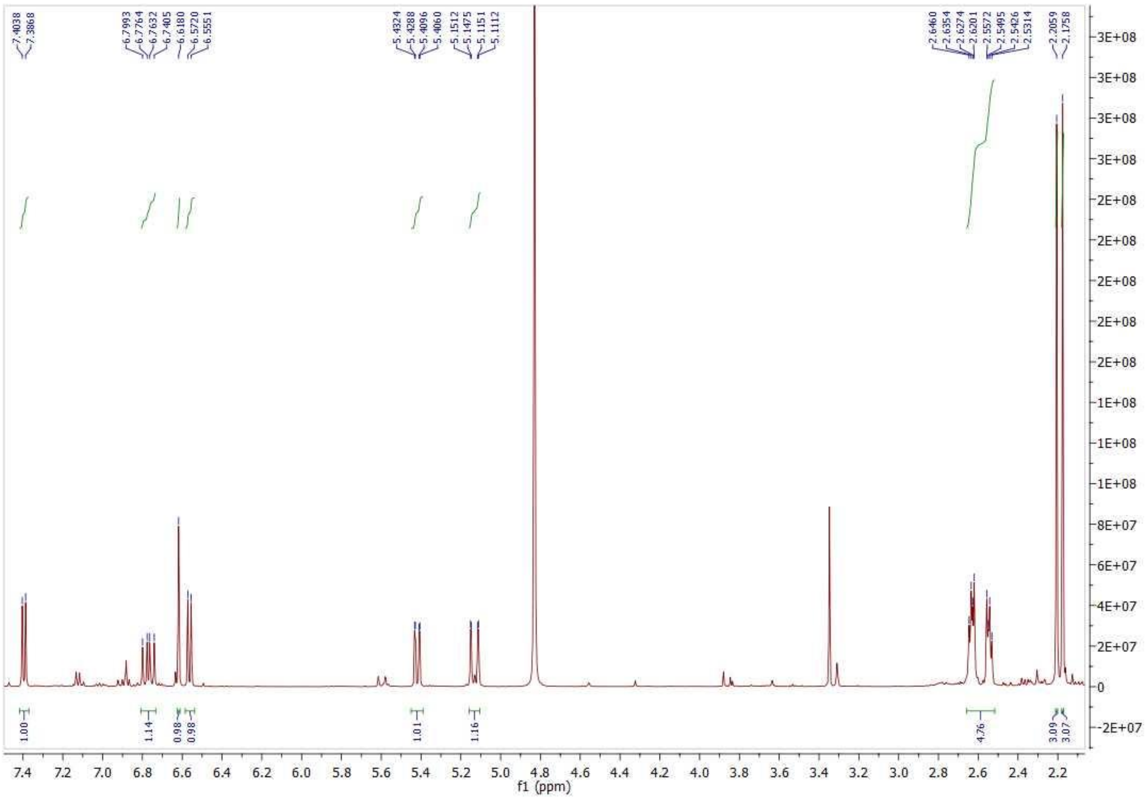

Figure S46. ^{1}H NMR spectrum of compound **11** (500 MHz, in CD_3OD).

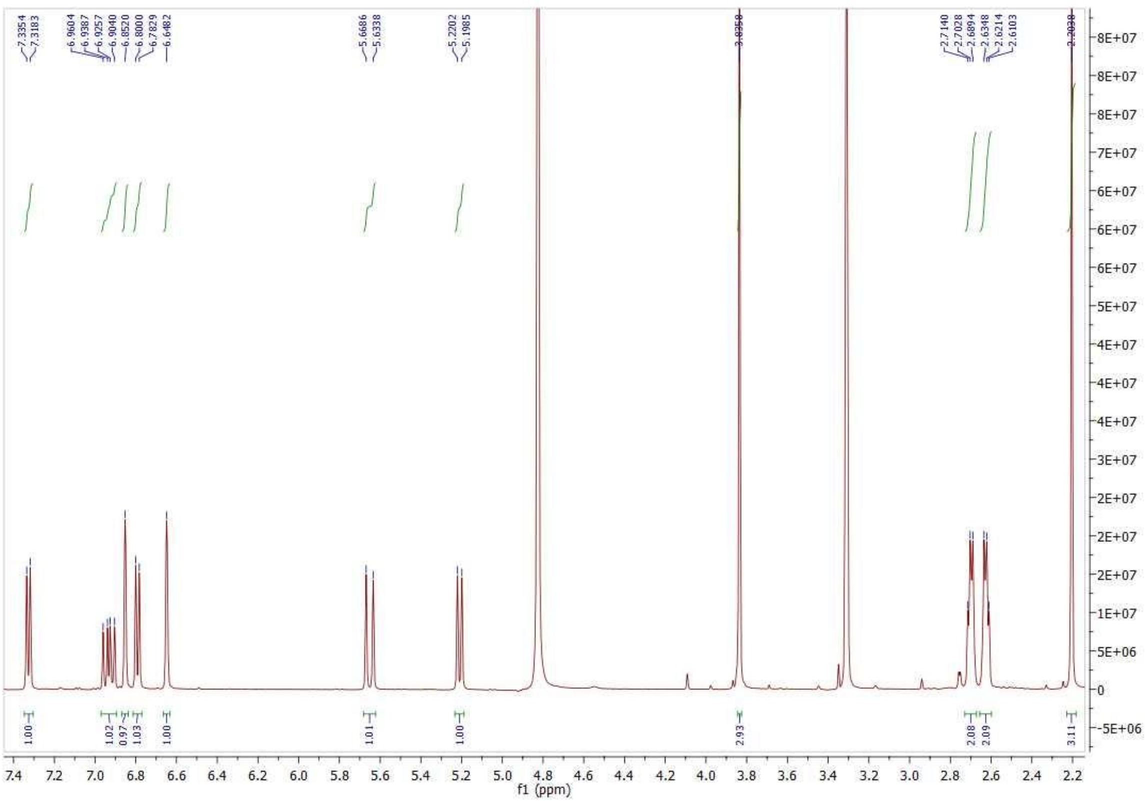

Figure S47. ¹H NMR spectrum of compound **12** (500 MHz, in CD_3OD).

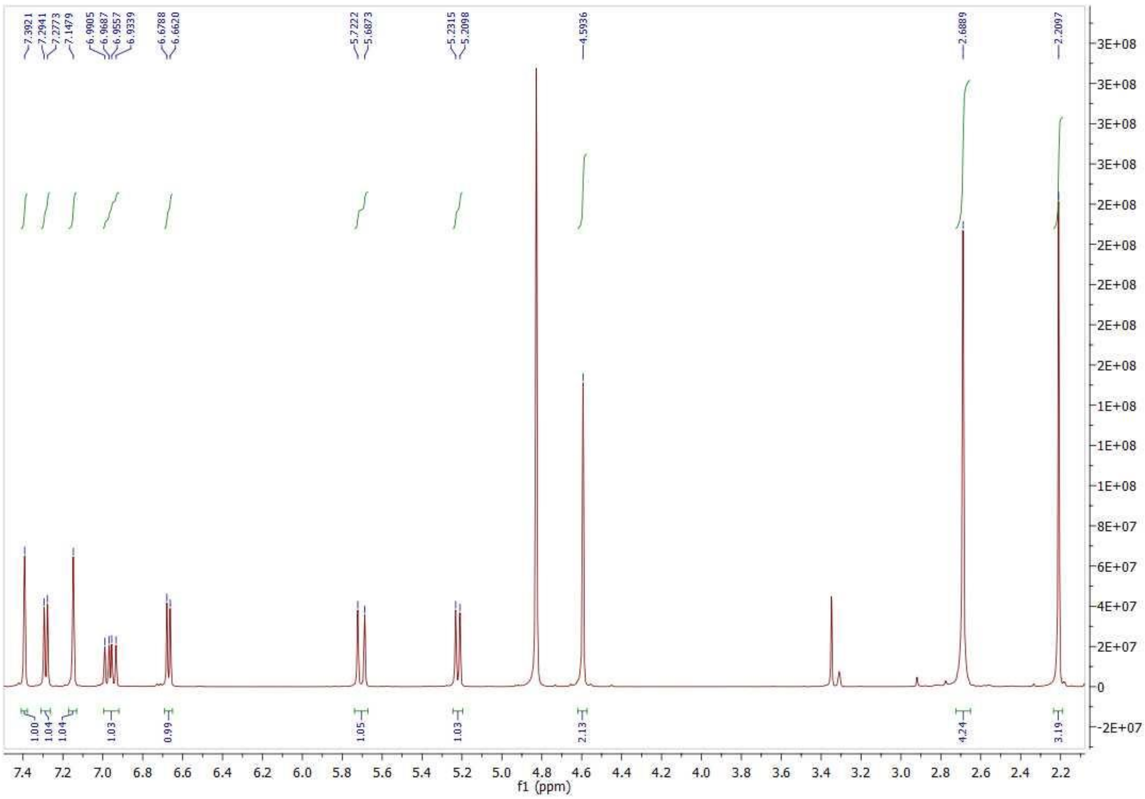

Figure S48. ¹H NMR spectrum of compound **13** (500 MHz, in CD_3OD).

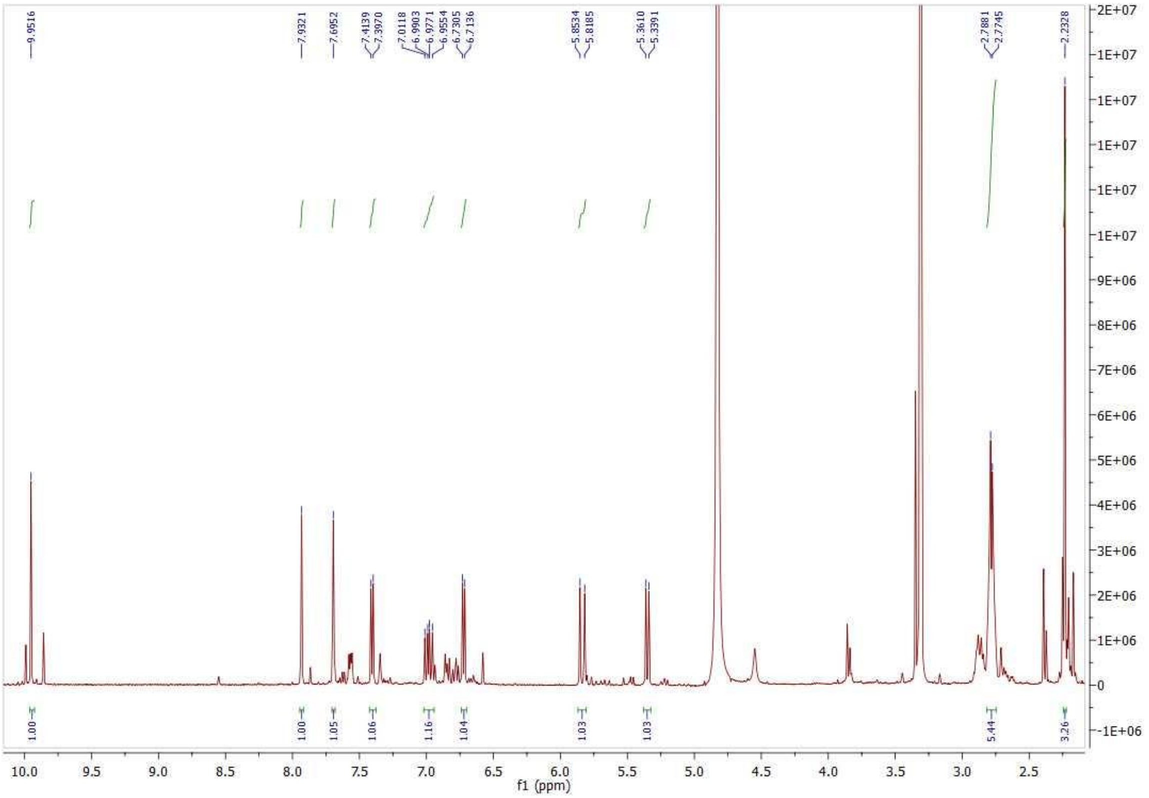

Figure S49. ^{1}H NMR spectrum of compound **14** (500 MHz, in CD_3OD).

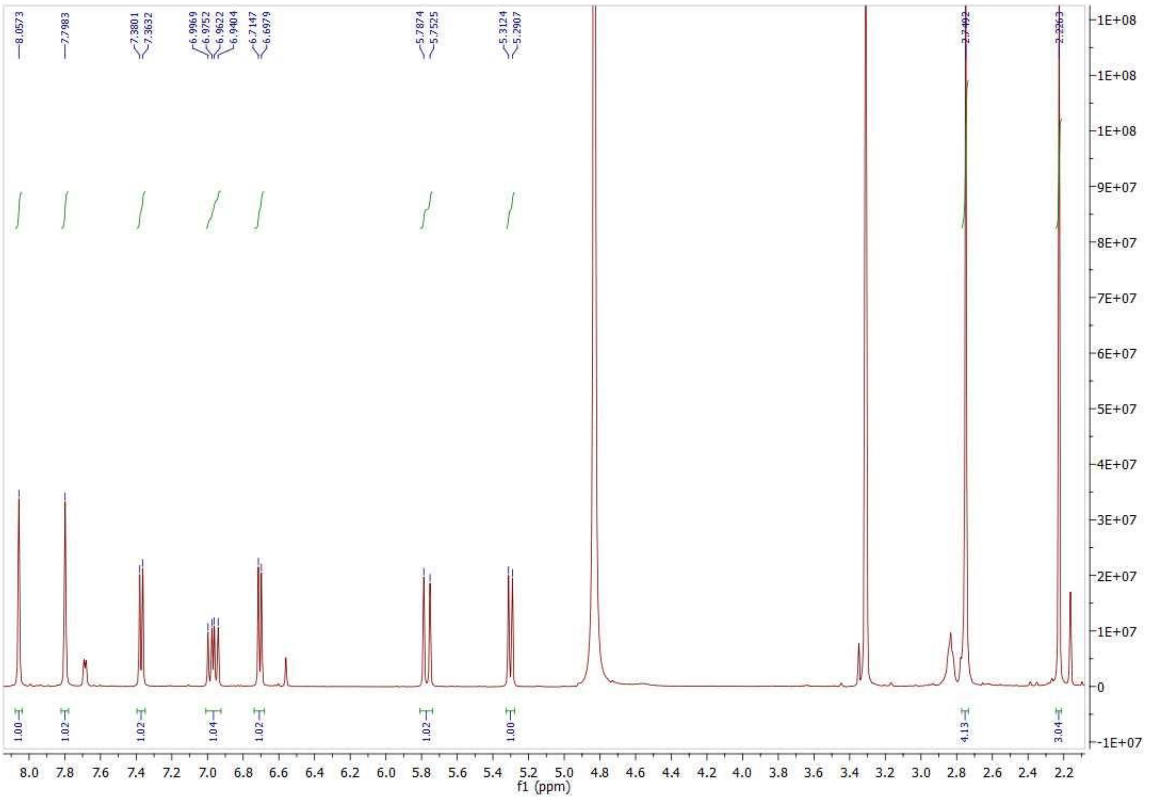

Figure S50. ^{1}H NMR spectrum of compound **15** (500 MHz, in CD_3OD).

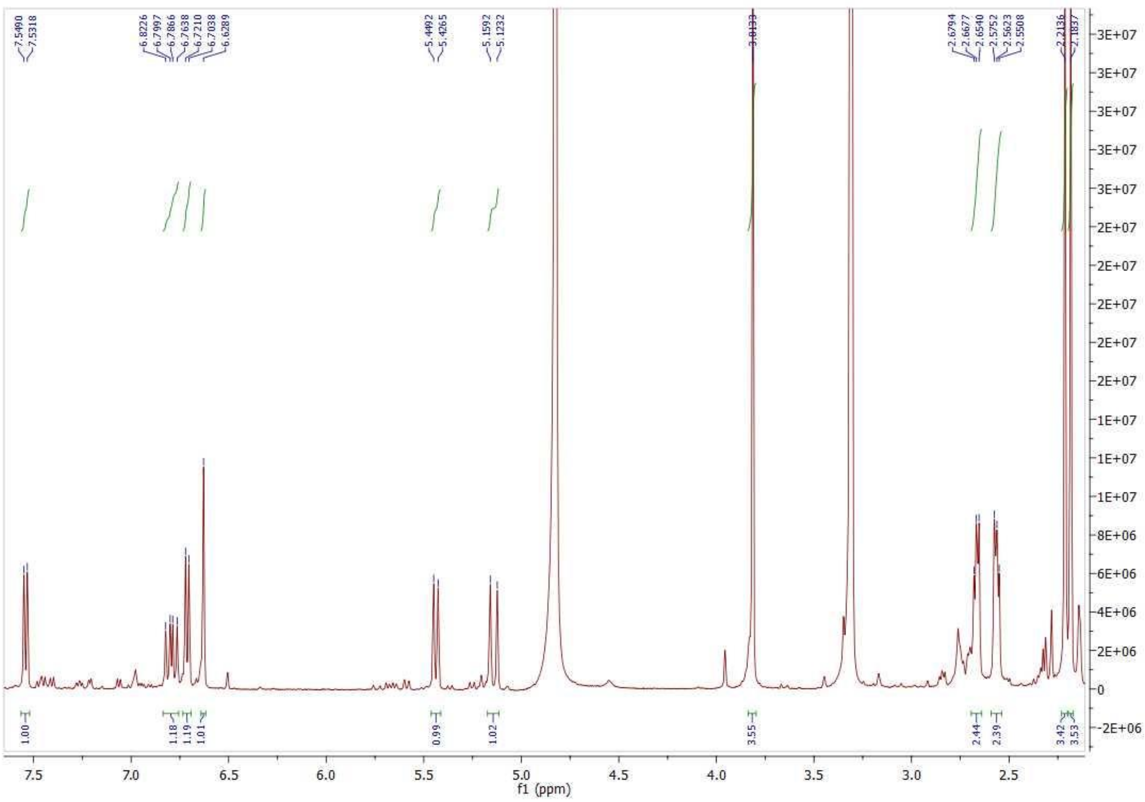

Figure S51. ^{1}H NMR spectrum of compound **16** (500 MHz, in CD_3OD).

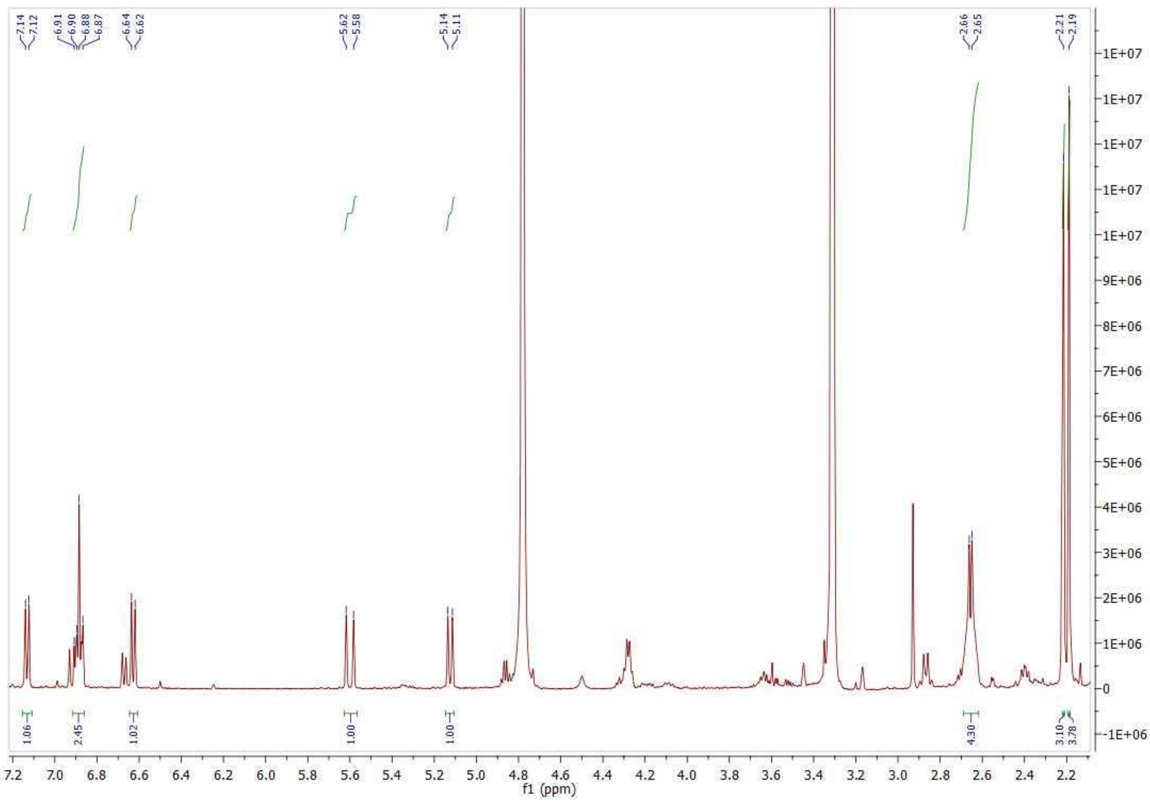

Figure S52. ^{1}H NMR spectrum of compound **17** (500 MHz, in CD_3OD).

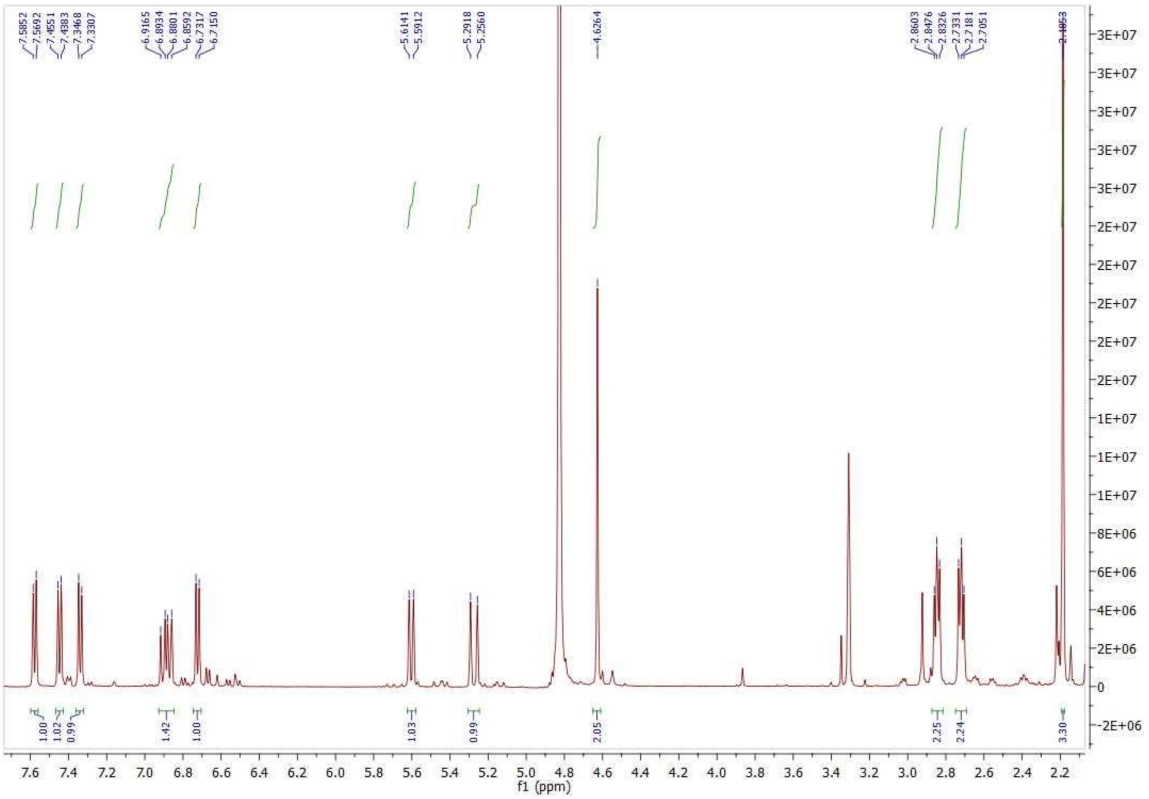

Figure S53. ^{1}H NMR spectrum of compound **18** (500 MHz, in CD_3OD).

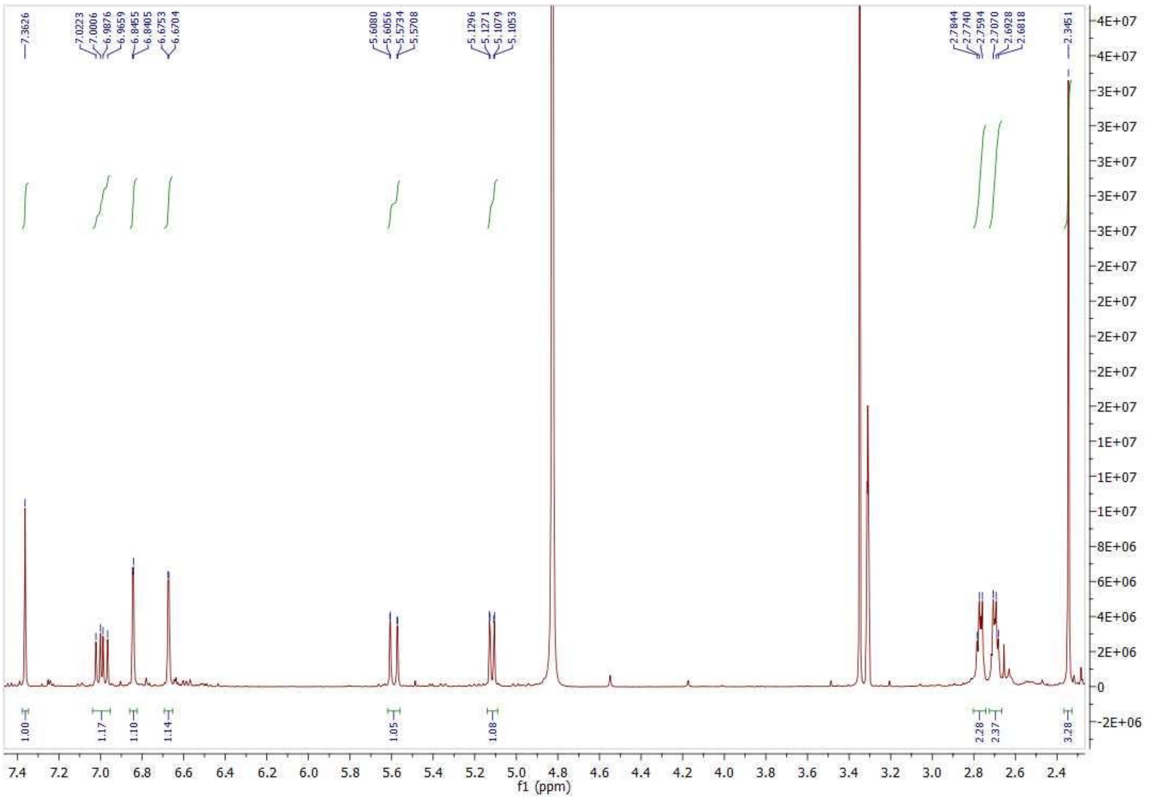

Figure S54. ^{1}H NMR spectrum of compound **19** (500 MHz, in CD_3OD).
